# Semisynthetic Glycoconjugate Vaccine Lead against *Klebsiella pneumoniae* Serotype O2afg Induces Functional Antibodies and Reduces the Burden of Acute Pneumonia

**DOI:** 10.1021/jacs.4c13972

**Published:** 2024-12-12

**Authors:** Dacheng Shen, Bruna M. S. Seco, Luiz Gustavo Teixeira Alves, Ling Yao, Maria Bräutigam, Bastian Opitz, Martin Witzenrath, Bettina C. Fries, Peter H. Seeberger

**Affiliations:** † Department of Bimolecular System, Max Planck Institute of Colloids and Interfaces; 14476 Potsdam, Germany; ‡ Institute of Chemistry and Biochemistry, Freie Universität Berlin, 14195 Berlin, Germany; § Department of Infectious Diseases, Respiratory Medicine and Critical Care, Charite-Universitätsmedizin Berlin; 10117 Berlin, Germany; ∥ German Center for Lung Research (DZL), 12203 Berlin, Germany; ⊥ Department of Medicine, Infectious Disease Division, Stony Brook University; Stony Brook, New York 11794, United States; ⬡ Veteran’s Administration Medical Center, Northport, New York 11768, United States

## Abstract

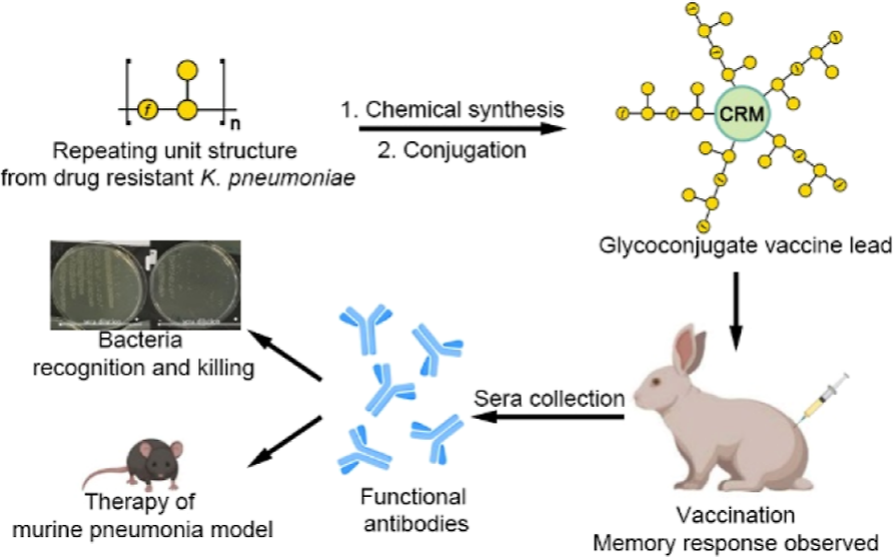

Carbapenem-resistant *Klebsiella pneumoniae* (CR-*Kp*) bacteria are a serious global health concern due to their drug-resistance to nearly all available antibiotics, fast spread, and high mortality rate. O2afg is a major CR-*Kp* serotype in the sequence type 258 group (KPST258) that is weakly immunogenic in humans. Here, we describe the creation and evaluation of semisynthetic O2afg glycoconjugate vaccine leads containing one and two repeating units of the polysaccharide epitope that covers the surface of the bacteria conjugated to the carrier protein CRM_197_. The semisynthetic glycoconjugate containing two repeating units induced functional IgG antibodies in rabbits with opsonophagocytic killing activity and enhanced complement activation and complement-mediated killing of CR-*Kp*. Passive immunization reduced the burden of acute pneumonia in mice and may represent an alternative to antimicrobial therapy. The semisynthetic glycoconjugate vaccine lead against CR-*Kp* expressing O2afg antigen is awaiting preclinical development.

## Introduction

*Klebsiella pneumoniae* is a Gram-negative bacterium naturally occurring in the gastrointestinal tract of humans. Pathogenic strains, when invading other tissues can cause diseases such as pneumonia, urinary tract infection, sepsis, liver abscesses, and meningitis.^1^ Hospital-acquired infections (HAIs) are a critical global health crisis affecting millions annually.^2^ Carbapenem-resistant *K. pneumoniae* (CR-*Kp*) has emerged as a common cause of these infections, with studies reporting mortality rates as high as 30–60%.^3^

The combination of high mortality rate, global dissemination, and their resistance to nearly all currently used antibiotics, resulted in CR-*Kp* to be classified as an urgent public health threat.^4^ A specific CR-*Kp* lineage belonging to the sequencing type 258 (ST258) is the major cause of infections in the USA and Europe,^5−8^ a group that includes the serotype O2afg.^9^ The O2afg serotype is found in over 80% of the CR-*Kp* isolates globally.^10^ The *O-* and *K-* carbohydrate antigens are well-described virulence factors that contribute to *K. pneumoniae* survival by avoiding complement-mediated killing.^11,12^ Both antigens have been explored as targets for glycoconjugate vaccine development due to their prevalent exposure on the bacterial surface.^13^ In contrast to more than 79 *K-*serotypes,^14^ just 12 *O-*serotypes compose the relevant CR-*Kp* clinical strains and among those, four serotypes (O1, O2, O3, and O5) are responsible for more than 90% of all *K. pneumoniae* infections worldwide.^15^ Serotypes O1 and O2 are particularly important as they are associated with high antimicrobial resistance and high prevalence.^16−18^ Although *K. pneumoniae* O2afg strains are markedly more serum sensitive than the O1 strains, humans produce very low numbers of specific B cells against the O2afg antigens. The poor immunogenicity of O2afg antigens may explain the high frequency of CR-*Kp* global propagation of this serotype.^18^

Licensed glycoconjugate vaccines against *Haemophilus influenzae* type B, meningococcus, *Streptococcus pneumoniae*, and *Salmonella typhi*(19,20) are very effectively used to protect humans. *K. pneumoniae* vaccines based on whole cell preparations or subunits^21^ have been studied extensively. A multivalent *O*-antigen glycoconjugate vaccine lead containing O1, O2, O3, and O5 serotypes conjugated to *Pseudomonas aeruginosa* flagellin A or B proteins resulted in high IgG-titers in rabbits against all antigens including the carrier proteins and protected mice against *K. pneumoniae* infection. The vaccine candidate did not enter clinical trials, likely due to the adverse effects caused by the endotoxin lipid A from bacterial cultures.^22^ The Phase I/II study of a tetravalent bioconjugate vaccine candidate (kleb4 V) targeting four *O*-serotypes was completed, but results remain unpublished.^23^

Here, we report the chemical synthesis of two defined *O-*antigen oligosaccharides containing one or two repeating units resembling the O2afg serotype and the immune response of CRM_197_ glycan conjugates in rabbits. Passive immunization with antibodies that target the O2afg serotype glycans was also evaluated in a murine model of acute pneumonia.

## Results

### Synthetic Strategy to Obtain Epitopes Mimicking Natural Occurring O2afg Antigen

**Figure 1:**
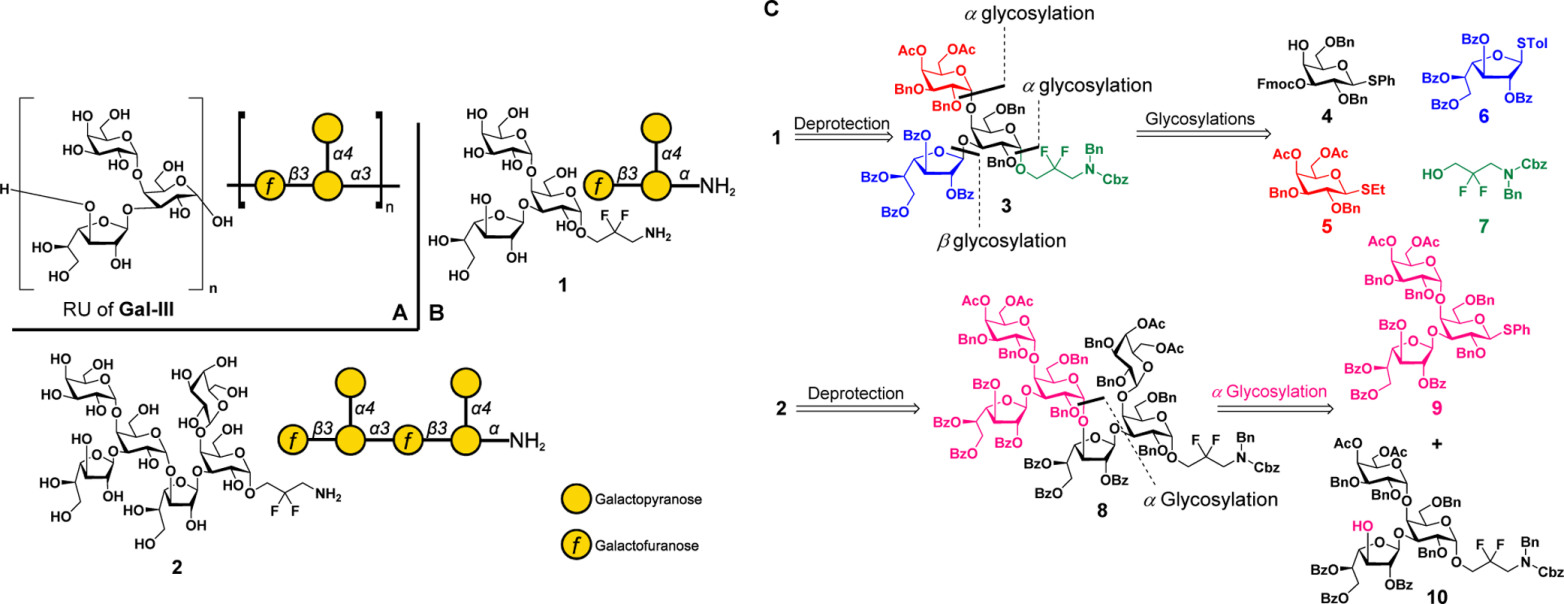
Glycoconjugate vaccine design and retro-synthetic analysis to obtain the epitopes. (A) Trisaccharide repeating unit of **Gal-III** from **KPST258**. (B) Synthetic trisaccharide repeating unit **1** and hexasaccharide **2** containing two repeating units, each connected to an amine linker. (C) Retrosynthetic analysis of trisaccharide **1** and hexasaccharide **2**.

*K. pneumoniae* O2afg serotype bacteria are covered by polysaccharides composed of d-galactan-III (**Gal-III**) (→3)-β-d-Gal*f*-(1 → 3)-[α-d-Gal*p*-(1 → 4)]-α-d-Gal*p*-(1→) repeating units (RUs)^16^ (Figure 1A). We synthesized two oligosaccharide antigens mimicking the natural occurring trisaccharide containing one RU (**1**) and an hexasaccharide containing two RUs (Figure 1B). A fluorinated C_3_ aminoalkyl linker^24^ at the reducing end of both antigens allowed for efficient conjugation to the carrier protein and immobilization on microarrays.

The dense antigen structure with branching at the C3 and C4 positions of the central Gal*p*s to the bridging Gal*f* residue, and the presence of fragile Gal*f* glycosidic bonds renders the synthesis challenging. No syntheses of the *K. pneumoniae* O2afg antigens have been reported so far.

The core galactopyranose of our target trisaccharide **1** connects to the other two sugar residues via C3 and C4 while its anomeric position is connected to a linker. The key strategic consideration is the design of core Gal*p* building block **4**. It needs to be glycosylated with building blocks **5** and **6** and connected to linker **7** on correct positions with proper stereo selectivity. Protected hexasaccharide **8** is assembled by combining two building blocks with trisaccharide-repeating-unit structures together, donor **9** and acceptor **10** (Figure 1C).

### Synthesis of Trisaccharide **1** and Hexasaccharide **2**

The acetyl esters on C4 and C6 of galactopyranose building block **5** participate to ensure α-glycoside formation,^25^ galactofuranose **6**, and 2,2-difluoro-5-aminopropanol linker **7** favoring α-glycosylation^26^ were prepared in anticipation of the assembly of target trisaccharide **1**. Core building block **4** was synthesized from commercially available β-d-galactose pentaacetate (see the Supporting Information for details.).

**Scheme 1:**
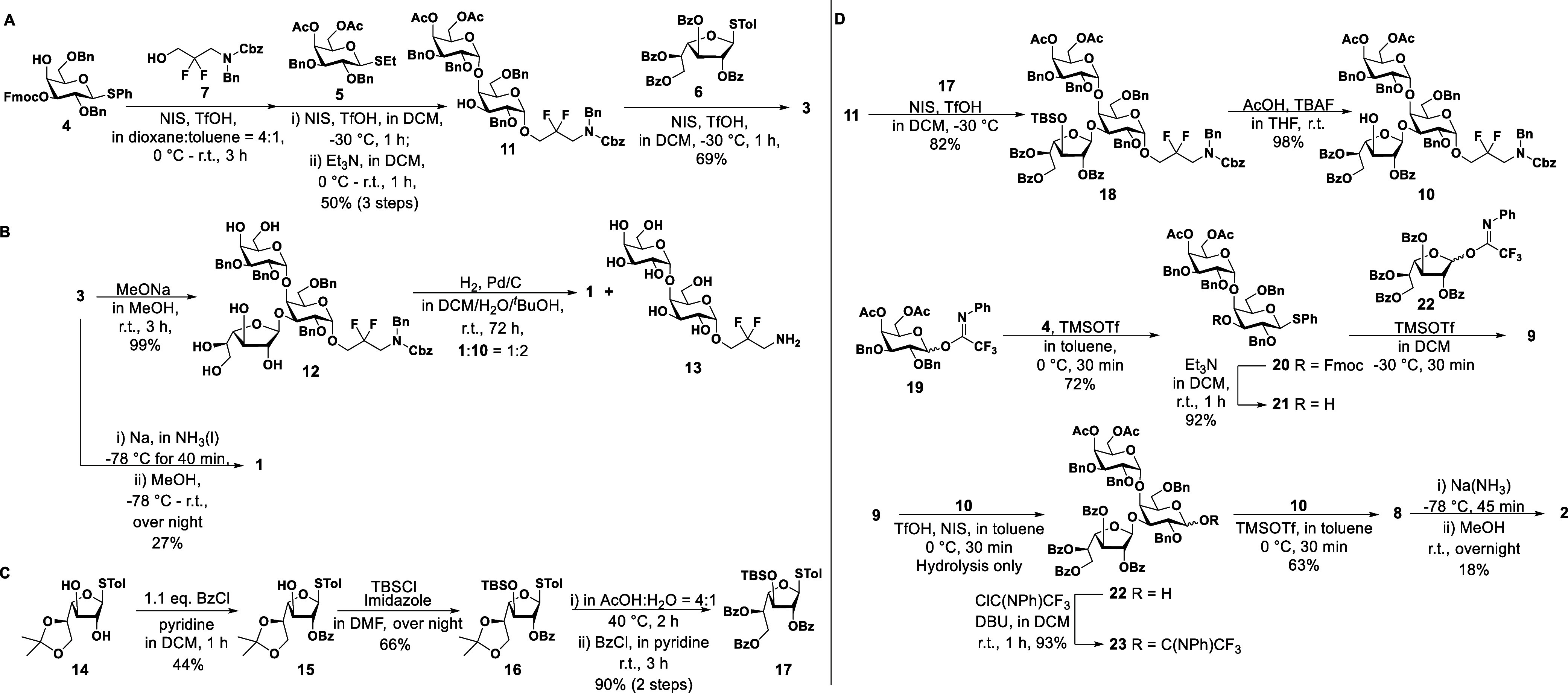
Synthesis of Trisaccharide **1** and Hexasaccharide **2**: (**A**) Assembly of Protected Trisaccharide **3**: (**B**) Global Deprotection of **3** Galactofuranose partially cleaved during Pd catalyzed hydrogenation. With one-pot Birch reduction, trisaccharide **1** was obtained without cleavage of the Gal*f* residue. (**C**) Synthesis of the modified galactofuranose building block **17**. (**D**) Assembly of trisaccharide fragments **9** and **10** for the assembly of hexasaccharide target **2**.

With all of the building blocks in hand, thioglycoside **4** was reacted with linker **7**. Following glycosylation with building block **5** and Fmoc cleavage yielded disaccharide acceptor **11**. The H–C coupling constants (∼170 Hz) of both glycosidic linkages confirmed the formation of two α*-*glycosidic bonds. The β-isomer was not observed by NMR but was detectable by HPLC (α/β = 34:1). The excellent stereoselectivity resulted from the fluorines on the linker and the 4,6-acetyl groups on donor **5**, as well as a relatively high reaction temperature. Glycosylation of disaccharide **11** with Gal*f* donor **6** gave the protected trisaccharide **3** (Scheme 1A).

Global deprotection of trisaccharide **3** via sodium methoxide-driven ester hydrolysis followed by hydrogenation catalyzed by palladium on carbon resulted in partial decomposition of the product through galactofuranose cleavage. In order to avoid the unwanted side reaction, Birch reduction of **3** yielded target trisaccharide **1** in one-pot (Scheme 1B). The NMR spectra of the product were in agreement with those obtained with isolated polysaccharides (see the Supporting Information).

The synthesis of hexasaccharide **2** containing two repeating units was based on the assembly of protected trisaccharide **3**. For that purpose, the C3 hydroxyl group on Gal*f* building block **6** was masked with a temporary protective group that could later be cleaved to reveal the site for extension. Benzoylation of Gal*f***14** gave C2 benzoylated **15** ready for subsequent C3 silylation.^27^ Due to the vulnerability of the isopropylidene group during acidic glycosylation conditions, it was replaced with benzoyl esters at the C5 and C6 positions to obtain building block **17** (Scheme 1C).

Union of building block **17** and disaccharide **11** yielded trisaccharide **18**. Attempts to cleave the TBS ether on the galactofuranose with TBAF resulted in concomitant loss of the acetyl groups. Addition of acetic acid ensured silyl ether cleavage only to furnish trisaccharide acceptor **10** in 98% yield. Trisaccharide donor **9** was synthesized from monosaccharide **4**. Without a linker present at the reducing end, the anomeric thiol leaving group must be retained through subsequent glycosylations. Therefore, thioglycosides **5** and **6** were converted into the corresponding glycosyl trifluoroacetimidates **19** and **22**.^28^ Activation of donors **19** and **22** was achieved with TMSOTf as a promotor instead of TfOH/NIS in order to keep the thiol unaffected (Scheme 1D).

Coupling of trisaccharide donor **9** and acceptor **10** was unsuccessful, yielding acceptor **10** that unreacted and hydrolyzed trisaccharide **23**. Replacing the anomeric thiol by an acetimidate solved a similar problem during the synthesis of a *N. meningitidis* LPS oligosaccharide previously.^29^ Hydrolyzed trisaccharide **23** was recovered and converted into trifluoroacetimidate **24** that was utilized to glycosylate **10** successfully and to obtain protected hexasaccharide **8** in 63% yield. Birch reduction of **8**, desalting and HPLC purification provided hexasaccharide **2**, ready for further conjugation on microarrays or carrier proteins.

### Antibodies from CR-*Kp* Infected Patients Recognize Synthetic O2afg-Antigens

**Figure 2:**
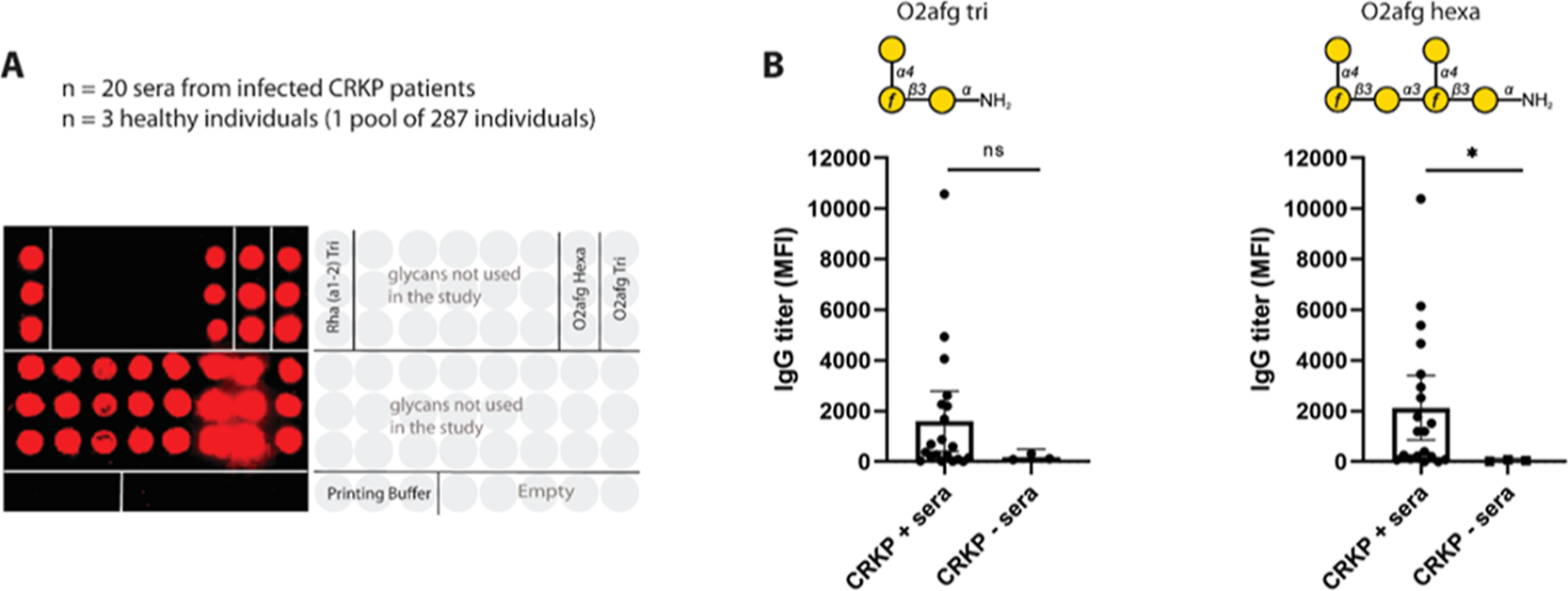
Determination of human antibody binding to synthetic *O*-antigens derived from O2afg serotype. (A) Synthetic glycans **1** and **2** were immobilized on a glass slide and sera from CRK-*Kp* infected patients, and healthy human were incubated with the antigens. Antibody binding was determined by using a glycan microarray assay, and the fluorescence intensity indicates the number of bound IgG antibodies. (B) Graphs showed the titer of bound human IgG antibodies to either O2afg trisaccharide **1** (on the left) or the hexasaccharide **2** (on the right). Both antigens showed positive antibody binders, but only the hexasaccharide showed a significant increase in fluorescence intensity, when compared to the negative control group. A trisaccharide containing the 6-deoxy hexose l-rhamnose (Rha) was used as human antibody positive control. The analysis was performed using the unpaired *t*-test of individual serum mean values from triplicate printed glycan spots. CRKP—carbapenem-resistant *K. pneumoniae*, MFI—mean fluorescence intensity in human sera evaluated by glycan array (left panel), and the mean fluorescence intensity of bound antibodies to O2afg synthetic hexasaccharide from different groups was compared (graph on the right). The sera at time point 0 h was collected as control of preinfection. Two-way ANOVA was used in the analysis. The data represent the mean of MFI values from triplicated printed spots of three individuals per group ±95% CI. **p < 0.01.

Determining the epitope specificity of antibodies is crucial for glycoconjugate vaccine design since the target antigen should generate specific antibodies after immunization.^30^ Humans infected with CR-*Kp* produce antiglycan antibodies that reflect the interaction between the host immune system and the bacterial antigens.^31^ In search of a minimal oligosaccharide epitope, sera of CR-*Kp* infected patients were analyzed using synthetic antigens immobilized on microarrays. Human IgGs produced after infection bound to synthetic trisaccharide **1** and hexasaccharide **2**. Hexasaccharide epitope **2** was significantly better recognized by human antibodies than trisaccharide **1**, when compared to the negative sera control (Figure 2). Thus, hexasaccharide epitope **2** was selected for subsequent vaccination studies.

### Glycoconjugate Vaccine Candidate Preparation and Characterization

Even though humans generate antibodies against the O2afg epitope, the immune response is weak and not long-lasting.^18,32^ The conjugation of poorly immunogenic oligosaccharides to carrier proteins boosts a T-cell-dependent, glycan-specific, and long-lasting immune response.^33,34^ CMR_197_, a nontoxic mutant of diphtheria toxin that has already been used in marketed glycoconjugate vaccines, was selected as carrier protein.^35^ Synthetic O2afg hexasaccharide **2** was conjugated to CRM_197_ by coupling the oligosaccharide to the primary amine side chains of lysine residues and the *N*-terminus of the protein using a *p*-nitrophenyl adipate ester (PNP) as a linker (Figure S1). Conjugation success was confirmed by SDS-PAGE, comparing the shifts of conjugate mass to unconjugated carrier protein, and the estimation of antigen loading (protein/glycan molar ratio) was based on differences of masses between CRM_197_ and CRM_197_-glycan conjugate determined by MALDI-TOF MS. The average antigen loading for **CRM**_**197**_**-2** was 5.2 and was used to calculate the glycan dose for the vaccine (Figure S2).

### Formulated Glycoconjugate Vaccine Evokes the Production of Anti-O2afg Specific Antibodies in Vivo

After **CRM**_**197**_**-2** was formulated with aluminum hydroxide (alum) that is approved for use in humans, the vaccine candidate was used in an immunization study to establish the antibody response in vivo. A very important factor in vaccine development is the choice of animal models. Mice are the most commonly used animal model due to their low cost and straightforward management. Contrary, rabbits are more expensive and demand larger facilities. However, their immune system is evolutionarily closer to the human counterpart. Knowing that the rabbit immune system allows for somatic maturation that is recognized to be an important factor for the successful generation of antiglycan antibodies,^36^ we selected rabbits as our model system for this study.

**Figure 3:**
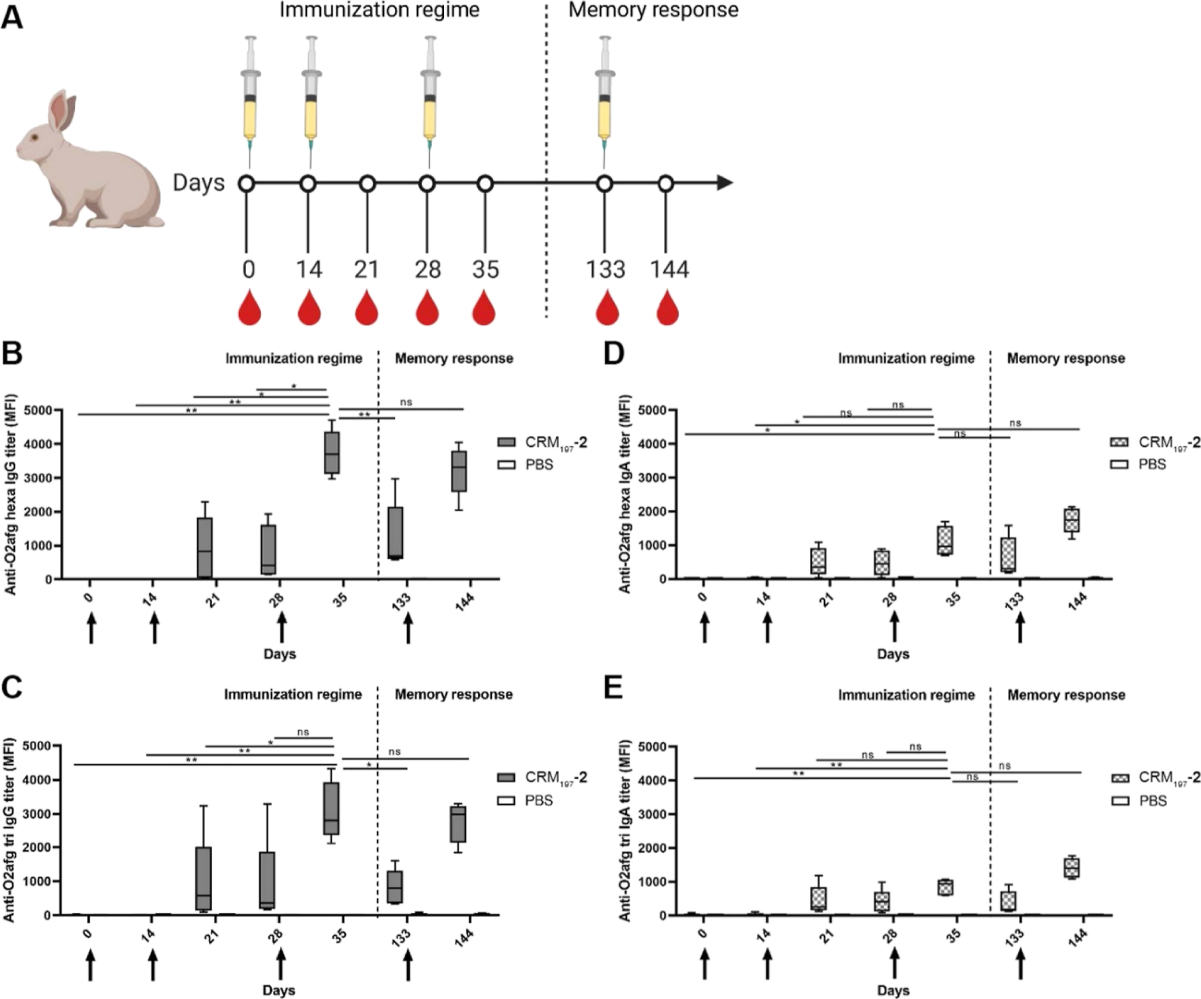
Rabbits immunized with **CRM**_**197**_**-2** produce antibodies that recognize both LPS epitopes **1** and **2**. (A) Schematic schedule for rabbit immunization. Five individuals were immunized on day 0 with **CRM**_**197**_**-2** (1 μg antigen per dose) formulated in alum and boosted on days 14 and 28 with **the** same formulation. The long-term immune response was measured 11 days after a boost injection on day 133. Negative control group containing three individuals received PBS with alum. (B) Levels of IgG specific to the hexasaccharide **2**. (C) Levels of IgG specific against the trisaccharide **1**. (D) Levels of IgA specific to the hexasaccharide **2**. (E) Levels of IgA specific antibody against the trisaccharide **1**. The data represent the 95% CI distribution of five animals in the **CRM**_**197**_**-2** group and three animals in the PBS group. Two-way ANOVA was used for statistical analysis. MFI—mean fluorescence intensity measured by ELISA. *p < 0.05 and **p < 0.01.

The immunogenicity of **CRM**_**197**_**-2** formulated with alum was tested on rabbits. Eight rabbits were divided into two groups. The first group contained five individuals that were immunized intramuscularly (i.m.) with 1 μg of **CRM**_**197**_**-2** vaccine per dose. The second group consisted of three animals that received the same vaccine formulation with PBS instead of the glycoconjugate. The control group contained fewer animals to reduce the use of animals in the experiments since PBS with alum formulations are known to induce no immune response with specific antibodies. The vaccination scheme was based on a well-established three-dose schedule,^37,38^ with a first vaccine dose followed by two boosts with a 14 day interval between each injection. The long-term immune response was measured after a boost injection three and a half months after the last immunization, and the analysis of the antibody response was measured 11 days postimmunization. The antibody titer against the synthetic antigen was measured by ELISA (Figure 3A).

The results showed that the glycoconjugate formulation with **CRM**_**197**_**-2** induced a robust immune response in rabbits with increased IgG titer after the first boost (day 21). A significantly higher antibody titer was achieved after the second boost (day 35). After a resting period, the animals were immunized on day 133 again with the same vaccine formulation. The concentration of antigen-specific IgGs in the serum was rapidly restored, reaching the same level, observed after the second boost (day 35), within 11 days postimmunization (day 144) (Figure 3B). This rapid increase in the antibody level indicated the pre-existence of memory B cells, which were restimulated with the antigen, promptly differentiated into plasma cells and secreted large quantities of antibodies.^39^ The anti-O2afg antibodies bound to both synthetic antigens, confirming that the O2afg trisaccharide **1** is the minimal repeating unit recognized by the immune system^40^ (Figure 3C). Interestingly, the glycoconjugate vaccination also induced the production of specific IgA that recognized both hexasaccharide **2** and trisaccharide **1**, although the levels of generated IgAs were much lower than those of IgGs (Figure 3D,E). The IgA antibody class is important to combat pathogens causing respiratory infection and the residents of human microbiota, as is the case for *K. pneumoniae*.

Native O2afg antigen induces a poor inflammatory immune response, impairing the generation of long-lasting immunity and a robust antibody response.^41^ With the conjugation of the synthetic antigen to a carrier protein, we successfully induced an immune response with high antibody titers, overcoming the lower immunogenicity of the glycan antigen. In summary, 1 μg of conjugated glycan induced a robust immune response in rabbits with the production of specific IgGs and IgAs against the synthetic antigen. Since rabbits have proven to be a better animal model than mice, when translating vaccine response to humans,^36^ our vaccine lead is likely to evoke a strong immune response in humans with the advantage of using a lower dose than marketed vaccines.

Rabbit anti-O2afg IgG antibodies, generated after glycoconjugate vaccination, recognize exclusively the native O2afg antigen and have opsonophagocytic killing activity

The key to immunization with semisynthetic glycoconjugates is to trigger the production of antibodies against specific epitopes. More importantly, these antibodies have to be able to recognize native antigens on the bacterial surface in order to protect the host against an infection caused by the pathogen. To determine whether the rabbit anti-O2afg IgG antibody generated recognize the native O2afg antigen on *K. pneumoniae*, pooled polyclonal sera of rabbits, immunized with **CRM**_**197**_**-2** vaccine from the time point day 35, were incubated with *K. pneumoniae* expressing O2afg antigens. Bacteria cells bound by the rabbit antibody were quantified with a flow cytometer using a fluorescent-labeled antirabbit-IgG secondary antibody. A strain expressing the O1 antigen was used as a negative control.

**Figure 4:**
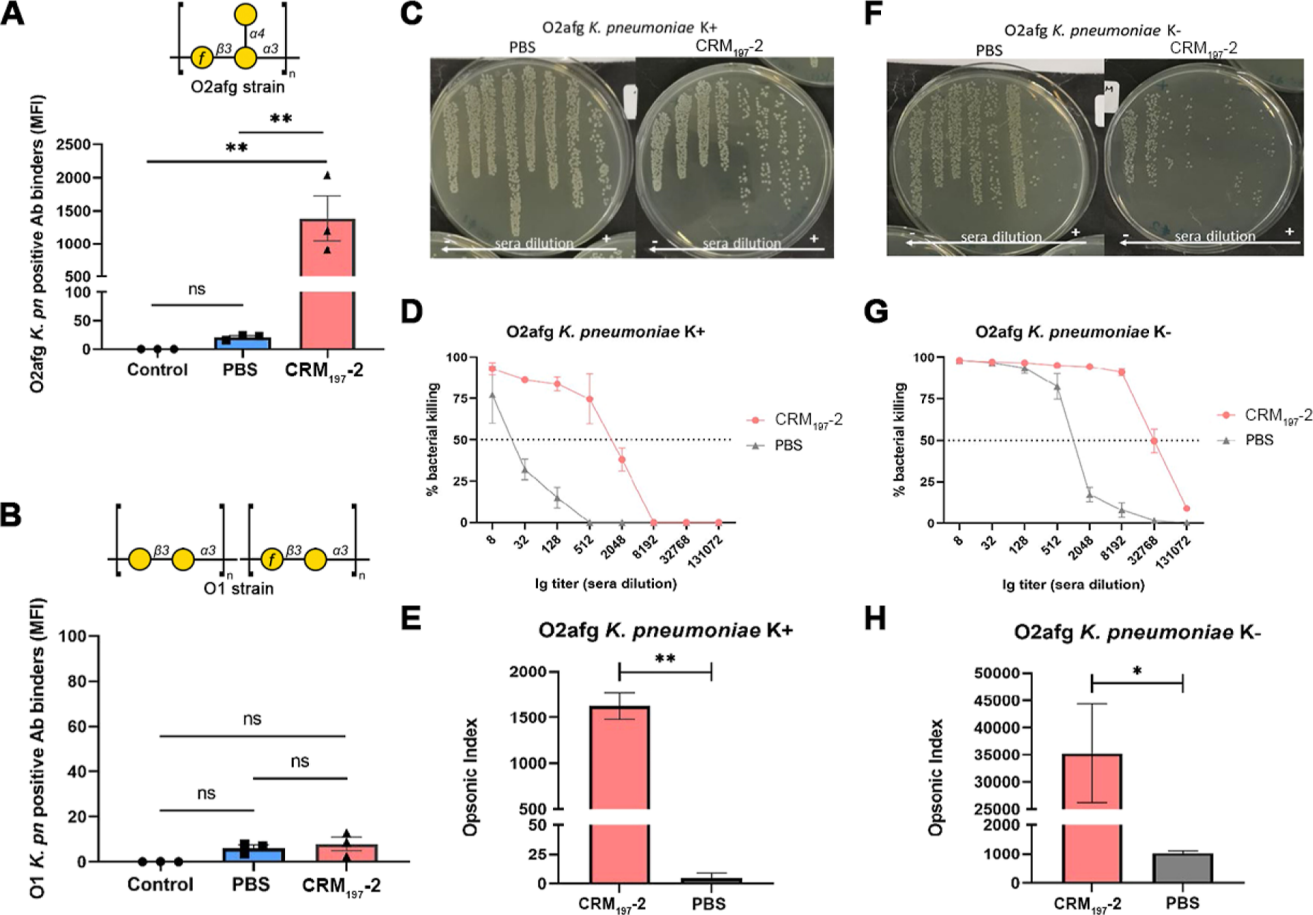
Rabbit anti-O2afg IgG binding assay measured by flow cytometry and opsonophagocytic killing activity. (A,B) *K. pneumoniae* O2afg or O1 were incubated with sera from day 35 (diluted 1:100) from rabbits immunized with either glycoconjugate (**CRM**_**197**_**-2** group, red) or PBS (PBS group, blue). A negative control containing bacteria with fluorescent secondary antibody was used as reference to establish the threshold for positive binding (Figure S4). The positive bind quantification shown in the right panel (MFI) was based on the product of the percent of gated events that passed the fluorescence threshold and the median fluorescence of those events that passed the threshold. MFI—mean fluorescence intensity, *K. pn*—*K. pneumoniae*. The error bars represent the SD of three independent experiments. One-way ANOVA was used for statistical analysis. **p < 0.01. (C–H) Killing activity of antibodies present in the sera of rabbits was evaluated with *K. pneumoniae* expressing a capsule (C,D) and a mutant strain without capsule (F,G). The opsonic index represents the sera dilution for 50% of bacteria killing (E,H) and was based on a four-parameter linear regression of sera dilution curves from two independent assays performed in duplicate. The percentage bacterial killing data (D,G) were mean ± SD of colony-forming units (CFUs) reduction relative to negative control (sample lacking sera with complement and effector cells) of two independent assays performed in duplicate. Unpaired *t*-test was used in the analysis. *p < 0.05, **p < 0.01.

The results showed that rabbit anti-O2afg IgG antibodies recognized native antigens on the surface of the bacteria, while PBS control group IgGs showed no binding. None of the sera had antibodies bound to unrelated O1 antigen. Importantly, anti-O2afg IgGs from rabbits that received the glycoconjugate vaccine lead did not cross-react with O1 *K. pneumoniae* serotype, indicating that the vaccine induced exclusively IgG antibodies against the O2afg antigen (Figure 4A,B). The RUs of both bacterial serotypes used in the assay share a (→3)-β-d-Gal*f*-(1 → 3)-α-d-Gal*p*-(1→) disaccharide. However, no cross-reactivity was observed with the *K. pneumoniae* O1 serotype, underscoring the significance of the branching structure in conferring specificity.

Antibodies that recognize the O2afg antigen are very rare in patients infected with CR-*Kp*, even though the incidence of the O2afg serotype in the CR-*Kp* group is over 80%. This effect results from the weak activation of the immune system by a lower molecular weight of O2afg antigen, when compared to other serotypes. Consequently, very few specific B cells against this antigen are produced.^18^ To date, there has been no report of antibodies specifically targeting **Gal-III** (O2afg). However, some studies have successfully produced anti-O1 and anti-O2 monoclonal antibodies (mAb) and polyclonal sera using animal models infected with respective *K. pneumoniae* serotypes.^18,42,43^ This is expected as in vitro antibody production relies on isolating specific B cells. The low immunogenicity and frequency of B cells targeting the O2afg antigen significantly hinder antibody generation against this target. Overcoming the inherent low immunogenicity of O2afg, our glycoconjugate elicited antibody production against both the synthetic and native antigen forms. Notably, the low-molecular weight of O2afg likely precluded T-independent B-cell activation during infection. In contrast, our glycoconjugate vaccine lead stimulated T-dependent B-cell activation.^44^ Antibodies targeting specifically CR-*Kp* are crucial as other *K. pneumoniae* strains are commensal gut microbiota. Thus, the generated antibodies selectively eliminate pathogenic CR-*Kp* while sparing beneficial gut bacteria.

To verify the functional efficacy of vaccine-induced antibodies in eliminating the target microorganism, an opsonophagocytic killing activity (OPKA) assay^45^ was conducted. This method is widely employed to assess the efficacy of vaccine candidates by estimating the antibody-mediated killing potential of sera from vaccinated animals or humans,^46^ where serum dilution titers strongly correlate with vaccine potency in inducing protective immunity.^47^ The results showed that our synthetic glycoconjugate vaccine lead elicit opsonic antibodies that kill O2afg *K. pneumoniae* (Figure 4C,D). Antibodies from the group that received the glycoconjugate vaccine lead (**CRM**_**197**_**-2**) had an opsonic index of 1626.5 while the PBS group had a value of 4.5 (Figure 4E). This represents a 99.6% significant increase in killing activity, confirming that the glycoconjugate induces the production of opsonic antibodies. Since *K. pneumoniae* are known to express thick capsular polysaccharides that can affect the interaction of antibodies with *O-*antigens, the effect of the capsule in the antibody binding interaction was evaluated using identical serum and OPKA conditions with a mutant acapsular strain (*K. pneumoniae* K−). Glycoconjugate-induced antibodies were also able to kill the acapsular strain (Figure 4F,G) with a 21 times higher opsonic index (Figure 4H) when compared with the capsular strain (*K. pneumoniae* K+) (Figure 4E). Here, we confirmed that our O2afg glycoconjugate vaccine lead induces the production of anti-O2afg antibodies harboring opsonophagocytic killing activity, hence a great potential to fight CR-*Kp* infections.

### Anti-O2afg Antibodies Passively Transferred into Mice Reduce Burden in an Acute Pneumonia Model

**Figure 5:**
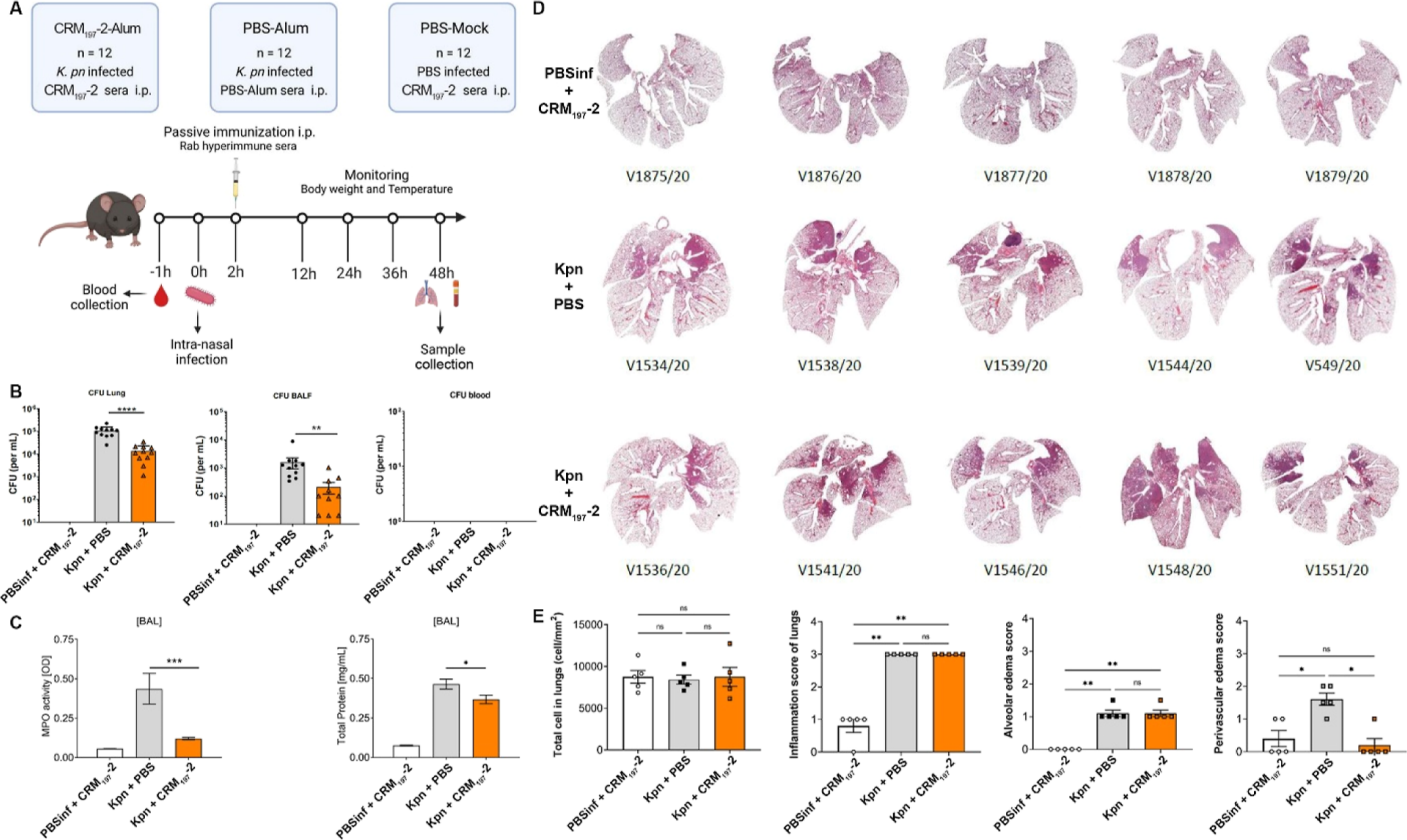
Passive immunization in a murine pneumonia model. (A) Mice were intranasally infected with 1 × 10^8^ of O2afg-CR-*Kp*, and 2 h later passively immunized with polyclonal sera of rabbits vaccinated with either synthetic anti-O2afg vaccine (**CRM**_**197**_**-2**) or PBS vaccine (PBS). Lung, BALF, and blood were collected 48 h postinfection. Mice infected with PBS (PBSinf) but receiving **CRM**_**197**_**-2** polyclonal rabbit sera were used as negative control. (B) Lung (left), BALF (middle), and blood (right) were collected 48 h postinfection and analyzed for bacterial burden by quantifying CFU counting. (C) Myeloperoxidase (MPO) activity and lung permeability were measured in BALF of mice infected with CR-*Kp* 48 h postinfection. Levels of MPO was measured by its enzymatic activity against TMB substrate. Lung permeability was measured by the amount of total protein present in BALF. The data represent the mean ± SE of 12 mice per group. Mann–Whitney was used for the statistical analysis. **p < 0.01 and ****p < 0.0001. One mouse was excluded from the Kpn-infected PBS group due to death after narcosis, and one mouse outlier was excluded from the CR-*Kp*-infected **CRM**_**197**_**-2** group. (D) Lung sections stained with hematoxylin and eosin (H&E) from mice noninfected (PBSInf), mice infected and treated with polyclonal sera of rabbits immunized with O2afg vaccine (Kpn + **CRM**_**197**_**-2**), and mice infected and treated with polyclonal of rabbits vaccinated with PBS (Kpn + PBS), 48 h postinfection. (E) Histopathology scores of the lungs of mice. From left to right: total cells in lungs; inflammation score of lungs; alveolar edema of lungs; and perivascular edema of lungs. The data represent the mean ± SE of five mice per group. Kruskal–Wallis was used for the statistical analysis. *p < 0.05 and **p < 0.01.

To test the protective features of the anti-O2afg antibodies generated by our construct, we administrated antibodies on mice 2 h after infection with CR-*Kp*, as a strategy for passive immunization, terminating the experiment 48 h post infection (Figure 5A). The administration of rabbit antibodies into noninfected mice did not affect their body temperature, indicating that receiving antibodies from different species did not cause a burden for the animal (Figure S5). This result encourages interspecies passive immunization, with rabbits and horses being potential producers of polyclonal sera against infectious diseases.^48^

Pneumonia and sepsis are the primary causes of mortality in patients infected with CR-*Kp*,^49^ with pneumonia being the most frequent cause of sepsis. Consequently, decreasing the number of bacterial colony-forming units (CFUs) in the lungs is crucial for mitigating inflammation and preventing the progression of pneumonia to sepsis. Here, mice that received anti-O2afg polyclonal sera had a significant reduction of more than one-log in bacteria CFU in the lungs and bronchoalveolar lavage fluid (BALF) when compared to the infected group that received polyclonal sera without anti-O2afg antibodies (Figure 5B left and middle). This suggests that the presence of anti-O2afg antibodies was the cause of the bacterial clearance in the lungs and BALF. Therefore, the observed reduction of CFU in BALF was a very important factor to limit the spread of CR-*Kp* in the body. The presence of bacteria in the alveolar space (measured in BALF) may lead to an exacerbated inflammation of the lung tissue, followed by epithelial cell damage and a breach of the alveolar epithelial barrier, possibly facilitating bacterial entry into the bloodstream. However, blood analysis of infected mice revealed no bacteria in the bloodstream across all groups (Figure 5B right), suggesting that the clinical CR-*Kp* isolate used in this study is of limited invasiveness in mice and therefore insufficient to induce bacteremia.

Myeloperoxidase (MPO) is a cytotoxic enzyme with antimicrobial activity, mainly produced in neutrophil granulocytes. It can also be associated with increased lung permeability due to its cytotoxic nature, contributing to acute pulmonary inflammation and epithelial lung tissue injury.^50^ In order to estimate the protective effect of anti-O2afg antibodies in infected lungs, MPO levels and lung permeability were measured in BALF samples from infected animals after administration of the passive immunization with rabbit antibodies vaccinated with either **CRM**_**197**_**-2** or PBS alum. Anti-O2afg antibodies significantly reduced the levels of MPO present in the lungs of infected mice as well as lung permeability, compared to the group that received PBS polyclonal sera (Figure 5C). Immune cell counts and cytokine production were also assessed in blood and BALF samples. The passive immunization with anti-O2afg antibodies led to an increase in the number of immune cells in blood, including neutrophils, monocytes, and eosinophils. Contrarily, the number of neutrophils and inflammatory monocytes, directly related to the increase in lung tissue damage, were significantly lower than that in the control infected group. The number of alveolar macrophages was similar among these two groups (Figure S6). It was reasonable to think that a reduced MPO expression might be correlated with the reduced number of neutrophils infiltrating the lung space, as seen by flow cytometry, as they are the main cell type responsible for MPO production. The production of inflammatory cytokines was also affected by the passive immunization with anti-O2afg antibodies, with a significant increase in the level of IL-12_p70, IL-6, CXCL1, and IFN-γ in blood, when compared to the other infected group. In BALF, a significant increase was only seen for IL-12_p70 and IL-6, while levels of IL-17α were reduced after treatment with anti-O2afg antibodies, comparing to the other infected group (Figure S7).

Histopathology has been a cornerstone method for assessing morphological changes in lung infection models for decades. Relying on qualitative diagnoses of microscopic tissue alterations, it involves certified pathologists applying a semiquantitative scoring system to compare lesion severity between treatment and control groups.^51,52^ Compared with uninfected controls, the lungs of infected mice exhibited increased inflammation and edema, confirming the development of acute lung injury (ALI) (Figure 5D). However, no significant difference in total cell count was observed between groups. While anti-O2afg polyclonal sera did not reduce inflammation scores or alveolar edema compared with PBS controls, it did significantly ameliorate perivascular edema in infected mice, reaching levels comparable to uninfected animals (Figure 5E).

Edema is a hallmark of ALI, resulting from increased capillary permeability caused by endothelial barrier damage, which allows fluid to accumulate in perivascular spaces.^53,54^ In *K. pneumoniae*-induced ALI, lung injury is correlated with elevated neutrophil counts and myeloperoxidase activity.^55^ The reduced neutrophil and MPO levels observed in the BALF of mice treated with anti-O2afg antibodies together with a reduction in the cell counts of inflammatory monocytes may therefore explain the attenuation in tissue damage, as seen by reduced perivascular edema formation and the protein infiltration in the alveolar space of infected mice. Previous studies have demonstrated that decreased neutrophil and MPO levels in the BALF are associated with improved outcomes in influenza-induced acute respiratory distress syndrome and cystic fibrosis.^56,57^ Consequently, the beneficial effects of anti-O2afg antibodies on lung and BALF parameters suggest their potential to enhance the survival rates in CR-*Kp*-infected mice.^58−63^

## Discussion

Bacterial infections remain a leading cause of mortality worldwide, disproportionately affecting immuno-compromised and hospitalized individuals as well as the elderly. The emergence of antimicrobial-resistant strains, such as carbapenem-resistant *K. pneumoniae* (CR-*Kp*), exacerbates this crisis. While antibody-based therapies and vaccine development have been more deeply explored, targeting the highly variable capsular polysaccharide structure has proven challenging due to its limited epidemiological correlations. In contrast, the *O*-antigen, with fewer serotypes and broader coverage, represents a more promising target.

Despite the potential of *O*-antigen-based vaccines, existing approaches using inactivated bacteria or isolated *O*-antigens are hindered by the presence of toxic endotoxins. To address this, we developed a synthetic hexasaccharide mimicking the O2afg serotype, which is a predominant CR-*Kp* strain. Conjugation to the carrier protein CRM_197_ and adsorption to alum created a safe and immunogenic semisynthetic glycoconjugate vaccine lead.

Unlike other *O*-antigens, O2afg exhibits poor immunogenicity, limiting antibody production. However, our vaccine lead successfully elicited a robust immune response in rabbits, generating IgG and IgA antibodies that recognized both the synthetic and native O2afg antigens. These antibodies promoted bacterial clearance through opsonization and complement activation, demonstrating the vaccine’s efficacy. Notably, the low vaccine dose required underscores its potential cost-effectiveness and translational potential.

*K. pneumoniae* is a primary cause of hospital-acquired pneumonia. Critically ill patients, in particular, are particularly susceptible to infections caused by this bacterium. Our study demonstrated the therapeutic effects of anti-O2afg antibodies in murine pneumonia, reducing bacterial burden, inflammation, and lung injury.

## Conclusions

In summary, we have developed a novel, semisynthetic glycoconjugate vaccine lead targeting O2afg-CR-*Kp*, a significant step toward addressing the urgent threat of antimicrobial resistance. The vaccine’s ability to induce a protective immune response and overcome the challenges associated with traditional vaccine approaches holds promise for future clinical development and broader application against resistant pathogens.

## Supporting Information Available

Experimental methods and characterization, including synthesis of the compounds, glycan microarray studies, conjugation, with in vitro and in vivo assays to evaluate the vaccine lead (PDF)
^1^H-, ^13^C-, and HSQC-NMR spectra for new compounds (PDF)
