# Supplementary material for: Semisynthetic Glycoconjugate Vaccine Lead against Klebsiella pneumoniae Serotype O2afg Induces Functional Antibodies and Reduces the Burden of Acute Pneumonia
Source: J Am Chem Soc. 2024 Dec 12;146(51):35356–66. doi: 10.1021/jacs.4c13972 (PMC11673581; doi:10.1021/jacs.4c13972)
Supplement: Supplementary file 1 — ja4c13972_si_001.pdf [file ja4c13972_si_001.pdf]

Supplementary Materials for

A semi-synthetic glycoconjugate vaccine lead against *Klebsiella pneumoniae* serotype O2afg induces functional antibodies and reduces the burden of acute pneumonia

Dacheng Shen, Bruna M. S. Seco, Luiz Gustavo Teixeira Alves, Ling Yao, Maria Bräutigam, Bastian Opitz, Martin Witzenrath, Bettina C. Fries, Peter H. Seeberger*

*Corresponding author. Email: peter.seeberger@mpikg.mpg.de (P.H.S.)

This file includes:
Materials and Methods
Figs. S1 to S7
Table S1

1. General Procedure 3
2. Syntheses of the building blocks 3
3. Assembly of the building blocks 6
4. Deprotections 14
5. Glycan microarrays 15
6. Minimum glycan epitope screening 16
7. Conjugation 16
8. Glycoconjugate formulation with aluminum adjuvant 18
9. Rabbits' immunization and ethical approval 18
10. ELISA analysis of blood samples 19
11. Flow cytometer for bacteria-surface-specific antibody binding 19
12. *In-vitro* opsonophagocytic killing assay (OPKA) 20
13. Passive immunization in murine pneumonia model 21
13.1. Rabbit hyperimmune sera preparation 21
13.2. Bacteria strain characterization and infective sample preparation 21
13.3. Mice and ethical approval 22
13.4. *In vivo* murine pneumonia model 23
13.5. Measurements of immune cells in blood and BALF 25
13.6. Cytokine and chemokine quantification 26
13.7. Permeability assay 27
13.8. Myeloperoxidase (MPO) activity assay 27
13.9. Histopathology 27
14. References 28

1. General Procedure

Commercially available reagents were used without further purification except indicated. All batch reactions were conducted under N_2/Ar atmosphere. ^{1}H-NMR and ^{13}C-NMR spectra were measured with an Ascend 400-MHz, Agilent 400-MHz or Ascend 700-MHz spectrometer. The proton signal of residual, non-deuterated solvent (δ 7.26 ppm for $CHCl_3$; δ 4.79 ppm for H_2O) was used as an internal reference for ^{1}H spectra. For ^{13}C spectra, the chemical shifts were reported relative to the respective solvent (δ 77.16 ppm for $CDCl_3$). Coupling constants were reported in Hertz (Hz). The following abbreviations are used to indicate the multiplicities: s, singlet; d, doublet; t, triplet; m multiplet. Infrared (IR) spectra were recorded as thin films on a Perkin Elmer Spectrum 100 FTIR spectrophotometer. Optical rotations (OR) were measured with a Schmidt & Haensch UniPol L 1000 at 589 nm and a concentration (c) expressed in g/100 mL. High-resolution mass spectra (HRMS) were recorded with an Agilent 6210 ESI-TOF mass spectrometer. MALDI-TOF spectra were recorded on a Bruker Daltonics Autoflex Speed, using a 2,4,6-trihydroxyacetophenone (THAP) matrix. Analytical thin layer chromatography (TLC) was performed on Macherey-Nagel pre-coated TLC plates SIL G-25 UV_{254}. The TLC plates were visualized with UV light and by staining with CAM (ceric sulfate and ammonium molybdate in aqueous sulfuric acid) or sugar stain (2N H_2SO_4 and resorcine monomethylether (0.2%) in ethanol). Column chromatography was performed using silica gel 60 (230–400 mesh). Size exclusion chromatography (SEC) was performed using Sephadex® LH-20 and G-25 (GE Healthcare).

2. Syntheses of the building blocks

4,6-Di-*O*-acetyl-2,3-di-*O*-benzyl-*α*/*β*-D-galactopyranoside (S-1)

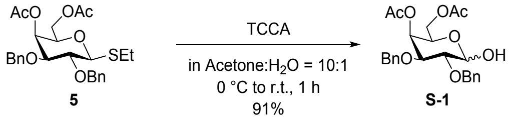

Monosaccharide building block **5** [1] (103 mg, 211 µmol, 1.0 eq.) was dissolved in a mixture of acetone (5 mL) and water (0.5 mL), the solvent was cooled to 0 °C (ice-water bath) and trichloroisocyanuric acid (TCCA) (59 mg, 253 µmol, 1.2 eq.) was added. Ice-water bath was removed and the mixture was stirred at r.t. until TLC showed full conversion of the starting material. The mixture was evaporated *in vacuo* to remove the acetone, extracted with EtOAc, washed with $NaHCO_3$(sat. aq.) and brine, dried with Na_2SO_4(s), concentrated and further purified by flash chromatography (25% to 30% EtOAc in Hexane) to get 85 mg colorless syrup **S-1** (α:β = 5/3, 191 µmol), yield: 91%. ^{1}H NMR (400 MHz, Chloroform-*d*) δ 7.39 – 7.27 (m, 16H), 5.57 (dd, *J* = 3.5, 1.4 Hz, 1H), 5.50 (dd, *J* = 3.1, 1.2 Hz, 1H), 5.27 (d, *J* = 3.6 Hz, 1H), 4.86 (dd, *J* = 11.3, 7.7 Hz, 2H), 4.82 – 4.73 (m, 2H), 4.72 – 4.64 (m, 2H), 4.53 (dd, *J* = 11.2, 8.3 Hz, 2H), 4.36 (ddd, *J* = 7.1, 5.7, 1.4 Hz, 1H), 4.22 – 4.02 (m, 4H), 3.95 (dd, *J* = 9.8, 3.4 Hz, 1H), 3.83 – 3.75 (m, 2H), 3.60 – 3.55 (m, 1H), 3.13 (s, 1H), 2.15 (s, 2H), 2.13 (s, 3H), 2.08 (s, 2H), 2.07 (s, 3H). ^{13}C NMR (101 MHz, $CDCl_3$) δ 170.7, 170.4, 170.4, 138.3, 138.0, 137.8, 137.6, 128.5, 128.4, 128.4, 128.1, 128.1, 128.1, 128.0, 128.0, 127.9, 127.8, 97.4, 92.0, 79.7, 79.1, 77.4, 77.3, 77.1, 76.8, 75.6, 75.5, 75.4, 73.9, 72.2, 72.0, 71.0, 67.5, 66.9, 66.5, 62.5, 62.4, 20.9, 20.9. HRMS (Q-Tof): calculated for $C_{24}H_{28}NaO_8^+$ [M+Na]$^+$ 467.1676, found 467.1683 m/z.

4,6-Di-*O*-acetyl-2,3-di-*O*-benzyl-*α*/*β*-D-galactopyranosyl *N*-phenyltrifluoroacetimidate (19)

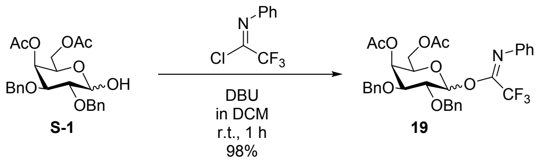

Hydrolyzed monosaccharide **S-1** (155 mg, 349 μmol, 1.0 eq.) and trifluoro-*N*-phenylacetimidoyl Chloride (340 μL, 2.10 mmol, 6.0 eq.) were dissolved in DCM (2 mL), the solvent was cooled to 0 °C (ice-water bath) and DBU (178 μL, 1.05 μmol, 3 eq.) was added. The mixture was stirred at r.t. until TLC showed full conversion of the starting material. After evaporation, the crude product was further purified by flash chromatography (15% EA in Hex) to get yellowish syrup as trifluoroacetimidate donor **19** (α:β = 5/3, 111 mg, 180 μmol), yield 98%. ^{1}H NMR (400 MHz, Chloroform-*d*) δ 7.39 – 7.24 (m, 13H), 7.10 (tq, *J* = 7.4, 1.4 Hz, 1H), 6.79 (d, *J* = 7.7 Hz, 2H), 5.65 – 5.48 (m, 1H), 4.86 – 4.67 (m, 3H), 4.57 (dd, *J* = 21.8, 11.2 Hz, 1H), 4.16 (qd, *J* = 11.4, 6.5 Hz, 2H), 3.85 (t, *J* = 8.7 Hz, 1H), 3.66 (s, 1H), 2.16 (d, *J* = 23.6 Hz, 3H), 2.06 (d, *J* = 21.9 Hz, 3H). ^{13}C NMR (101 MHz, $CDCl_3$) δ 170.5, 170.3, 170.2, 143.4, 138.0, 137.8, 137.7, 137.4, 129.4, 128.8, 128.5, 128.5, 128.4, 128.2, 128.1, 128.0, 128.0, 128.0, 127.8, 127.8, 127.5, 124.3, 119.1, 79.1, 77.4, 77.0, 76.7, 75.8, 75.4, 74.7, 73.8, 72.3, 71.8, 69.4, 67.1, 66.1, 62.1, 61.7, 29.7, 20.9, 20.9, 20.8, 20.7, 1.1. HRMS (Q-Tof): calculated for $C_{32}H_{32}F_3NNaO_8^+$ $[M+Na]^+$ 638.1972, found 638.1980 m/z.

Phenyl 2,6-di-*O*-Benzyl-3-*O*-fluorenylmethoxycarbonyl-1-thio-*β*-D-galactopyranoside (4)

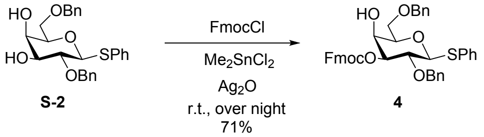

Monosaccharide **S-2** [2] (1.806 g, 3.99 mmol, 1.0 eq.) was put into a flask and Ag_2O (1.017 g, 4.38 mmol, 1.1 eq.) and Me_2SnCl_2 (44 mg, 0.199 mmol, 0.05 eq.) were added. After MeCN (anhydrous, 35 mL) was added as solvent, FmocCl (1.576 g, 6.09 mmol, 1.5 eq.) was added to the suspension and the mixture was allowed to stirr at r.t. over night. According to TLC, the starting material was fully conversed. The mixture was filtrated with Celite and concentrated *in vacuo*. Building block **4** was further purified with flash chromatography to get white solid 1.906 g (2.82 mmol), yield: 71%.^{1}H NMR (400 MHz, Chloroform-*d*) δ 7.77 (d, *J* = 7.5 Hz, 2H), 7.65 – 7.56 (m, 4H), 7.44 – 7.22 (m, 18H), 4.86 (d, *J* = 10.5 Hz, 1H), 4.80 (dd, *J* = 9.5, 3.0 Hz, 1H), 4.70 (d, *J* = 9.7 Hz, 1H), 4.66 (d, *J* = 10.5 Hz, 1H), 4.64 – 4.54 (m, 2H), 4.45 (dd, *J* = 10.5, 7.2 Hz, 1H), 4.37 (dd, *J* = 10.4, 7.4 Hz, 1H), 4.29 (d, *J* = 3.0 Hz, 1H), 4.23 (t, *J* = 7.2 Hz, 1H), 3.93 (t, *J* = 9.6 Hz, 1H), 3.82 (qd, *J* = 10.2, 4.9 Hz, 2H), 3.69 (t, *J* = 4.9 Hz, 1H), 2.81 (s, 1H). ^{13}C NMR (101 MHz, $CDCl_3$) δ 154.5, 143.4, 143.2, 141.4, 137.9, 137.6, 133.3, 132.3, 129.1, 128.7, 128.5, 128.2, 128.1, 128.1, 128.0, 128.0, 127.9, 127.8, 127.3, 127.3, 125.3, 125.2, 120.2, 120.2, 87.8, 80.9, 77.5, 77.4, 77.2, 76.8, 76.5, 75.8, 75.2, 74.0, 70.3, 70.0, 68.4, 46.8. HRMS (Q-Tof): calculated for $C_{41}H_{38}NaO_7S^+$ $[M+Na]^+$ 697.2230, found 697.2234 m/z.

4-Methylphenyl-5,6-*O*-isopropylidene-2-*O*-benzoyl-1-thio-*β*-D-galactofuranoside (15)

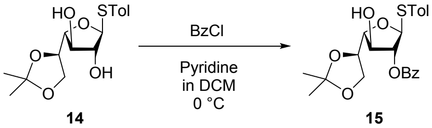

Galactofuranoside **14** (639 mg, 1.96 mmol, 1.0 eq.) was dissolved in DCM (10 mL) and the temperature was cooled to 0 °C with an ice-water bath. Pyridine (0.50 mL, 3.91 mmol, 2.0 eq.) and BzCl (0.25 mL, 2.15 mmol, 1.1 eq.) were added sequentially and the mixture was allowed to stirr at that temperature for 1 h. The mixture was washed with H_2O, 1 M HCl(aq.), $NaHCO_3$(aq.) and brine, dried with Na_2SO_4(s), concentrated and purified by flash chromatography to get benzoylated product **15** (R_f = 0.4 on TLC with a gradient of 30% EtOAc in Hex), 369 mg, 0.86 mmol, yield: 44%. Data of the compound **15** were compromised as reported [3].

4-Methylphenyl-5,6-*O*-isopropylidene-2-*O*-benzoyl-3-*tert*-butyldimethylsilyl-1-thio-*β*-D-galactofuranoside (16)

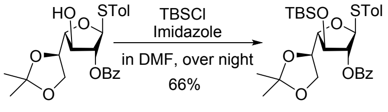

Compound **15** (369 mg, 0.857 mmol, 1.0 eq.) was dissolved in anhydrous DMF (8.0 mL). Imidazole (117 mg, 1.72 mmol, 2.0 eq.) was added to the solution and the mixture was cooled to 0 °C (ice-water bath). TBSCl (194 mg, 1.29 mmol, 1.5 eq.) was added, the cooling bath was removed and the resultant mixture was stirred overnight. The reaction was quenched by MeOH and the mixture was evaporated to remove most of the DMF. After extraction with EtOAc, washed with water and brine, dried with Na_2SO_4(s) and concentrated again, the product was further purified with flash chromatography to get galactofuranoside **16** (304 mg, 0.556 mg, 66%). ^{1}H NMR (400 MHz, Chloroform-*d*) δ 8.17 – 7.94 (m, 2H), 7.66 – 7.55 (m, 1H), 7.52 – 7.38 (m, 4H), 7.10 (d, *J* = 7.8 Hz, 2H), 5.49 (d, *J* = 2.7 Hz, 1H), 5.37 (t, *J* = 2.9 Hz, 1H), 4.39 – 4.26 (m, 2H), 4.22 (dd, *J* = 6.0, 4.5 Hz, 1H), 4.06 (dd, *J* = 8.1, 6.6 Hz, 1H), 3.93 (t, *J* = 7.7 Hz, 1H), 2.31 (s, 3H), 1.43 (s, 3H), 1.39 (s, 3H), 0.90 (s, 9H), 0.09 (d, *J* = 3.0 Hz, 6H). ^{13}C NMR (101 MHz, $CDCl_3$) δ 165.5, 137.8, 133.6, 132.7, 130.8, 130.0, 129.8, 129.4, 128.6, 109.8, 91.2, 84.3, 83.1, 77.5, 77.2, 76.8, 74.8, 65.7, 32.1, 29.9, 26.5, 25.8, 25.7, 22.9, 21.3, 18.0, 14.3, -4.4, -4.9. LCMS (ESI): HRMS (Q-Tof): calculated for $C_{29}H_{40}NaO_6SSi^+$ $[M+Na]^+$ 567.2207, found 567.2220 m/z.

4-Methylphenyl-2,5,6-tri-*O*-benzoyl-3-*tert*-butyldimethylsilyl-1-thio-*β*-D-galactofuranoside (17)

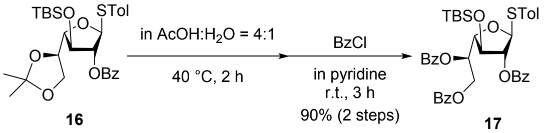

Monosaccharide **16** (251 mg, 0.46 mmol, 1.0 eq.) was dissolved in a mixed solvent of AcOH and H_2O (5 mL, v/v = 4/1) and the mixture was stirred at 40 °C until **13** was fully converted. The time should be controlled to prevent TBS from cleavage. The mixture was then extracted with EtOAc, washed with H_2O, $NaHCO_3$(aq.) and brine, dried with Na_2SO_4(s), concentrated *in vacuo* and used for next step without further purification. The crude product was dissolved in pyridine (5 mL) and BzCl (0.16 mL, 0.69 mmol, 3.0 eq.) was added at 0 °C. After stirring for 1 h, the reaction was quenched with EtOAc, washed with H_2O, HCl (aq., 1 M), $NaHCO_3$(aq.) and brine, dried with Na_2SO_4(s), concentrated and purified by flash chromatography to get colorless syrup as product **17** (397 mg, 0.417 mmol), yield: 90% for two steps. ^{1}H NMR (400 MHz, Chloroform-*d*) δ 8.14 – 8.01 (m, 2H), 8.01 – 7.94 (m, 2H), 7.94 – 7.84 (m, 2H), 7.64 – 7.48 (m, 3H), 7.45 – 7.29 (m, 8H), 7.05 (d, *J* = 7.9 Hz, 2H), 5.86 (ddd, *J* = 7.8, 4.3, 3.3 Hz, 1H), 5.63 – 5.51 (m, 1H), 5.37 – 5.24 (m, 1H), 4.77 – 4.56 (m, 3H), 4.43 (ddd, *J* = 5.9, 2.3, 0.9 Hz, 1H), 2.30 (s, 3H), 0.90 (s, 9H), 0.08 (s, 3H), 0.06 (s, 3H). ^{13}C NMR (101 MHz, $CDCl_3$) δ 166.2, 166.0, 165.6, 138.0, 133.5, 133.5, 133.3, 133.0, 130.3, 130.0, 129.9, 129.9, 129.8, 129.7, 129.6, 129.1, 128.6, 128.5, 91.5, 85.0, 82.8, 77.5, 77.4, 77.2, 76.9, 76.8, 69.9, 63.8, 32.1, 29.9, 29.5, 25.7, 22.9, 21.3, 17.9, 14.3, -4.6, -5.0. HRMS (Q-Tof): calculated for $C_{40}H_{44}O_8SSiNa^+$ $[M+Na]^+$ 735.2418, found 735.2419.

3. Assembly of the building blocks

4,6-Di-*O*-acetyl-2,3-di-*O*-benzyl-*α*-D-galactopyranosyl-(1→4)-2,6-di-*O*-benzyl-*α*-D-galactopyranosyl-(1→1)-(3-*N*-benzyl-*N*-benzyloxycarbonylamino)-2,2-difluoropropanol (11)

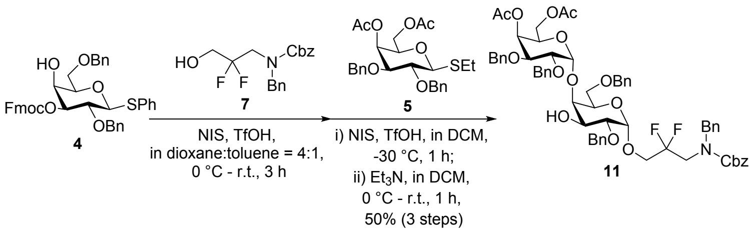

The donor **4** (68 mg, 100 μmol, 1.0 eq.) and the acceptor **7** (40 mg, 120 μmol, 1.2 eq.) were coevaporated with toluene and put under high vacuum to remove the trace water. After dissolved in a mixed solvent (1 mL, 1,4-dioxane:toluene = 4:1), 4 Å AWMS was added and the system was cooled to 0 °C. NIS (34 mg, 150 μmol, 1.5 eq.) and TfOH (1.3 μL, 0.014 mmol, 0.15 eq.) was added and the temperature was raised to r.t.. The reaction was put overnight but some starting material remained. The reaction was quenched with $NaHCO_3$ (aq. sat.), extracted with EtOAc, washed with water and brine, dried with Na_2SO_4(s) and concentrated *in vacuo*. The product was further purified with flash chromatography to get crude intermediate, 76 mg as light-yellow foam. The foam was coevaporated with building block **5** (50 mg, 101 μmol, 1.2 eq.) with toluene and put under vacuum overnight to remove the trace water. After dissolved in a mixed solvent (1 mL, 1,4-dioxane:toluene = 4:1) and 4 Å MS were added, the mixture was cooled to 0 °C using ice-water bath. NIS (38 mg, 168 μmol, 1.5 eq.) and TfOH (2.0 μL, 17 μmol, 0.2 eq.) were then added sequentially to the mixture and the mixture was performed at that temperature until the TLC showed full conversion of the starting material. The reaction was quenched with $Na_2S_2O_3$(aq.), extracted with EtOAc, washed with water and brine, dried with Na_2SO_4(s) and concentrated *in vacuo*. The product was further purified with flash chromatography to get crude

syrup product, 72 mg. The syrup was dissolved in a mix of DCM and TEA (1 mL, 4:1) and after stirring for 1 h, the resultant mixture was concentrated and purified by flash chromatography to get colorless syrup disaccharide **11**, 40. mg, 36 μmol, 50% for 3 steps. ^{1}H NMR (400 MHz, Chloroform-*d*) δ 7.43 – 7.09 (m, 36H), 5.59 (dd, *J* = 3.3, 1.4 Hz, 1H), 5.18 (s, 2H), 4.99 (t, *J* = 25.2 Hz, 2H), 4.85 (d, *J* = 11.7 Hz, 1H), 4.78 (d, *J* = 10.8 Hz, 1H), 4.70 (d, *J* = 17.0 Hz, 2H), 4.66 – 4.59 (m, 4H), 4.53 (d, *J* = 10.8 Hz, 1H), 4.48 (s, 1H), 4.27 (d, *J* = 11.7 Hz, 2H), 4.20 – 4.13 (m, 1H), 4.11 – 3.97 (m, 3H), 3.94 (dd, *J* = 10.2, 3.3 Hz, 2H), 3.88 (s, 1H), 3.83 (dd, *J* = 10.1, 3.5 Hz, 3H), 3.79 – 3.72 (m, 2H), 3.72 – 3.44 (m, 4H), 2.85 – 2.51 (m, 1H), 2.14 (s, 3H), 2.08 (s, 3H), 1.36 (s, 2H), 1.31 (s, 4H), 1.28 (s, 12H). ^{13}C NMR (101 MHz, $CDCl_3$) δ 170.7, 170.4, 138.3, 137.9, 128.7, 128.5, 128.5, 128.4, 128.1, 128.0, 128.0, 127.7, 127.6, 127.3, 124.5, 100.3, 97.3, 77.4, 77.0, 76.7, 76.1, 75.4, 74.3, 72.8, 71.8, 69.6, 68.5, 68.1, 67.9, 67.5, 67.3, 62.5, 51.2, 32.0, 31.5, 30.2, 29.7, 29.4, 22.7, 20.9, 20.9, 14.2, 1.1. HRMS (Q-Tof): calculated for $C_{62}H_{67}F_2NO_{15}Na^+$ [M+Na]$^+$ 1126.4371, found 1126.4319.

2,3,5,6-Tetra-*O*-benzoyl-*β*-D-galatofuranosyl-(1→3)-4-*O*-[4,6-di-*O*-acetyl-2,3-di-*O*-benzyl-*α*-D-galactopyranosyl-(1→)]-2,6-di-*O*-benzyl-*α*-D-galactopyranosyl-(1→1)-(3-*N*-benzyl-*N*-benzyloxycarbonylamino)-2,2-difluoropropanol (11)

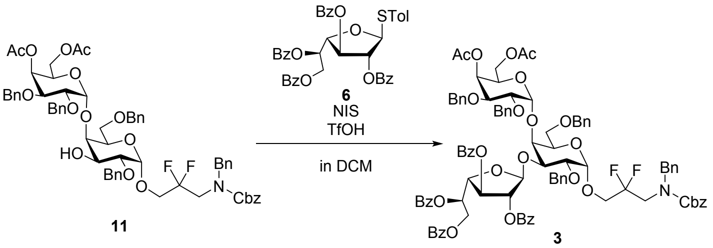

The donor **6** (22 mg, 31 μmol, 1.2 eq.) and the acceptor **11** (28 mg, 26 μmol, 1.0 eq.) were dissolved in DCM and the solution was cooled to -30 °C. After 5 min, NIS (9 mg, 38 μmol, 1.5 eq.) and TfOH (0.2 μL, 2.6 μmol, 0.1 eq.) were added sequentially to the mixture and the solution was allowed to stirr at low temperature for 3 h. (-30 °C ~ -10 °C) Most of the starting material had been cconsumed (TLC) and the reaction was quenched with $Na_2S_2O_3$(aq., sat.). After filtration, extraction and wash with water and brine, the mixture was dried with Na_2SO_4(s), concentrated *in vacuo* and further purified with flash chromatography to get white solid like syrup trisaccharide **3**. 30 mg, 18 μmol, yield: 69%. ^{1}H NMR (400 MHz, Chloroform-*d*) δ 8.04 (d, *J* = 7.7 Hz, 4H), 8.00 – 7.93 (m, 2H), 7.78 (d, *J* = 7.8 Hz, 2H), 7.57 – 7.48 (m, 3H), 7.51 – 7.42 (m, 3H), 7.41 (t, *J* = 7.7 Hz, 3H), 7.38 – 7.00 (m, 38H), 6.08 (dt, *J* = 7.2, 3.7 Hz, 1H), 5.82 (d, *J* = 10.2 Hz, 1H), 5.66 (s, 1H), 5.64 – 5.56 (m, 2H), 5.15 (s, 2H), 5.03 (d, *J* = 3.2 Hz, 1H), 4.94 (d, *J* = 36.6 Hz, 1H), 4.76 – 4.40 (m, 11H), 4.36 – 4.21 (m, 4H), 4.24 – 4.06 (m, 2H), 4.04 – 3.87 (m, 4H), 3.78 (dd, *J* = 25.2, 12.1 Hz, 2H), 3.73 – 3.46 (m, 5H), 2.14 (s, 3H), 2.01 (s, 3H), 0.87 (dt, *J* = 12.9, 7.1 Hz, 1H). ^{13}C NMR (101 MHz, $CDCl_3$) δ 170.5, 170.3, 166.1, 165.7, 165.6, 165.1, 138.4, 138.1, 137.0, 133.4, 133.3, 133.1, 130.1, 130.0, 129.8, 129.7, 129.5, 129.4, 128.9, 128.6, 128.5, 128.5, 128.4, 128.4, 128.3, 128.3, 128.1, 128.0, 128.0, 127.8, 127.7, 127.5, 127.4, 107.1, 99.8, 97.4, 81.7, 79.3, 78.1, 77.4, 77.0, 76.7, 75.9, 74.1, 73.4, 72.9, 71.9, 70.2, 67.8, 67.2, 67.1, 63.7, 61.4, 31.0, 29.7, 21.1, 20.8, 1.1. HRMS (Q-Tof): calculated for $C_{96}H_{93}F_2NO_{24}Na^+$ [M+Na]$^+$ 1704.5948, found 1705.5908.

2,5,6-Tri-*O*-benzoyl-3-*tert*-butyldimethylsilyl-*β*-D-galatofuranosyl-(1→3)-4-*O*-[4,6-di-*O*-acetyl-2,3-di-*O*-benzyl-*α*-D-galactopyranosyl-(1→)]-2,6-di-*O*-benzyl-*α*-D-galactopyranosyl-(1→1)-(3-*N*-benzyl-*N*-benzyloxycarbonylamino)-2,2-difluoropropanol (18)

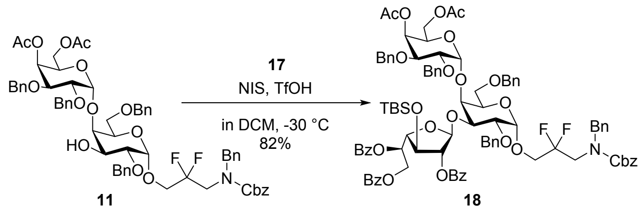

Disaccharide **11** (40 mg, 36 μmol, 1.0 eq.) and donor **17** (38 mg, 54 μmol, 1.5 eq.) were coevaporated with toluene and put under high vacuum to remove any trace water. After being dissolved in DCM, dried with 4 Å MS and cooled to -30 °C, NIS (16 mg, 72 μmol, 2.0 eq.) was added to the mixture. After addition of TfOH (1.0 μL, 4 μmol, 0.2 eq.), the mixture was stirred for 30 min. The reaction was quenched with $Na_2S_2O_3$(aq., sat.). After filtration, extraction and wash with water and brine, the mixture was dried with Na_2SO_4(s), concentrated *in vacuo* and further purified with flash chromatography to get trisaccharide **18**, 50 mg, 30 μmol, yield: 82%. ^{1}H NMR (400 MHz, Chloroform-*d*) δ 8.09 (d, *J* = 7.6 Hz, 2H), 8.00 – 7.94 (m, 2H), 7.83 (d, *J* = 7.7 Hz, 2H), 7.64 – 7.45 (m, 3H), 7.45 – 7.14 (m, 33H), 7.10 (d, *J* = 5.6 Hz, 5H), 5.78 (dt, *J* = 6.3, 3.2 Hz, 1H), 5.61 (dd, *J* = 3.4, 1.4 Hz, 1H), 5.56 (d, *J* = 10.3 Hz, 1H), 5.46 (d, *J* = 3.9 Hz, 1H), 5.15 (d, *J* = 2.6 Hz, 2H), 5.10 (d, *J* = 3.3 Hz, 1H), 4.98 – 4.83 (m, 1H), 4.77 (dd, *J* = 11.3, 5.0 Hz, 2H), 4.72 – 4.42 (m, 10H), 4.38 (dp, *J* = 9.1, 5.3 Hz, 2H), 4.24 – 4.18 (m, 2H), 4.18 – 4.07 (m, 2H), 4.02 – 3.46 (m, 11H), 2.13 (s, 3H), 2.10 (s, 3H), 0.85 (s, 9H). ^{13}C NMR (101 MHz, $CDCl_3$) δ 170.6, 170.5, 166.1, 166.0, 165.1, 156.8, 138.7, 138.5, 138.3, 138.2, 137.1, 136.5, 136.3, 133.5, 133.4, 133.2, 130.0, 129.8, 129.8, 129.7, 129.6, 129.3, 128.6, 128.6, 128.5, 128.5, 128.4, 128.4, 128.3, 128.2, 128.1, 128.0, 127.9, 127.8, 127.7, 127.6, 127.5, 127.4, 121.6, 114.2, 107.7, 99.9, 97.8, 84.2, 82.3, 82.2, 79.6, 77.5, 77.2, 76.8, 76.5, 76.3, 75.6, 74.8, 74.3, 73.1, 72.9, 71.8, 70.8, 70.7, 69.5, 69.3, 67.9, 67.2, 67.1, 66.4, 63.8, 61.2, 60.6, 51.3, 51.1, 47.8, 46.9, 34.0, 32.1, 31.6, 30.3, 29.8, 29.8, 29.8, 29.6, 29.5, 29.3, 29.1, 25.8, 22.8, 21.2, 21.1, 21.0, 18.0, 14.3, 14.3, 13.9, -4.7, -5.3. HRMS (Q-Tof): calculated for $C_{95}H_{103}F_2NO_{23}SiNa^+$ [M+Na]$^+$ 1714.6550, found 1714.6553.

2,5,6-Tri-*O*-benzoyl-*β*-D-galatofuranosyl-(1→3)-4-*O*-[4,6-di-*O*-acetyl-2,3-di-*O*-benzyl-*α*-D-galactopyranosyl-(1→)]-2,6-di-*O*-benzyl-*α*-D-galactopyranosyl-(1→1)-(3-*N*-benzyl-*N*-benzyloxycarbonylamino)-2,2-difluoropropanol (10)

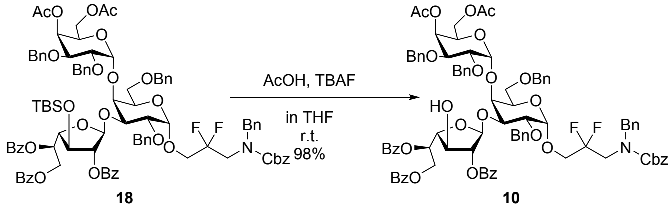

TBS protected trisaccharide **18** (13 mg, 7.7 μmol, 1.0 eq.) was dissolved in THF (0.5 mL). AcOH (5 μL, 77 μmol, 10 eq.) and TBAF (1 M in THF, 77 μL, 77 μmol, 10 eq.) were added to the solution and the mixture was allowed stirr at ambient temperature until TLC showed full conversion of the starting material. The resultant was diluted with, EtOAc (20 mL), washed with

$NaHCO_3$ (sat. aq.) and brine, dried with Na_2SO_4(s), concentrated *in vacuo* and purified with flash chromatography (30% EtOAc in Hexane) to get 12 mg white syrup as trisaccharide **10**, 7.6 μmol, yield: 98%. ^{1}H NMR (600 MHz, Chloroform-*d*) δ 8.02 (d, *J* = 7.6 Hz, 2H), 7.98 – 7.93 (m, 2H), 7.88 (d, *J* = 7.7 Hz, 2H), 7.54 (t, *J* = 7.6 Hz, 1H), 7.48 (td, *J* = 7.7, 3.5 Hz, 2H), 7.39 – 7.26 (m, 21H), 7.26 – 7.06 (m, 15H), 5.81 (dt, *J* = 8.4, 4.5 Hz, 1H), 5.68 (d, *J* = 22.5 Hz, 1H), 5.60 (dd, *J* = 3.4, 1.5 Hz, 1H), 5.24 (d, *J* = 2.3 Hz, 1H), 5.15 (s, 2H), 5.02 – 4.88 (m, 3H), 4.77 (dd, *J* = 21.1, 11.2 Hz, 2H), 4.71 (dd, *J* = 11.9, 4.1 Hz, 1H), 4.69 – 4.61 (m, 4H), 4.58 (d, *J* = 11.5 Hz, 1H), 4.53 (d, *J* = 11.6 Hz, 1H), 4.51 – 4.46 (m, 2H), 4.44 (t, *J* = 7.0 Hz, 1H), 4.28 – 4.22 (m, 3H), 4.14 (dt, *J* = 20.9, 7.6 Hz, 3H), 4.07 (t, *J* = 6.7 Hz, 1H), 3.95 (dd, *J* = 10.2, 3.5 Hz, 3H), 3.88 – 3.81 (m, 2H), 3.78 (dd, *J* = 10.2, 3.3 Hz, 2H), 3.74 – 3.58 (m, 4H), 3.55 (t, *J* = 8.8 Hz, 1H), 2.11 (s, 3H), 2.10 (s, 3H), 0.94 (t, *J* = 7.4 Hz, 1H), 0.91 – 0.82 (m, 5H). ^{13}C NMR (151 MHz, cdcl$_3$) δ 171.4, 170.8, 170.5, 166.3, 166.2, 166.0, 156.8, 139.4, 138.5, 138.5, 138.2, 137.1, 133.6, 133.4, 133.2, 130.0, 130.0, 129.9, 129.8, 129.7, 129.0, 128.7, 128.6, 128.6, 128.5, 128.5, 128.5, 128.4, 128.2, 128.2, 128.1, 127.9, 127.9, 127.8, 127.8, 127.6, 127.4, 124.6, 114.2, 107.2, 100.8, 97.4, 84.7, 83.5, 80.4, 77.4, 77.2, 76.9, 76.2, 76.1, 75.9, 74.6, 74.4, 72.9, 72.8, 72.6, 72.0, 70.7, 70.5, 69.1, 67.9, 67.6, 67.4, 64.5, 63.5, 61.8, 51.3, 37.3, 34.0, 32.1, 31.6, 30.8, 30.3, 29.8, 29.8, 29.8, 29.7, 29.5, 29.4, 29.3, 29.1, 22.8, 21.2, 21.1, 21.0, 19.3, 14.3, 13.9. HRMS (Q-Tof): calculated for $C_{89}H_{89}F_2NO_{23}Na^+$ $[M+Na]^+$ 1600.5686, found 1600.5693.

Phenyl 4,6-di-*O*-acetyl-2,3-di-*O*-benzyl-*α*-D-galactopyranosyl-(1→4)-2,6-di-*O*-benzyl-3-*O*-fluorenylmethoxycarbonyl-1-thio-*α*-D-galactopyranoside (20)

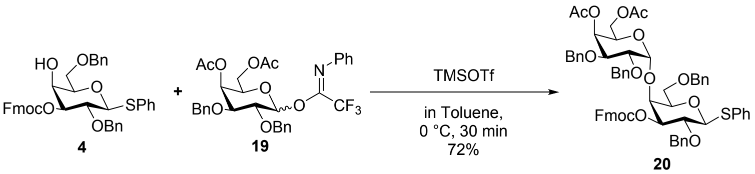

Acceptor **4** (64 mg, 95 μmol, 1.0 eq.) and donor **19** (70 mg, 114 μmol, 1.2 eq.) were evaporated with toluene (5 mL x 3) and put into high vacuum to remove trace water before they were dissolved in anhydrous toluene (2 mL). 4 Å MS were added to the solution and the mixture was cooled to 0 °C using an ice-water bath. TMSOTf (2 μL, 11 μmol, 0.1 eq.) was added to the mixture and the reaction was put at 0 °C until TLC showed the disappearance of the starting material. The reaction was quenched with $Na_2S_2O_3$(aq.), washed with brine, dried with Na_2SO_4(s), concentrated *in vacuo* and purified with flash chromatography to get disaccharide **20**, 158 mg, 144 μmol, 72%. ^{1}H NMR (400 MHz, Chloroform-*d*) δ 7.75 (ddt, *J* = 7.6, 1.7, 0.8 Hz, 2H), 7.71 – 7.66 (m, 2H), 7.59 (dd, *J* = 7.5, 1.0 Hz, 1H), 7.54 (dt, *J* = 7.6, 1.0 Hz, 1H), 7.43 – 7.27 (m, 19H), 7.25 – 7.14 (m, 11H), 5.60 (dd, *J* = 3.2, 1.4 Hz, 1H), 4.96 (d, *J* = 3.7 Hz, 1H), 4.93 (d, *J* = 11.6 Hz, 1H), 4.85 (d, *J* = 10.7 Hz, 1H), 4.83 – 4.79 (m, 1H), 4.76 (d, *J* = 10.6 Hz, 1H), 4.66 (d, *J* = 6.2 Hz, 1H), 4.64 – 4.59 (m, 2H), 4.53 (d, *J* = 10.6 Hz, 1H), 4.44 – 4.40 (m, 2H), 4.31 (ddd, *J* = 7.8, 6.3, 1.5 Hz, 1H), 4.26 (s, 2H), 4.24 (d, *J* = 3.0 Hz, 1H), 4.18 (d, *J* = 7.0 Hz, 1H), 4.06 (d, *J* = 7.7 Hz, 2H), 3.97 – 3.92 (m, 1H), 3.92 – 3.88 (m, 1H), 3.88 – 3.81 (m, 2H), 3.74 (dd, *J* = 7.7, 6.0 Hz, 1H), 3.62 (dd, *J* = 9.4, 5.9 Hz, 1H), 2.12 (s, 3H), 1.90 (s, 3H). ^{13}C NMR (101 MHz, $CDCl_3$) δ 170.5, 170.5, 154.7, 143.6, 143.0, 141.4, 141.4, 138.5, 138.1, 137.9, 137.6, 133.4, 132.0, 129.1, 128.6, 128.6, 128.5, 128.5, 128.4, 128.2, 128.1, 128.1, 128.0, 128.0, 127.9, 127.9, 127.8, 127.8, 127.3, 127.3, 125.2, 125.1, 120.2, 100.0, 86.6, 79.9, 77.5, 77.4, 77.2, 77.1, 76.8, 76.6, 75.6, 75.1, 74.8, 74.7, 73.7, 73.2, 71.7, 70.3, 67.3, 66.9, 66.7, 61.2, 46.7, 21.1, 20.9. HRMS (Q-Tof): calculated for $C_{65}H_{68}NO_{14}S^+$ $[M+NH_4]^+$ 1118.4355, found 1118.4377 m/z.

Phenyl 4,6-di-*O*-acetyl-2,3-di-*O*-benzyl-*α*-D-galactopyranosyl-(1→4)-2,6-di-*O*-benzyl-1-thio-*α*-D-galactopyranoside (21)

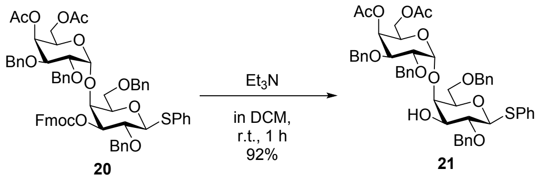

Fmoc protected disaccharide **20** (30 mg, 27 μmol, 1.0 eq.) was dissolved in DCM (2 mL) and Et_3N (1 mL) was added to the solution. The mixture was stirred at r.t. until TLC showed full conversion of the starting material. The solvent was removed *in vacuo* and the crude product was purified with flash chromatography to get 22 mg product **21** (25 μmol), yield: 92%. ^1H NMR (600 MHz, Chloroform-*d*) δ 7.67 – 7.62 (m, 2H), 7.42 – 7.39 (m, 2H), 7.37 (t, *J* = 7.4 Hz, 2H), 7.34 – 7.26 (m, 14H), 7.24 – 7.20 (m, 5H), 5.48 (dd, *J* = 3.3, 1.4 Hz, 1H), 5.00 (d, *J* = 3.6 Hz, 1H), 4.87 (d, *J* = 11.7 Hz, 1H), 4.82 (t, *J* = 11.3 Hz, 2H), 4.72 (d, *J* = 10.9 Hz, 1H), 4.65 (d, *J* = 11.7 Hz, 1H), 4.59 (dd, *J* = 12.2, 10.1 Hz, 2H), 4.28 (d, *J* = 3.0 Hz, 2H), 4.08 (ddd, *J* = 7.0, 5.3, 1.5 Hz, 1H), 4.04 – 4.00 (m, 2H), 3.96 (dd, *J* = 11.3, 7.5 Hz, 1H), 3.91 (dd, *J* = 10.1, 3.2 Hz, 1H), 3.87 (dd, *J* = 9.6, 6.5 Hz, 1H), 3.82 (dd, *J* = 10.1, 3.6 Hz, 1H), 3.71 (t, *J* = 6.2 Hz, 1H), 3.67 (td, *J* = 9.9, 4.4 Hz, 2H), 3.49 (t, *J* = 9.4 Hz, 1H), 2.12 (s, 3H), 2.01 (s, 3H). ^{13}C NMR (101 MHz, $CDCl_3$) δ 170.6, 170.4, 138.5, 138.1, 138.1, 138.0, 133.4, 132.5, 128.9, 128.7, 128.6, 128.5, 128.5, 128.4, 128.2, 128.1, 128.0, 128.0, 127.9, 127.9, 127.8, 127.7, 127.4, 100.4, 86.3, 79.1, 77.6, 77.5, 77.4, 77.2, 77.0, 76.8, 76.0, 75.5, 74.8, 74.5, 74.4, 73.2, 71.9, 68.5, 67.5, 67.4, 62.5, 29.8, 21.0, 21.0. HRMS (Q-Tof): calculated for $C_{50}H_{58}NO_{12}S^+$ $[M+NH_4]^+$ 896.3674, found 896.3688 m/z.

Phenyl 2,3,5,6-tetra-*O*-benzoyl-*β*-D-galatofuranosyl-(1→3)-4-*O*-[4,6-di-*O*-acetyl-2,3-di-*O*-benzyl-*α*-D-galactopyranosyl-(1→)]-2,6-di-*O*-benzyl-1-thio-*β*-D-galactopyranoside (9)

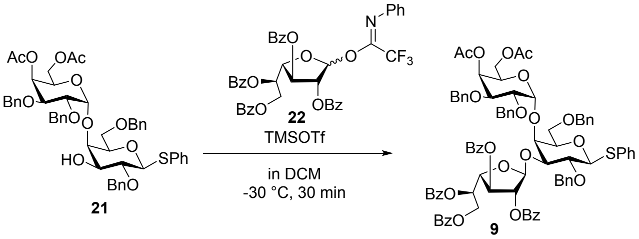

Acceptor **21** (32 mg, 36 μmol, 1.0 eq.) and Gal*f* donor **22** (41 mg, 54 μmol, 1.5 eq.) were evaporated with toluene (5 mL x 3) and put into high vacuum to remove trace water before they were dissolved in anhydrous DCM (1 mL). 4 Å MS were added to the solution and the mixture was cooled to -30 °C with dry ice-acetone bath. TMSOTf (2 μL, 8 μmol, 0.2 eq.) was added to the mixture and the reaction was put at -30 °C until TLC showed the disappearance of the starting material. The reaction was quenched with $Na_2S_2O_3$(aq.), extracted with EtOAc, washed with brine, dried with Na_2SO_4(s), concentrated *in vacuo* and purified with flash chromatography to get trisaccharide **9**, 40 mg, 27 μmol, 75%. ^1H NMR (600 MHz, Chloroform-*d*) δ 8.09 (d, *J* = 7.6 Hz, 2H), 8.04 – 8.00 (m, 6H), 7.98 (d, *J* = 7.8 Hz, 4H), 7.88 (d, *J* = 7.7 Hz, 2H), 7.78 (d, *J* = 7.7 Hz, 2H), 7.62 (dd, *J* = 7.4, 2.2 Hz, 2H), 7.50 (dddd, *J* = 21.4, 10.9, 8.4, 5.9 Hz, 10H), 7.40 (dd, *J* = 11.5, 7.5 Hz, 5H), 7.36 – 7.27 (m, 22H), 7.20 (d, *J* = 6.5 Hz, 4H), 7.18 – 7.16 (m, 2H), 7.07 (dd, *J* = 8.5, 3.0 Hz, 2H), 6.09 (dt, *J* = 7.4, 3.9 Hz, 1H), 6.04 (dt, *J* = 6.8, 3.7 Hz, 1H), 5.10 (d, *J* = 3.3

Hz, 1H), 4.92 (dd, J = 5.1, 3.7 Hz, 1H), 4.84 (d, J = 10.3 Hz, 1H), 4.79 (td, J = 13.2, 12.6, 2.8 Hz, 3H), 4.76 – 4.66 (m, 6H), 4.59 – 4.55 (m, 2H), 4.51 (d, J = 11.1 Hz, 1H), 4.34 (d, J = 17.2 Hz, 3H), 4.25 (d, J = 7.3 Hz, 2H), 4.08 – 4.03 (m, 1H), 3.97 – 3.91 (m, 2H), 3.87 (dd, J = 9.6, 2.5 Hz, 1H), 3.80 (d, J = 9.4 Hz, 1H), 3.79 – 3.75 (m, 1H), 3.69 (ddd, J = 15.6, 9.8, 6.5 Hz, 2H), 3.59 (dd, J = 10.0, 3.3 Hz, 1H), 2.15 (s, 3H), 1.99 (s, 3H). ^{13}C NMR (151 MHz, cdcl$_3$) δ 170.4, 170.4, 166.3, 166.2, 165.9, 165.8, 165.7, 165.7, 165.3, 138.6, 138.4, 138.3, 138.0, 133.7, 133.6, 133.5, 133.5, 133.4, 133.4, 133.3, 133.1, 132.3, 130.2, 130.2, 130.1, 130.1, 130.1, 130.0, 130.0, 130.0, 129.9, 129.9, 129.9, 129.9, 129.7, 129.7, 129.6, 129.5, 129.0, 128.9, 128.9, 128.6, 128.6, 128.6, 128.5, 128.5, 128.5, 128.4, 128.3, 128.2, 128.0, 127.9, 127.8, 127.8, 127.7, 127.7, 127.4, 107.2, 101.2, 99.8, 87.1, 82.6, 82.3, 82.1, 82.0, 79.0, 78.6, 78.5, 78.3, 77.6, 77.5, 77.4, 77.2, 76.9, 76.8, 76.0, 75.1, 74.3, 73.2, 71.9, 70.5, 70.4, 69.7, 67.5, 67.3, 63.9, 63.7, 61.6, 22.8, 21.2, 20.9, 14.3. HRMS (Q-Tof): calculated for $C_{84}H_{80}O_{21}SNa^+$ [M+Na]$^+$ 1479.4805, found 1479.4836.

2,3,5,6-Tetra-*O*-benzoyl-*β*-D-galatofuranosyl-(1→3)-4-*O*-[4,6-di-*O*-acetyl-2,3-di-*O*-benzyl-*α*-D-galactopyranosyl-(1→)]-2,6-di-*O*-benzyl-*α/β*-D-galactopyranoside (23)

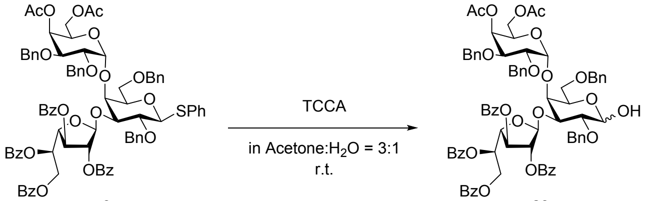

Thiolglycoside **9** (31 mg, 21 μmol, 1.0 eq.) was dissolved in a mixture of acetone and water (v/v = 3/1, 2 mL) before TCCA (6 mg, 26 μmol, 1.2 eq.) was added. The mixture was stirred at r.t. for 1 h but some of the starting material was not converting. The reaction was quenched with $NaHCO_3$ (sat. aq.), washed with NaCl(aq.), dried with Na_2SO_4(s), concentrated in vacuo and further purified with flash chromatography to get white syrup **23** (α:β = 2:1, 21 mg, 15 μmol), yield: 72%. Starting material **9** was recovered with an amount of 5 mg. ^{1}H NMR (600 MHz, Chloroform-*d*) δ 8.09 – 7.95 (m, 6H), 7.79 (ddd, J = 14.7, 8.3, 1.4 Hz, 2H), 7.57 – 7.45 (m, 4H), 7.42 (dt, J = 9.4, 7.7 Hz, 2H), 7.37 – 7.26 (m, 12H), 7.25 – 7.20 (m, 4H), 7.16 (ddd, J = 9.6, 5.2, 2.7 Hz, 5H), 7.13 – 7.03 (m, 3H), 6.10 – 6.04 (m, 1H), 5.79 (d, J = 11.0 Hz, 1H), 5.67 – 5.54 (m, 3H), 5.29 (d, J = 3.4 Hz, 1H), 5.10 (dd, J = 55.7, 3.3 Hz, 1H), 5.02 – 4.89 (m, 1H), 4.79 – 4.70 (m, 4H), 4.69 – 4.65 (m, 1H), 4.62 – 4.55 (m, 2H), 4.54 – 4.42 (m, 3H), 4.35 – 4.27 (m, 3H), 4.27 – 4.21 (m, 2H), 4.21 – 4.15 (m, 1H), 4.15 – 4.11 (m, 1H), 4.07 (t, J = 6.7 Hz, 1H), 3.97 – 3.86 (m, 3H), 3.82 – 3.63 (m, 3H), 3.61 – 3.51 (m, 2H), 2.15 (d, J = 16.5 Hz, 3H), 2.05 (s, 1H), 2.03 (s, 1H), 2.01 (s, 2H). ^{13}C NMR (151 MHz, cdcl$_3$) δ 170.9, 170.5, 170.4, 170.4, 166.3, 166.3, 165.8, 165.8, 165.8, 165.3, 147.2, 139.4, 138.5, 138.5, 138.3, 138.3, 138.2, 138.2, 137.9, 133.5, 133.5, 133.4, 133.4, 133.4, 133.3, 133.2, 130.3, 130.2, 130.1, 130.1, 130.0, 129.9, 129.9, 129.9, 129.7, 129.6, 129.5, 129.0, 129.0, 129.0, 128.6, 128.6, 128.6, 128.5, 128.5, 128.4, 128.4, 128.3, 128.2, 128.1, 128.1, 128.0, 127.9, 127.9, 127.8, 127.8, 127.8, 127.7, 124.6, 124.1, 114.2, 107.3, 107.2, 99.9, 99.4, 98.1, 91.5, 82.6, 82.0, 81.9, 81.9, 80.9, 79.5, 78.4, 78.2, 77.4, 77.2, 76.9, 76.8, 76.1, 74.8, 74.7, 74.1, 74.1, 73.9, 73.5, 73.3, 73.2, 72.1, 72.0, 70.6, 70.4, 70.4, 70.2, 69.8, 67.4, 67.4, 67.2, 67.1, 64.5, 63.8, 63.8, 61.8, 61.5, 37.2, 35.0, 34.7, 34.0, 32.1, 31.6, 30.8, 30.5, 30.3, 30.2, 29.8, 29.8, 29.8, 29.7, 29.5, 29.3, 29.2, 29.1, 27.2, 22.8, 22.8, 21.6, 21.2, 21.2, 21.0, 20.9,

19.3, 14.3, 13.9, 1.2. HRMS (Q-Tof): calculated for $C_{78}H_{76}O_{22}Na^+$ $[M+Na]^+$ 1387.4720, found 1387.4723.

2,3,5,6-Tetra-*O*-benzoyl-*β*-d-galatofuranosyl-(1→3)-4-*O*-[4,6-di-*O*-acetyl-2,3-di-*O*-benzyl-*α*-d-galactopyranosyl-(1→)]-2,6-di-*O*-benzyl-*α/β*-D-galactopyranosyl-*N*-phenyltrifluoroacetimidate (24)

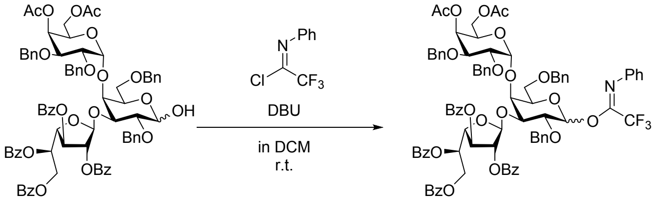

23 **24**

Hydrolyzed trisaccharide **23** (20 mg, 15 μmol, 1.0 eq.) and trifluoro-*N*-phenylacetimidoyl chloride (15 μL, 90 μmol, 6 eq.) were dissolved in anhydrous DCM (2 mL). DBU (8 μL, 45 μmol, 3.0 eq.) was added and the resultant mixture was stirred at ambient temperature until TLC indicated disappearance of the starting material. The crude product was concentrated *in vacuo* and purified by flash chromatography to get white syrup as donor **24** (α:β = 1:4, 21 mg, 14 μmol), yield: 93%. ^{1}H NMR (400 MHz, Chloroform-*d*) δ 8.09 – 7.96 (m, 6H), 7.78 (ddd, J = 12.4, 8.3, 1.4 Hz, 2H), 7.61 – 7.27 (m, 18H), 7.25 – 7.04 (m, 18H), 6.77 (d, J = 7.8 Hz, 1H), 6.08 (ddt, J = 25.6, 6.9, 3.3 Hz, 1H), 5.87 – 5.73 (m, 1H), 5.68 – 5.58 (m, 3H), 5.09 (dd, J = 26.5, 3.3 Hz, 1H), 4.85 – 4.60 (m, 6H), 4.59 – 4.40 (m, 3H), 4.36 – 4.24 (m, 4H), 4.23 – 4.05 (m, 2H), 4.05 – 3.52 (m, 8H), 2.15 (d, J = 3.7 Hz, 3H), 2.03 (d, J = 5.2 Hz, 3H). ^{13}C NMR (101 MHz, $CDCl_3$) δ 170.6, 170.4, 166.4, 166.3, 165.9, 165.8, 165.8, 165.3, 165.3, 147.2, 143.5, 139.4, 138.5, 138.4, 138.3, 138.2, 138.2, 138.1, 138.1, 138.1, 137.8, 137.6, 137.6, 135.2, 133.6, 133.5, 133.4, 133.3, 130.3, 130.2, 130.1, 130.1, 130.0, 129.9, 129.8, 129.7, 129.6, 129.5, 129.5, 129.4, 129.0, 128.9, 128.9, 128.8, 128.7, 128.7, 128.7, 128.6, 128.6, 128.5, 128.5, 128.5, 128.5, 128.4, 128.4, 128.3, 128.2, 128.2, 128.1, 128.1, 128.1, 128.0, 127.9, 127.9, 127.8, 127.8, 127.8, 127.7, 127.7, 127.7, 127.6, 126.5, 124.6, 124.4, 124.1, 120.5, 120.4, 119.4, 114.2, 107.2, 107.2, 99.9, 91.0, 82.8, 82.4, 82.1, 81.9, 81.8, 78.2, 77.5, 77.2, 76.8, 75.9, 75.8, 75.2, 74.3, 74.2, 74.1, 74.0, 73.7, 73.4, 73.4, 73.3, 73.2, 73.1, 72.1, 72.0, 71.9, 70.5, 70.3, 69.7, 67.6, 67.5, 67.5, 67.2, 63.8, 63.7, 61.9, 61.8, 61.4, 45.4, 37.2, 35.0, 34.0, 33.8, 32.9, 32.1, 31.6, 30.3, 30.2, 29.8, 29.8, 29.8, 29.7, 29.5, 29.3, 29.1, 28.1, 27.7, 27.6, 27.2, 22.8, 22.8, 21.6, 21.2, 20.9, 20.9, 19.9, 14.3, 1.2. MS not available due to instability of the imidate.

2,3,5,6-Tetra-*O*-benzoyl-*β*-D-galatofuranosyl-(1→3)-4-*O*-[4,6-di-*O*-acetyl-2,3-di-*O*-benzyl-*α*-D-galactopyranosyl-(1→)]-2,6-di-*O*-benzyl-*α*-D-galactopyranosyl-(1→3)-2,5,6-Tri-*O*-benzoyl-*β*-D-galatofuranosyl-(1→3)-4-*O*-[4,6-di-*O*-acetyl-2,3-di-*O*-benzyl-*α*-D-galactopyranosyl-(1→)]-2,6-di-*O*-benzyl-*α*-D-galactopyranosyl-(1→1)-(3-*N*-benzyl-*N*-benzyloxycarbonylamino)-2,2-difluoropropanol (8)

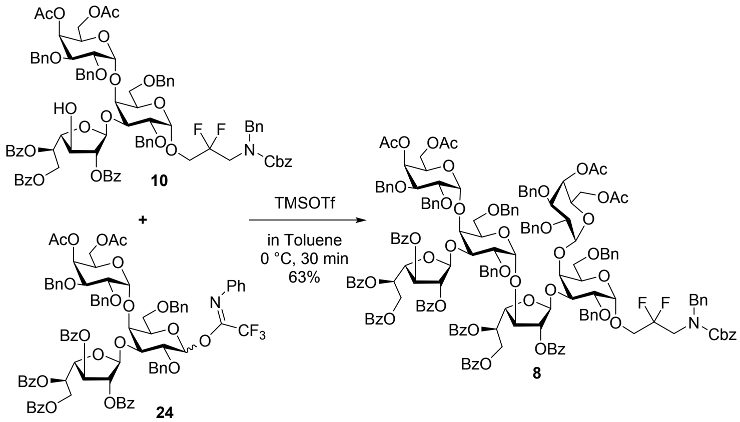

Donor **24** (5 mg, 3.2 μmol, 1.0 eq.) and acceptor **10** (9 mg, 5.7 μmol, 1.7 eq.) were coevaporated with toluene and put under high vacuum to remove trace water before they were dissolved in anhydrous toluene (0.5 mL). Molecular sieves (4 Å) were added and the mixture was cooled to 0 °C with ice water bath. TMSOTf (0.1 μL, 0.2 μmol, 0.2 eq.) was added and after TLC showed full conversion of the donor, the reaction was quenched by triethylamine(l), diluted with EtOAc, washed with NaCl (sat. aq.), dried with Na_2SO_4(s), concentrated *in vacuo* and further purified with flash chromatography to get colorless syrup as protected hexasaccharide **8** (6 mg, 2 μmol), yield: 63%. ^{1}H NMR (700 MHz, Chloroform-*d*) δ 8.12 (d, *J* = 8.6 Hz, 2H), 8.01 (d, *J* = 7.8 Hz, 2H), 7.97 (d, *J* = 7.7 Hz, 4H), 7.86 (t, *J* = 7.9 Hz, 4H), 7.74 (d, *J* = 7.8 Hz, 2H), 7.58 (t, *J* = 7.5 Hz, 1H), 7.44 (ddq, *J* = 26.6, 14.0, 7.8, 4.9 Hz, 9H), 7.35 – 7.27 (m, 17H), 7.19 (dtd, *J* = 33.8, 17.7, 16.0, 8.4 Hz, 29H), 7.11 – 7.05 (m, 6H), 7.01 (t, *J* = 7.5 Hz, 2H), 6.97 (t, *J* = 9.4 Hz, 4H), 6.92 (dd, *J* = 10.4, 6.9 Hz, 3H), 6.04 (dd, *J* = 7.6, 3.5 Hz, 1H), 5.88 (s, 1H), 5.76 (dd, *J* = 14.6, 8.5 Hz, 2H), 5.70 – 5.65 (m, 2H), 5.62 (d, *J* = 6.2 Hz, 1H), 5.56 – 5.46 (m, 2H), 5.13 (d, *J* = 11.0 Hz, 3H), 4.96 (t, *J* = 3.9 Hz, 2H), 4.92 – 4.86 (m, 1H), 4.83 – 4.72 (m, 4H), 4.69 – 4.58 (m, 8H), 4.58 – 4.31 (m, 13H), 4.30 – 4.24 (m, 2H), 4.20 – 4.02 (m, 6H), 4.01 – 3.95 (m, 2H), 3.94 – 3.79 (m, 5H), 3.78 – 3.57 (m, 8H), 3.52 (dd, *J* = 10.1, 3.1 Hz, 1H), 3.49 (t, *J* = 8.3 Hz, 1H), 3.08 (dd, *J* = 9.9, 6.0 Hz, 1H), 2.36 (s, 5H), 2.07 (s, 3H), 2.00 (s, 3H), 1.96 (s, 3H), 1.90 (s, 3H), 0.90 – 0.81 (m, 3H). ^{13}C NMR (176 MHz, $CDCl_3$) δ 207.4, 185.8, 170.6, 170.5, 170.5, 170.4, 166.2, 166.1, 165.9, 165.8, 165.7, 165.1, 165.1, 158.5, 156.7, 148.8, 138.7, 138.5, 138.3, 138.3, 138.0, 137.1, 133.6, 133.2, 133.2, 133.2, 133.1, 133.0, 130.2, 130.1, 130.0, 130.0, 129.9, 129.9, 129.9, 129.8, 129.7, 129.6, 129.5, 129.2, 128.8, 128.7, 128.7, 128.6, 128.5, 128.4, 128.4, 128.4, 128.4, 128.3, 128.2, 128.2, 128.2, 128.1, 128.1, 127.9, 127.7, 127.6, 127.6, 127.5, 127.5, 127.5, 127.4, 127.4, 127.3, 127.2, 125.4, 117.2, 108.3, 108.0, 106.9, 100.5, 99.9, 99.7, 98.1, 91.3, 83.2, 81.9, 81.4, 79.9, 79.4, 79.0, 78.7, 77.3, 77.2, 77.0, 76.4, 76.4, 76.1, 74.4, 74.2, 74.0, 73.9, 73.1, 73.0, 72.9, 72.8, 72.6, 72.2, 71.9, 70.6, 70.2, 69.2, 68.4, 67.9, 67.5, 67.4, 67.2, 67.1, 64.0, 61.6, 61.4, 51.3, 34.2, 32.1, 29.9, 29.8, 29.5, 24.9, 22.8, 22.8, 21.6, 21.1, 21.0, 20.8, 17.0, 14.3, 9.3, 1.2, -0.2. HRMS (Q-Tof): calculated for $C_{167}H_{163}F_2NO_{44}Na_2^{2+}$ $[M+2Na]^{2+}$ 1485.0150, found 1485.0155.

4. Deprotections

***β*-D-Galatofuranosyl-(1→3)-4-*O*-[*α*-D-galactopyranosyl-(1→)]-*α*-D-galactopyranosyl-(1→1)-3-amino-2,2-difluoropropanol (1)**

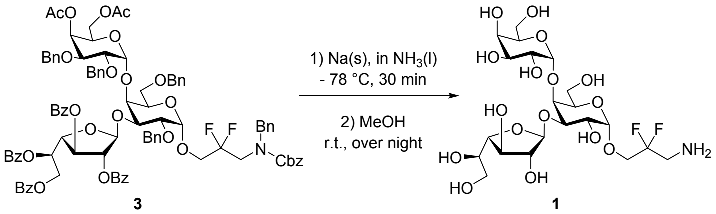

Ammonia gas was liquefied at -78 °C (dry ice-acetone bath) to get ~20 mL NH_3(l) in a 50 mL three-neck flask with a stirring bar in it. Protected trisaccharide **3** (8.5 mg, 5.05 µmol, 1.0 eq.) in a 5 mL flask was dissolved in 0.5 mL THF and a drop of ^{t}BuOH was added as initiator. The solvent of **3** was transferred into the NH_3(l) and the small flask was washed and transferred again with 0.3 mL THF. A thin piece of sodium was cut off and put into the NH_3(l) and the color turned dark blue. The mixture stayed blue so no more sodium was added and it was stirred at -78 °C for 30 min. Then MeOH (1 mL) was added to the mixture dropwise and the solution turned transparent immediately. The mixture was slowly warmed to r.t. and stirred overnight. The mixture was then evaporated, purified with size-exclusion column and HPLC semi-preparation and lyophilized to get final product **1** (0.77 mg) as white solid. Yield: 25%. ^{1}H NMR (700 MHz, Deuterium Oxide) δ 5.24 (d, J = 2.8 Hz, 1H), 5.12 (d, J = 3.8 Hz, 1H), 5.08 (d, J = 3.7 Hz, 1H), 4.27 – 4.20 (m, 3H), 4.18 – 4.08 (m, 4H), 4.08 – 3.96 (m, 4H), 3.96 – 3.89 (m, 2H), 3.89 – 3.82 (m, 3H), 3.82 – 3.75 (m, 2H), 3.72 (dd, J = 11.6, 4.7 Hz, 1H), 3.70 – 3.68 (m, 1H), 3.63 (t, J = 15.4 Hz, 2H). ^{13}C NMR (176 MHz, D_2O) δ 109.0, 100.2, 99.1, 82.1, 80.9, 78.2, 76.5, 76.1, 72.1, 70.9, 70.5, 69.1, 68.9, 67.7, 66.6, 62.7, 60.6, 60.3, 41.5. HRMS (Q-Tof) calculated for $C_{21}H_{37}F_2NO_{16}Na^+$ $[M+Na]^+$ 620.1973, found 620.1978.

***β*-D-Galatofuranosyl-(1→3)-4-*O*-[*α*-D-galactopyranosyl-(1→)]-*α*-D-galactopyranosyl-(1→3)-*β*-D-galatofuranosyl-(1→3)-4-*O*-[*α*-D-galactopyranosyl-(1→)]-*α*-D-galactopyranosyl-(1→1)-3-amino-2,2-difluoropropanol (2)**

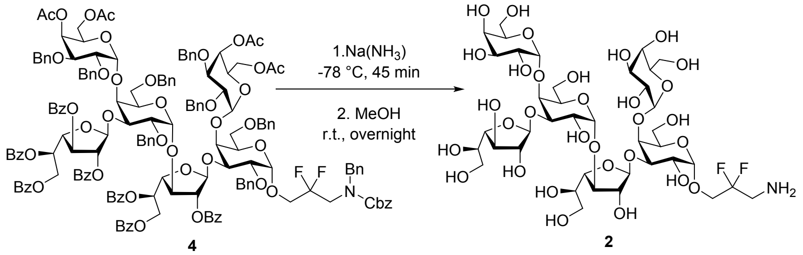

Ammonia was liquefied in a 3-necked flask with a stirring bar in it at -78 °C (dry ice-acetone bath). A small piece of sodium was added to the ammonia(l) and the color of the mixture turned dark blue. Protected hexasaccharide **4** (28 mg, 9.5 µmol) and ^{t}BuOH (0.1 mL) was transferred to Na(NH_3) with anhydrous THF (0.5 mL + 0.2 mL) and the mixture was stirred at –78 °C for 40 min while the color kept dark blue. MeOH was added dropwise until the mixture turned transparent to quench the first step of the reaction. Nitrogen gas was bubbled slowly until

most of the ammonia was removed and the temperature was slowly raised to room temperature. The mixture was stirred overnight, added AcOH to neutralize the solution, evaporated *in vacuo* and purified with Sephadex G-25 and HPLC (hypercarb) to get final product **2** (1.8 mg, 1.7 μmol), yield: 18%. ^{1}H NMR (600 MHz, Deuterium Oxide) δ 5.27 – 5.23 (m, 2H), 5.12 (dt, *J* = 7.7, 2.6 Hz, 2H), 5.07 (d, *J* = 3.8 Hz, 1H), 5.03 (d, *J* = 3.8 Hz, 1H), 4.36 (ddd, *J* = 5.5, 2.9, 1.1 Hz, 1H), 4.32 (ddd, *J* = 8.5, 3.0, 1.1 Hz, 1H), 4.28 – 4.17 (m, 6H), 4.17 – 4.07 (m, 7H), 4.07 – 4.03 (m, 2H), 4.00 (tt, *J* = 10.6, 2.1 Hz, 3H), 3.93 (ddd, *J* = 11.0, 8.9, 3.1 Hz, 4H), 3.90 – 3.82 (m, 7H), 3.82 – 3.79 (m, 1H), 3.79 – 3.76 (m, 2H), 3.75 – 3.66 (m, 4H), 3.64 – 3.55 (m, 2H). ^{13}C NMR (151 MHz, d_2o) δ 170.9, 109.4, 109.0, 100.2, 100.1, 100.0, 98.9, 84.5, 82.0, 80.8, 80.3, 79.6, 78.1, 76.5, 76.4, 76.0, 72.2, 72.0, 70.8, 70.5, 70.5, 69.8, 69.0, 69.0, 68.8, 68.5, 67.8, 67.5, 66.7, 66.5, 66.3, 62.7, 62.6, 60.5, 60.2, 60.2, 59.8, 41.5. HRMS (Q-Tof) m/z $[M+H]^+$ calculated 1084.3738, found 1084.3738.

5. Glycan microarrays

Microarray slides were prepared by coupling the synthetic epitopes **1** and **2** with an amine linker to the Codelink *N*-hydroxysuccinimide-activated glass slides (SurModics Inc., Eden Prairie, USA). The synthetic oligosaccharides were suspended in sterile printing buffer (50 mM PBS buffer, pH 8.5) to a final concentration of 200 μL in a printing plate and loaded into a contact-free piezoelectric microarray robotic spotter (Scienion, Berlin, Germany). The glycans were printed under temperature and humidity-controlled conditions in triplicate. The BSA-adipic linker and carrier protein were also printed in the same slide for unspecific binding controls. Incubation of the resultant slides for 16-24 h in a humidity chamber at room temperature was performed to improve the efficiency of the coupling reaction. The unreacted *N*-hydroxysuccinimide groups on slides were quenched with ethanolamine (100 mM in 0.1 M PBS buffer, pH 9) for 1 h, followed by three washes with ddH_2O for two minutes. After centrifugation at 300x g for 5 min, the microarray was stored at 4 °C in the dark for further use.

When screening, the slide was first blocked with blocking buffer (1% w/v BSA in PBS) for 1 h followed by centrifugation for 5 min at 300x g. A FlexWell grid (FlexWell 64, Grace Bio-Labs, Bend, US) was placed, generating 64 testing fields, where 30 μL of each serum sample (diluted 1:100 in blocking buffer) was applied into individual wells, in triplicate. Incubation was performed for 1 h at 37 °C in a light-prevented humidified box. After the samples were washed three times with 50 μL of washing buffer (PBS + 0.1% Tween-20), 30 μL of secondary fluorescence labeled antibodies (goat anti-human IgG (H + L) Alexa Fluor 647, Thermo Fisher, diluted 1:400 in blocking buffer) were added to each sample and the slides were incubated for another hour at 37 °C. Finally, the samples were washed three times with 50 μL of washing buffer, washed one time with ddH_2O and dried by centrifugation (5 min at 300x g) for fluorescence readout. An Axon GenePix 4300A microarray scanner was used for the fluorescent read outs and the photomultiplier tube (PMT) voltage was adjusted such that the scans were free of saturation signals. The GenePix Pro 7 software (Molecular Devices, Sunnyvale, CA, USA) produced the mean fluorescent intensity values (MFI) that were used for data analysis with R (RStudio Version 1.2.5033) or GraphPad Prism 8 software (Graphpad Software Inc., La Jolla, USA) for graphical visualization.

6. Minimum glycan epitope screening

In order to determine the minimum glycan epitope, the designed trisaccharide and hexasaccharide were incubated with serum of patients infected with *K. pneumoniae* resistant to carbapenems (CR-*Kp*). A total of 20 human sera samples from patients infected with CR-*Kp* was provided by the group of Dr. Bettina C. Fries from Stony Brook University, New York. Two non-infected sera and one pooled human serum from 287 individuals (WHO 007 sp., NIBSC) were used as negative controls and served as a reference for the determination of antibody binding threshold. Patients were consented under institutional review board (IRB) and SBU Human Subjects Committee approved protocols (IRB# 896845 and 851803). The health information was deidentified. Healthy donors gave written informed consent for blood donation under IRB# 718744. After blood collection, the samples were centrifuged, and the sera were transferred to Eppendorf tubes and sent by plane under cooling conditions. Samples were kept at -20°C until further use. Recognition of the glycan antigen by human antibodies present in sera was measured using glycan microarrays mentioned in the previous part.

7. Conjugation

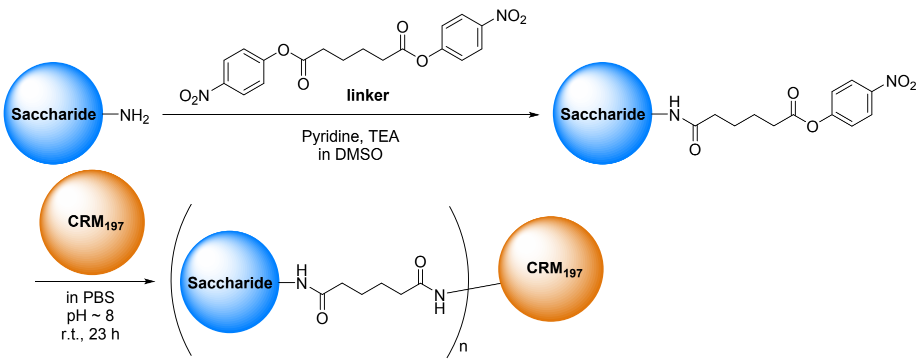

Fig. S1 Conjugation of the saccharides onto CRM_{197}

The conjugation was performed as reported [4]. Hexasaccharide **2** in a vial was dissolved in 200 μL DMSO, into which 25 μL pyridine and 10 μL TEA were added. The linker [bis(4-nitrophenyl) adipate, 4.90 mg] was dissolved in 100 μL DMSO and the solution was added to the saccharide solution. After stirring for 3 h, the stirring bar was removed and the reaction mixture was frozen in liquid nitrogen and lyophilized over night to remove all solvent. The residue was carefully washed with chloroform (5x 1 mL) and DCM (3x 1 mL). TLC (40% EtOAc in Hexane) showed that all excess linker was washed away and no sugar dissolved. The washed residue was redissolved in DMSO, transferred to a 1.5 mL vial and lyophilized again for further conjugation. CRM_{197} (1 mg) was dissolved in autoclaved water (750 μL), transferred into an Amicon 10k filter centrifuged. The filtrate (~60 μL) was transferred to the conjugation vial with phosphate buffer solution (PBS, 2x 30 μL, pH 8) and the solution was stirred at r.t. for 23 h. The solution was then transferred onto an Amicon 10k filter, washed with PBS (4x 400 μL, pH 8) by centrifuge, transferred to a clean vial and diluted with PBS to 400 μL. The solution of conjugated saccharide was kept at 4 °C for further biological study.

CRM_{197}-2

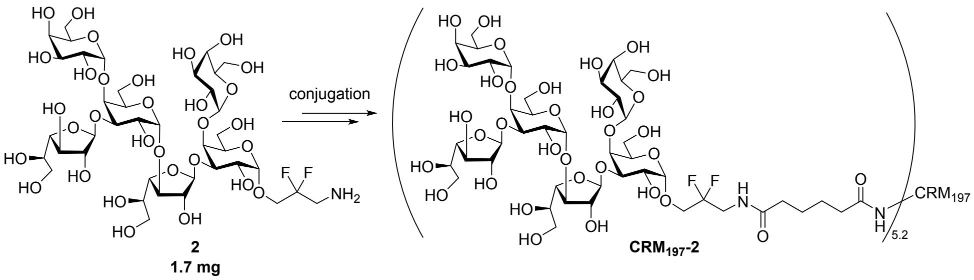

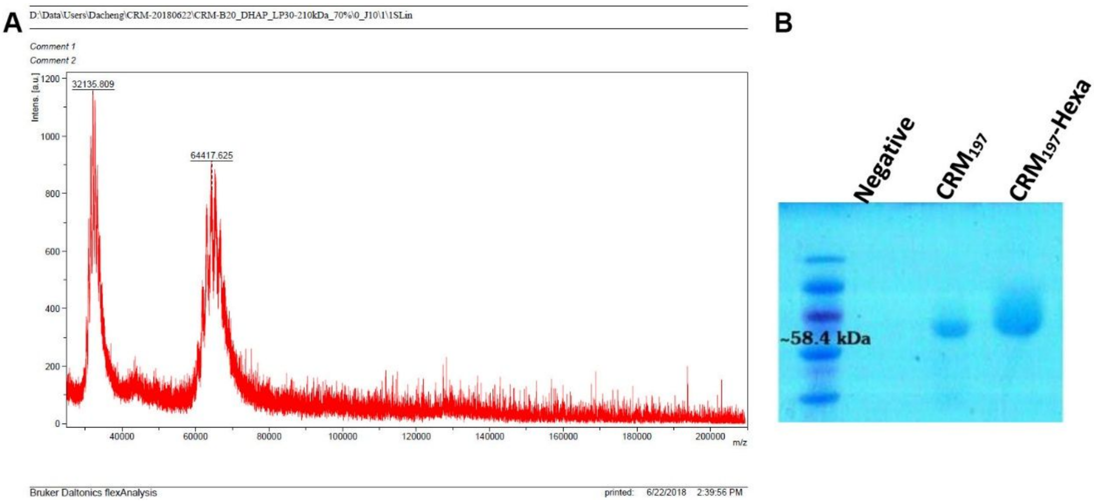

Fig. S2 MALDI (**A**) and SDS-PAGE (**B**) of CRM_{197} and **CRM_{197}-2**

Calculation of the loading number:

MALDI DHAP Matrix of **CRM_{197}-2**: 64417 Da

MALDI DHAP Matrix of CRM_{197}: 58184 Da

Sugar + Linker: 1210 Da

CRM_{197}-2 - CRM_{197} = 6233 Da

Loading Number **n** = 6233 / 1210 = **5.2**

8. Glycoconjugate formulation with aluminum adjuvant

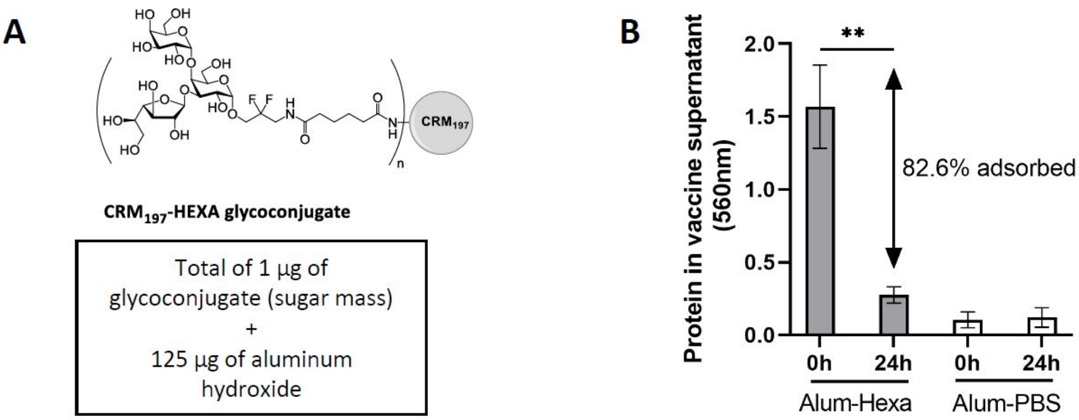

Fig. S3 Glycoconjugate vaccine formulation. Formulation of the glycoconjugate with aluminum hydroxide (Alhydrogel, Brenntag, Denmark) in a final dose of 0.5 mL in PBS (pH 7.4, PAN-Biotech, Germany). (**A**) A total of 1 µg **CRM_{197}-2** (calculated based on the epitope present in glycoconjugate) was adsorbed into 125 µg of aluminum hydroxide. The solution was rotated for 24 h at 4 °C to allow the adsorption to complete the alum matrix. (**B**) Quantification of protein, in the supernatant of vaccines after incubation with alum, was measured by ELISA. The adsorption rate was above 80% when compared to the same vaccine formulation without incubation. Alum and PBS was used as a negative control. containing alum and PBS was used in the assay. The error bars are the SD of three independent assay performed with three different vaccines. Unpaired t-test was used for statistical analysis.

9. Rabbits' immunization and ethical approval

A group of five rabbits (8-week-old Zika rabbits obtained from BioGenes, Berlin, Germany) were immunized with glycoconjugate (1 µg of antigen **CRM_{197}-2** per dose) mixed with aluminum hydroxide as adjuvant (Brenntag, Mülheim, Germany). The negative control group contained three rabbits (BioGenes, Berlin, Germany) that were injected with only PBS with aluminum hydroxide. On days 14 and 28, the rabbits received booster immunizations with the same formulation. Rabbits were bled on days 0, 14, 21, 28 and 35, and the antibody levels were measured. The long-term immune response was measured eleven days after a boost injection on day 133 by analysis of the antibody response. For serum extraction, the collected blood (15-50 mL per animal) from different time points was left to coagulate for at least 30 min at RT and then centrifuged for 15 minutes at 2000x g to separate blood cells from the serum containing antibodies. The serum was transferred to clean vials, aliquot, and stored at -20 °C until the performance of antibody evaluation assays.

All animals were kept according to the governing laws of DIN EN ISO 9001:2000 64 standards, German guidelines according to law 8a Animal Welfare Act of 18. May 2006 (BGBI. I, p.1206), European Union guidelines 86/609/EWG of 24.11.1986 and according to the European Agreement of 18.3.1996 for protection of animal trials and other for scientific purposes used vertebrates of 11.12.1990 (BGBl.ll S. 1486). The immunization was performed in strict

accordance with the NIH/OLAW Animal Welfare Assurance, identification number F16-00178 (A5755-01) and was authorized by LALLF MV (Landesamt für Landwirtschaft, Lebensmittlelsicherheit und Fischerei Mecklenburg-Vorpommern) in accordance to TierSchG and Tierschuutz-Versuchstierverordnung (project #49062 and #49406).

10.ELISA analysis of blood samples

High binding 96-well polystyrene micro titer plates (Corning, USA) were coated overnight at 4 °C with 10 $\mu g \cdot mL^{-1}$ of CRM_{197} in PBS, pH 7.2 (50 µL per well). The plates were washed three times with PBS-T and blocked with 1% BSA in PBS for 1 h at room temperature. After three times of washes with PBS-T, the plates were incubated with each individual rabbit serum at different dilutions in duplicate or triplicate for 1 h at room temperature. The plates were washed four or five times with PBS-T and incubated with horseradish peroxidase (HRP) conjugated anti-rabbit IgG antibodies H+L (Sigma-Aldrich, USA), then washed thoroughly with PBS-T. The absorbance was recorded at 450 nm using an ELISA reader (Infinite® 200 NanoQuant, Tecan, Switzerland).

11. Flow cytometer for bacteria-surface-specific antibody binding

The evaluation of antibody binding to the native bacterial antigen was performed by flow cytometer (FACS) using well-characterized strains of *K. pneumoniae* expressing O2afg or O1 antigen as positive and negative control, respectively, that were kindly provided by Prof. Dr. Chris Whitfield form the University of Guelph and commercially bought from SSI Diagnostica. The binding of antibodies from immunized animals to the bacteria was assessed following a well stablished protocol with modifications [5].

Briefly, bacteria were grown on agar LB plates at 37 °C for 12-16 h and a single colony was transferred into 5 mL Luria Broth (LB) (Roth, art. No. X968.3 or Sigma art. No. L3522) and incubated O/N at 37 °C with shaking (300 rpm). An aliquot of 5 µL was transferred into 4.95 mL of LB in a 50 mL tube and incubated at 37 °C, until reaching the exponential growth phase (~2-3h). Bacterial density was determined by measuring the optical density (OD) of the samples where an OD of 0.1 contains 2×10^8 CFU/mL (based on bacteria growth curve). A total of 1×10^5 bacteria cells was transferred to an Eppendorf, centrifuged (13,000 rpm, 5 min), and washed three times with PBS. The bacteria pellet was suspended with 200 µL of sera diluted 1:10 in PBS-BSA 1%, incubated for 1h at RT or 4°C, and followed by two washing steps with 0.5 mL of PBS-BSA 1% and centrifugation (13,000 rpm, 5 min). The bacterial pellet was resuspended in 200 µL of fluorescent labeled secondary antibody diluted 1:100 (Goat anti-Rabbit IgG (H+L) Cross-Adsorbed Secondary Antibody, Alexa Fluor 635, Invitrogen) and incubated at 4°C for 1h. Washing steps were repeated and the bacterial pellet was resuspended with 1 mL of 4% (wt/vol) PFA following incubation at 20 min at RT for bacteria fixation. After centrifugation at 13,000 rpm for 5 min, the samples were resuspended in 500 µL of PBS-BSA 1% and used for FACS analysis. The FACS CANTO II (BD) was used for the acquisition and the threshold was set at FSC = 200 and SSC = 200 combined with an "AND" logic gate. Positive control single-stained samples were used to set photomultiplier tube (PMT) voltages and 10,000 events were recorded per sample. The acquisition of pure water or 0.22-µm-filtered 1× PBS with records of < 100 events/min ensured that the machine was cleaned. The software FlowJo 10.7.1 (BD) was used for

data analysis. The positive binder quantification was based on the product of the percent of gated events that passed the fluorescence threshold and the median fluorescence intensity (MFI) of those events that passed the threshold.

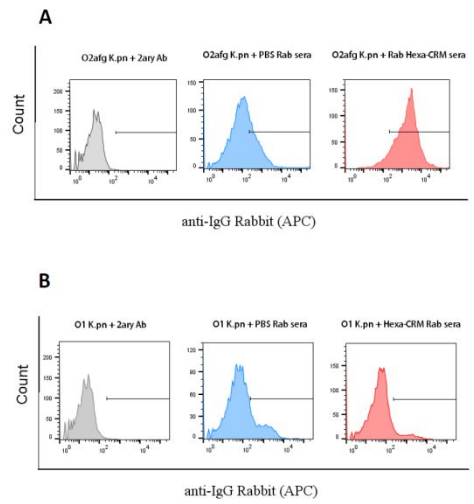

Fig. S4 Bacteria cells bound by the rabbit antibody quantified with a flow cytometer.

K. pneumoniae O2afg or O1 were incubated with sera from day 35 (diluted 1:100) from rabbits immunized with either glycoconjugate (**CRM_{197}-2** group, red) or PBS (PBS group, blue). A negative control containing bacteria with fluorescent secondary antibody (grey) was used as reference to establish the threshold for positive binding. The positive binding quantification (MFI) was based on the product of the percent of gated events that passed the fluorescence threshold and the median fluorescence of those events that passed the threshold. MFI –mean fluorescence intensity, *K. pn –K. pneumoniae*. The error bars represent the SD of three independent experiments. One-way ANOVA was used for statistical analysis. ** $p < 0.01$.

12. *In-vitro* opsonophagocytic killing assay (OPKA)

The assay was performed as described previously [6] with modifications for *K. pneumoniae* since gram-negative bacteria are more prone to complement killing in comparison to gram-positive bacteria. Concisely, the effector HL-60 cell line (a human origin leukemia cell line) was used as phagocytic cell. For granulocyte differentiation, approximately 4×10^5 cells/mL were seeded in tissue culture flasks (Corning, N.Y.) in complete medium (90% RPMI 1640, 10% FCS, 1 mM L-glutamine and penicillin-streptomycin solution; PAN Biotech, Germany) containing 0.8% *N,N*-dimethylformamide (DMF; 99.8% purity; Fisher Scientific, Fair Lawn, N.J.) for 5-6 days at 37 °C in the presence of 5% CO_2. After differentiation, the cells were harvested by centrifugation (300 × g, 5 min) and viable cells were counted by using 1% trypan blue exclusion and resuspended in opsonophagocytic buffer (HBSS with $Ca^{2+}$and Mg^{2+}, 0.1% gelatin, and 10% FBS; HyClone) at a density of 1×10^7 cells/mL. For the opsonophagocytic killing assay, a ratio of 400:1 effector to target cells was used. Glycerol stock of *K. pneumoniae* O2afg grown to mid-log phase (OD600= 0.2–0.3) were gently thawed and diluted in opsonophagocytic buffer to a final density of 1000 CFU per 20 µL. Pooled heated inactivated (56 °C, 30 min) rabbit sera samples (10 µL) were aliquoted in round bottom non-treated 96-well plates in triplicate at four-

fold dilution intervals. The bacterial suspension (20 μL) was added to each well and incubated for 15 min at 37 °C. After, 2.5 % (v/v) baby rabbit complement (Rabbit complement, Cedarlane) and 4×10^5 differentiated HL-60 cells (in 40 μL) were added to each well. The plates were incubated for 45 min at 37 °C in 5% CO_2 with intermittent shaking. The phagocytic reaction was stopped by putting the plate on ice for 15 min. Viable extracellular bacteria were determined by plating aliquots (5 μL) from each well on LB agar plates and incubating at 37°C until visualization of colonies (~12-16 h) for CFU counting. Negative control containing bacteria with complement, HL-60 cells, and buffer, but without antibody, was used as reference to calculate the percent killing of *K. pneumoniae*. The assay was repeated twice independently and in duplicate each. The percent killing was calculated as % phagocytic killing = [(CFU at 0 min − CFU at 120 min)/CFU at 0 min] × 100 and reported as means ± SD of CFU. The opsonic index values where 50% bacterial killing occurs was obtained from four parameters logistic regression of individual opsonic curves [7].

13. Passive immunization in murine pneumonia model

13.1. Rabbit hyperimmune sera preparation

The hyperimmune sera was prepared by pooling the rabbit sera from day 35 (time point after full immunization) from five animals for **CRM_{197}-2**-Alum group and three from PBS-Alum group, separately. The pooled sera were heated at 56 °C for 30 min for complement deactivation, sterilized with 0.22 μm filtration, and stored at -20 °C until usage.

13.2. Bacteria strain characterization and infective sample preparation

Bacteria characterization. The *K. pneumoniae* strain belongs to the ST258 clonal group and expresses KPC-2, TEM-1 & SHV-11 beta-lactamase genes (Leibniz Institute DSMZ, Germany; DSM 113479) In order to determine whether the strain belongs to O2afg serotype, a PCR amplifying specific *O*-antigen genes [8] and the regions of the *gmlABC* operon [9, 10]was performed. A strain expressing O2afg antigen was used as positive control. The boiling method was used for DNA extraction. One single bacterial colony grown on LB agar plate was inoculated in 5 mL LB broth (Roth, cat. No. X968.3 or Sigma, cat. No. L3522) and incubated at 37 °C until exponential phase was reached (~2-3 h). The sample (1 mL) was transferred to an Eppendorf tube and centrifuged at 13,000 rpm for 5 min following two washing steps with RNAse free water (Qiagen, cat. No. 129112). The tube was then placed at 100 °C in a boiling water-bath for 15 min and immediately cooled down on ice. Subsequently, the suspension was centrifuged at 13,000 rpm at 4 °C for 15 min and the supernatant was transferred to a clean 500-μL tube and stored at −20 °C for PCR analysis. For PCR, 50 μg of extracted DNA was mixed with 4 μL of PCR Master Mix containing Mg^{2+} (Thermo Fisher Scientific, Waltham, MA, United States, cat no. F-530XL), Taq polymerase (0.2 μL, Phusion™ High-Fidelity DNA Polymerase, Thermo Fisher, cat No. F-530XL), 0.5 μM primers (**Table S1**), and 200 μM of each dNTP (Invitrogen, cat. No. 18427013) in a final reaction volume of 20 μL. The following cycling conditions was used: 98 °C for 30 s, followed by 20 cycles of 98 °C for 10 s, 57.7 °C for 30 s, and 72 °C for 3 min, then a final extension at 72 °C for 10 min. The samples were stored at -20 °C until use. The DNA fragment size was determined on a 1% agarose gel and the 1Kb plus ladder (Invitrogen, cat. No. 10787018) was used for size reference.

Table S1 Nucleotide sequence of primers used for *O*-serotyping *K. pneumoniae*

Serotype	Primer Name	Nucleotide sequence (5' - 3')	Product lenght (Kb)
**O1/O2**	wb O1/O2-A-F	CGCTATAAGAGCAGCATGCTAG	1.3
	wb O1/O2-A-R	CGATATCACCTACTGCCAGA	
	wb O1/O2-B-F	TTGTTGAGCCTGACAGGATC	1.6
	wb O1/O2-B-R	GCCATTGCTTGCTTGTACAG	
**O3**	wb O3-A-F	CTATCGCTACCGTGGCTTTA	0.8
	wb O3-A-R	TCTCGTCCACAATATCAGCG	
	wb O3-B-F	GCCTACAGTATCTACCTCTG	0.9
	wb O3-B-R	CGGTAAAGTCAGGATGGAAG	
**O4**	wb O4-A-F	CAGAAGCGCGAGTTAATCTG	0.7
	wb O4-A-R	GGTCCAGTTAGGCTCAATTC	
	wb O4-B-F	GTCAGCGGGAATTATTGGAC	1.2
	wb O4-B-R	CTTGAGATCCAGAATGCCAC	
**O5**	wb O5-A-F	GCTACCAAACCAGTATGCTG	1.8
	wb O5-A-R	AGGTGCGTACTGGAAGTATG	
	wb O5-B-F	GGTGATGAAAGCCAGAATGC	1.4
	wb O5-B-R	CAGTGCCTGAAACAGTTTGC	
**O8**	wb O8-A-F	CGTGGCAATGGTTTGCTAGT	1.2
	wb O8-A-R	TCAATCCACACAACTCGGTC	
	wb O8-B-F	GCTAGTTCGGCAACTAACTCAC	0.8
	wb O8-B-R	AGTTCCAGCATCGAAGCAACTC	
**O9**	wb O9-A-F	CGCGCTCAGTTATTCCATTG	1
	wb O9-A-R	CTGGCTGATGACAGAGAATC	
	wb O9-B-F	GCATTCCTGTTCGTGTATGG	0.9
	wb O9-B-R	ATGTCACCGACAGCAAGTAC	
**O12**	wb O12-A-F	CTGCAGATGGCTAAACGTGA	0.6
	wb O12-A-R	CCGTTCGGGCTTGTTCAATA	
	wb O12-B-F	GAAGTCGACTTTGCTGCAGA	1
	wb O12-B-R	ACGTTGATCAAGCTCCTCTC	
**O1**	wbb O1-F	GATTTCACTTTCCGGGCAAC	1.1
	wbb O1-R	GGCTTGCTGAATCACAAGAC	
**O2ac**	wbb O2ac-F	AAACATCGCTGACTCGAGTC	1
	wbb O2ac-R	CGACTATGATCGTACCAACG	
**O2afg**	gmlABC-F	ATGCCAAGTTCAGGCCCATTATG	2.8
***gmlABC* operon**	gmlABC-R	CTAATAATTTATCGTTGACCTTCGCATTG	

13.3. Mice and ethical approval

Animals used in this study were obtained from Charles River (Charles River Laboratories, Sulzfeld – Germany). Conditions for housing and handling of the animals were approved by the German Office for Health and Social Affairs in Berlin (LAGeSo, #A-0242/19), aiming to minimize animal stress. Mice were kept under specific pathogen-free conditions at the internal animal facility at Charité – Universitätsmedizin Berlin (Berlin - Germany) prior to experiment, with a 12/12 hours (h) light/dark cycle, with temperature around 23 °C and humidity close to 40%. The animals were wild type (WT) C57BL/6N, 9–10-week-old female mice, weighting between 19-22 g.

13.4. *In vivo* murine pneumonia model

Study design. Three groups of twelve C57BL/6N mice each were divided into cages containing four animals each. The first group was passively immunized with **CRM_{197}-2**-Alum hyperimmunne sera and infected with *K. pneumoniae* (testing group), the second group was passively immunized with PBS-Alum hyperimmune sera and infected with *K. pneumoniae* (negative control group), and the third group was passively immunized with **CRM_{197}-2**-Alum hyperimmune sera but infected with PBS (antibody control group, PBS-Mock). The study design was performed in a 48-h window. Blood was collected from the animals prior to their intranasal infection with 1x 10^8 CFU of *K. pneumoniae*. A total amount of 100 µL of hyperimmune sera was administered intraperitoneally 2 h after infection and the animals were monitored for body weight and temperature variation every 12 h until final preparation to characterize disease progression. At the 48-h time point, mice were sacrificed and blood, bronchoalveolar lavage fluid (BALF) and lungs were collected for the measurement of CFU density, immune cells counting, cytokines level, myeloperoxidase (MPO) activity, and histopathology of lungs.

Bacteria infective inoculum. The bacteria inoculum used for infecting the mice was prepared as follows. The day before infection, a small number of bacteria was collected from frozen medium (20% glycerol (Merck, Germany) in Todd Hewitt Broth (THB) (BD, Germany) supplemented with 0.5% yeast extract (THY)) and plated on LB or THB agar plates (BD, Germany) and allowed to grow for 8-10 h at 37 °C. Viable colonies were transferred to 10 mL of THY medium added to 10% fetal calf serum (FCS; Gibco, USA) until an optical density (OD) of 0.03-0.04 at 600 nm was achieved. Bacteria were grown to mid-log phase at 37 °C in water bath for around 2 h until an optimal OD of 0.3-0.4 that corresponds to an amount of 0.3-0.4 x 10^9 colony-forming units (CFU) per mL. Total bacteria number in solution was calculated and the solution was centrifuged at 3100 rpm without breaks at RT. The pellet was resuspended in phosphate-buffered saline (PBS; Gibco, USA) for a final concentration of 1 x 10^9 CFU/mL. The final solution was kept on ice until murine infection was performed. A ten-fold serial dilution of the final bacterial solution in PBS was made and four final grade dilutions were plated on LB agar plates, in duplicate, in order to validate the cell density.

Sera titration and mice blood antibody titer. In order to establish the optimal hyperimmune sera dose for passive immunization, three groups of mice with three mice each were passively immunized i.p. with either 1 µL, 10 µL or 100 µL dose of **CRM_{197}-2**-Alum hyperimmune sera. Blood from the animals was collect from the Vena cava caudalis at time 0 h prior immunization and after 48 h post-immunization. Blood was let to sit for 30 min at RT and it was centrifuged for 15 min at 2000 x g to separate blood cells from the serum containing antibodies. The antibody titers against the glycan antigen were evaluated by glycan microarray.

Intranasal infection. Mice were anesthetized i.p. with 3 mL/kg of a mixture of 100 mg/mL ketamine (CP-pharma, Germany) and 20 mg/mL xylazine (CP-pharma, Germany), diluted in 0.9% NaCl solution according to their weight. Under anesthesia and without reflexes, mice were hanged by their teeth on a line to reach a vertical position and allow exposure of their nostrils. To avoid eye dryness, a small portion of 3 mg/g Thilo-tears gel (Alcon, USA) was applied in the ocular region of the animals. The bacteria stock solution was diluted 1:1 with hyaluronidase (Sigma-Aldrich Chemie GmbH, USA) to facilitate bacterial colonization of the lungs and mice were infected intranasally with 20 µL of bacteria. The mock infected control group was inoculated with the same volume of PBS. Infection was performed in two steps, to

avoid blockage of the nasal cavity of the mouse. Altogether, mice were infected with 1 x 10^8 CFU of *K. pneumoniae* and placed back in cages heated with infrared light, for the waking phase after infection.

Animal preparation and sample collection. At the 48-h time point, the animals were prepared for sample collection (blood, BALF, lung) as follows. Mice were anesthetized with 160 mg/kg ketamine and 75 mg/kg xylazine. Under deep narcosis, animals were prepared for collection of the material desired. Blood was collected from the caudal vena cava in split in a tube containing ethylenediaminetetraacetic acid (EDTA)-K (Sarstedt, Germany) or nothing for serum collection. Small blood aliquots were kept for bacteria counting and for flow cytometry analysis. The remaining blood was centrifuged at 4 °C for ten minutes with 4000 x g and the plasma collected was frozen for quantification of cytokines. For BALF collections, the lungs were exposed and the trachea was carefully released. To fix the BAL tube, a surgical thread was passed around the trachea. Using fine dissection scissors, a small incision was made at the posterior part of the trachea, the tube was inserted into the trachea and fixed by the surgical knot. The BAL tube was attached to the trachea and a solution of 1000 µL of PBS containing complete Mini protease inhibitors (Roche Diagnostics GmbH, Germany) used for the acquisition of the bronchoalveolar lavage fluid (BALF). A small aliquot was used for bacteria counting and FACS analysis and tube was centrifuged for ten minutes at 4 °C with 300 x g. The supernatant was frozen in liquid nitrogen for later analysis of antibody level, cytokines expression, and MPO activity. For lung collection, the lungs were perfused with PBS injected through the heart right ventricle after cutting the inferior vena cava in the thorax region. Lungs were washed until the eluent became whitish to avoid blood cell contamination of the sample. The lung lobes were prepared and kept in a solution of PBS and protease inhibitors. Then cut into smaller pieces and homogenized through a 100 µm filter for CFU counting.

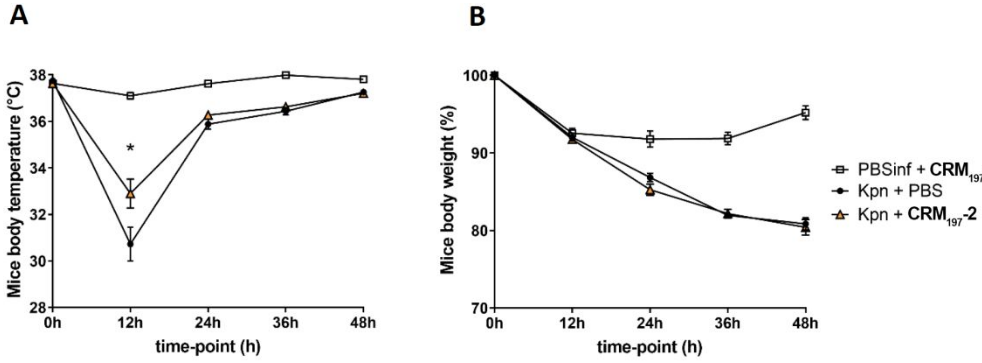

Fig. S5 Anti-O2afg polyclonal sera improve body temperature in mice after infection with CR-*Kp*. Micebody temperature and body weight was inferred before infection (0 h) and every twelve hours post-infection. Infected mice (Kpn) were passively immunized with polyclonal sera of rabbits immunized with either synthetic anti-O2afg vaccine (**CRM_{197}-2**) or PBS vaccine (PBS) two hours post-infection with 1x10^8 of O2afg-CR-*Kp*. Mice infected with PBS (PBSinf) but receiving **CRM_{197}-2** sera was used as a negative control. (**A**) Mouse body temperature. (**B**) Mouse body weight. The data represents the mean ± SE of 12 mice per group. Unpaired t-test was used for the statistical analysis. *$p < 0.05$.

13.5. Measurements of immune cells in blood and BALF

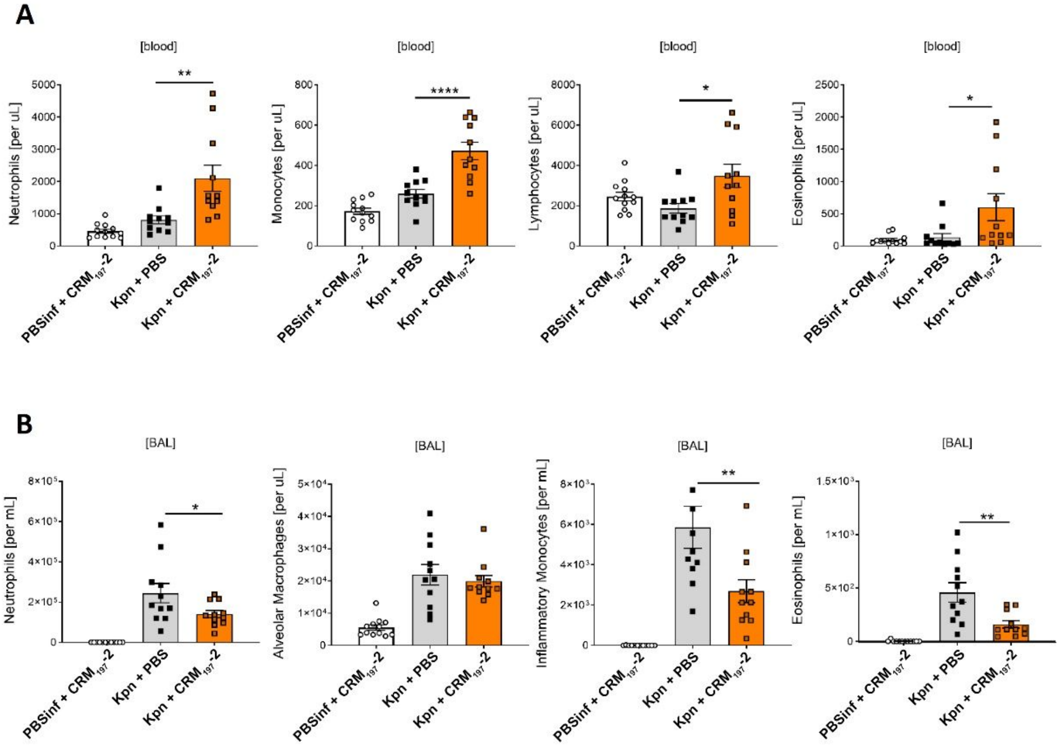

Fig. S6 Anti-O2afg polyclonal induce immune cells in blood and reduce cell infiltration in BALF of mice infected with CR-*Kp*. Blood and BALF were collected 48 hours after infection. (**A**) Neutrophils, monocytes, lymphocytes, and eosinophils were quantified by flow cytometry in blood. (**B**) Neutrophils, alveolar macrophages, inflammatory monocytes, and eosinophils l were also quantified in BALF. The data represents the mean ± SE of 12 mice per group. One-way ANOVA was used for the statistical analysis. * $p < 0.05$ ** $p < 0.01$ ****$p < 0.0001$. One mouse was excluded from Kpn infected PBS group due to death after narcosis and one mouse outlier was excluded from the Kpn infected **CRM_{197}-2** group.

Flow cytometry was used to identify specific cell phenotypes from blood and BALF of passively immunized mice. For FACS analysis, erythrocyte lysis was performed prior to staining with red cell lysis buffer (containing 0.01 M $KHCO_3$, 0.155 M NH_4Cl and 0.1 mM EDTA diluted in distillated water). The cell populations were labeled with the following monoclonal antibodies: phycoerythrin (PE) anti-F4/80 (BM8), PE-cyanine 7 (PE-Cy7) anti-CD11b (M1/70), Alexa Fluor 700 (A700) anti-MHC Class II (I-A/I-E) (M5/114-15-2) (purchased from eBioscience, USA), A700 anti-CD3 (17A2) (Invitrogen, USA), fluorescein isothiocyanate (FITC) anti-Ly-6G (1A8), Brilliant Violet 510 (BrV510) anti-Ly-6C (HK1.4) (purchased from BioLegend,USA), BrV510 anti-CD19 (1D3), Brilliant Violet 421 (BrV421) anti-Siglec-F (E50-2440), Pacific Blue V450 anti-Ly-6C (AL-21), peridinin chlorophyll protein (PerCP)-Cyanine 5.5 anti-Ly-6G (1A8), anti-Siglec-F (E50-2440), APC anti-NK1.1 (NKR-P1B/NKR-P1C) (PK136), anti-CD11c (HL3) (purchased from BD Bioscience, Germany). Quantification of cells from the *in vivo* samples was performed by addition of counting beads (Invitrogen, USA). Then, cells were analyzed for size (FSC) and granularity (SSC) and doublets were excluded. Then, the followed strategy was used: CD3, CD19 and NK1.1 were used as lineage markers; neutrophils identified as Ly-6G+CD11b+

cells; eosinophils as siglec-F+CD11b+Ly-6C-/lowF4/80+ SSChigh; inflammatory monocytes as Ly-6ChighCD11c-Ly-6GCD11b+ F4/80+; monocytes and macrophages as Ly-6C+CD11b+MHC-classII+F4/80+ and alveolar macrophages as siglec-F+CD11c+F4/80+ cells. Stained cells were analyzed with a BD FACSCanto™ II employing FACSDiva Software (BD Biosciences, Germany). The data obtained was analyzed using the FlowJo software (Tree Star Inc., USA).

13.6. Cytokine and chemokine quantification

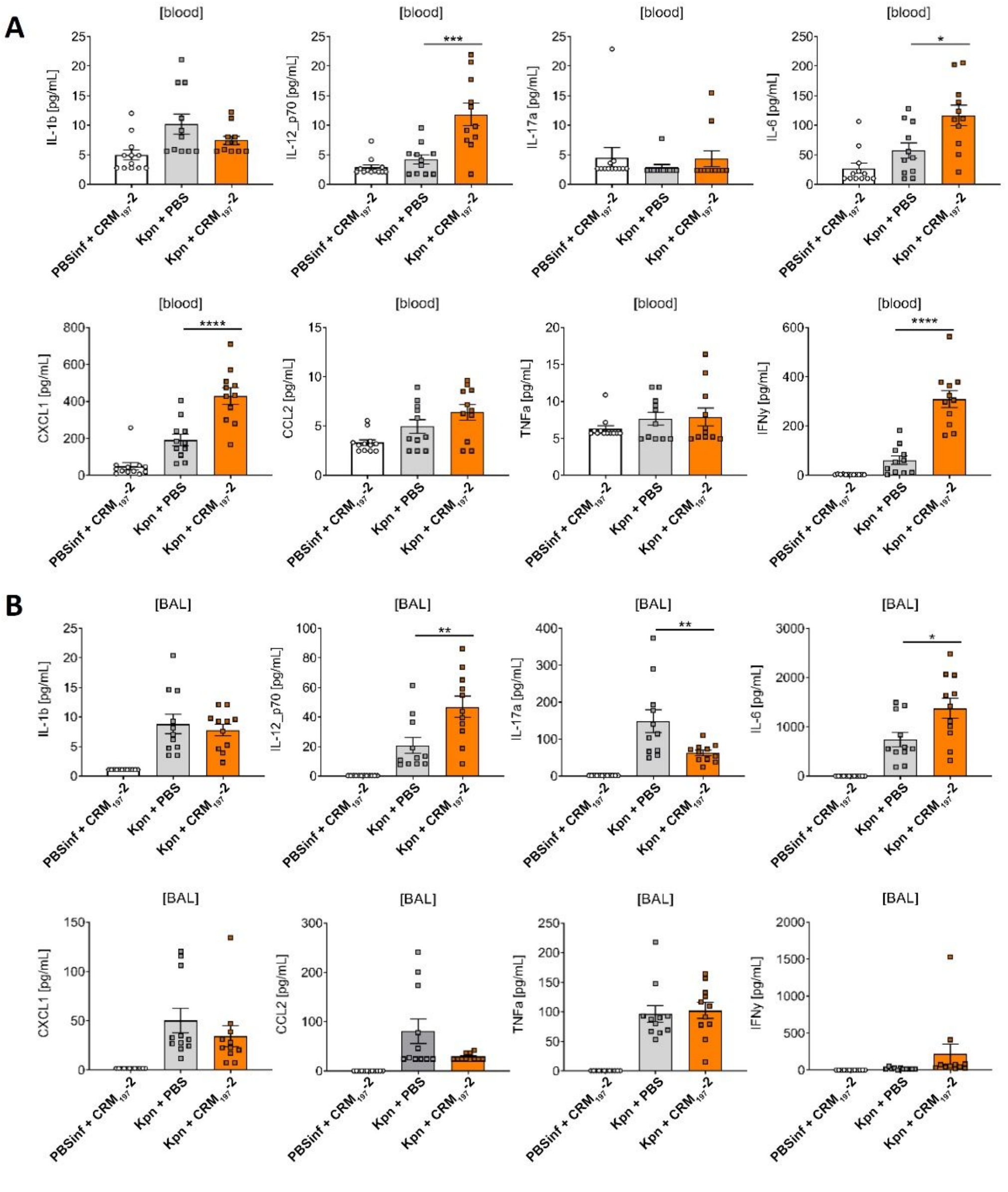

Fig. S7 Pro-inflammatory cytokine and chemokine quantification in blood and BALF of mice infected with CR-*Kp*. Blood and BALF were collected 48 hours after infection. (**A**) Levels of IL-1β, IL-12, IL-17, IL-6, CXCL1, CCL2, TNF-α, IFN-γ were analyzed in blood (**B**) and in BALF. The data represents the mean ± SE of 12 mice per group. One-way ANOVA was used for the statistical analysis. * $p < 0.05$ ** $p < 0.01$ ****$p < 0.0001$. One mouse was

excluded from Kpn infected PBS group due to death after narcosis and one mouse outlier was excluded from the Kpn infected **CRM_{197}-2** group.

Sera collected from animals after preparation were quantified for cytokine levels using beads in a multiplex assay (LEGENDPlex^{TM} multiplex assay, Biolegend, San Diego, CA). A customized panel containing the following cytokines and chemokines capture antibodies was used: IL-17α, TNF-α, IFN-γ and IL-1β, which are pro-inflammatory cytokines, MIP-1α, CCL-2 (MCP-1), which belongs to the macrophage activation pathway, CXCL1 (Gro-α), produced by epithelial cells in response to LPS and TNF-α stimulation and acting as a chemoattractant of neutrophils and IL-12, a pro-inflammatory cytokine involved in B cells and T cell activation. The assay was performed following manufacturer instructions and the data was analyzed using the software provided by the manufacturer.

13.7. Permeability assay

The permeability of the pulmonary vascular barrier was assessed by quantifying the total amount of proteins present in the alveolar space that increases upon breakdown of the alveolar/vascular barrier. The DCTM Protein Assay (Bio-Rad Laboratories Inc., USA) reagents were mixed with BALF samples or standards, according to the manufacturer's instructions and incubated for 15 minutes at room temperature. After reaction, the plate was analyzed at 750 nm using a plate reader SpectraMax M2e (Molecular Devices, USA).

13.8. Myeloperoxidase (MPO) activity assay

The level of neutrophil activation and inflammation can be estimated by the activity of the granulocyte enzyme called myeloperoxidase (MPO) released by these cells upon activation. To quantify MPO activity present in BALF samples, a quantification assay was performed based on the incubation of the samples with 0.75 mM hydrogen peroxide and TMB for five minutes at 37 °C for the break of the TMB substrate by the enzyme. The TMB reaction was stopped with 2 M H_2SO_4 and the plate was measured with 450 nm wavelength using a plate reader SpectraMax M2e (Molecular Devices, USA).

13.9. Histopathology

After trachea ligation was performed to avoid alveoli collapse, lungs were carefully harvested and fixed in 4% paraformaldehyde solution (pH 7.0) and sent to the Department of Veterinary Pathology at Freie Universität Berlin for histopathology analysis. The lungs were embedded in paraffin, and cut into 2-5 μm thick sections followed by hematoxylin and eosin (H&E) staining. Tissue samples were immersed three times in xylene (Chemsolute Xylol Technisch, Th. Geyer, Germany) for twice two min, and three min and then in a series of ethanol solutions (Berkel, Germany) of decreasing concentrations (96%, 80%, and 70%) for 30 seconds. Samples were washed in water, stained with hematoxylin (Roth, Germany) for eight mins, washed in water for five minutes and dyed in eosin (Waldeck, Germany) for 30 seconds. Samples were immersed in increasing ethanol concentrations (70%, 80%, 96%, and 100%) to eliminate the water. Finally, the ethanol was replaced by xylene by four immersions of one minute each in xylene solution. Scanning of HE-stained slides was performed by Aperio CS2 slide Scanner (Leica Biosystems Imaging Ins., CA, USA). The degree of edema formation was assessed semi quantitatively (0 = no edema, 1 = minimal edema, 2 = mild edema, 3 = moderate edema, 4 =

severe edema). Three evenly distributed sections per lung were microscopically evaluated to assess edema formation. Histopathology examination was performed by a European College of Veterinary Pathologists (ECVP) board-certified pathologist, who were blinded to study groups.

14. References

(1) Hahm, H. S.; Hurevich, M.; Seeberger, P. H. Automated assembly of oligosaccharides containing multiple cis-glycosidic linkages. *Nat. Commun.* **2016**, *7*, 12482.
(2) Saikam, V.; Dara, S.; Yadav, M.; Singh, P. P.; Vishwakarma, R. A. Dimethyltin Dichloride Catalyzed Regioselective Alkylation of cis-1,2-Diols at Room Temperature. *J. Org. Chem.* **2015**, *80* (24), 11916-11925.
(3) Deng, L. M.; Liu, X.; Liang, X. Y.; Yang, J. S. Regioselective glycosylation method using partially protected arabino- and galactofuranosyl thioglycosides as key glycosylating substrates and its application to one-pot synthesis of oligofuranoses. *J. Org. Chem.* **2012**, *77* (7), 3025-3037.
(4) Wu, X.; Ling, C. C.; Bundle, D. R. A new homobifunctional p-nitro phenyl ester coupling reagent for the preparation of neoglycoproteins. *Org. Lett.* **2004**, *6* (24), 4407-4410.
(5) Moor, K.; Fadlallah, J.; Toska, A.; Sterlin, D.; Balmer, M. L.; Macpherson, A. J.; Gorochov, G.; Larsen, M.; Slack, E. Analysis of bacterial-surface-specific antibodies in body fluids using bacterial flow cytometry. *Nat. Protoc.* **2016**, *11* (8), 1531-1553.
(6) Burton, R. L.; Nahm, M. H. Development and validation of a fourfold multiplexed opsonization assay (MOPA4) for pneumococcal antibodies. *Clin. Vaccine Immunol.* **2006**, *13* (9), 1004-1009.
(7) Dwyer, M.; Gadjeva, M. Opsonophagocytic assay. *Methods Mol. Biol.* **2014**, *1100*, 373-379.
(8) Fang, C. T.; Shih, Y. J.; Cheong, C. M.; Yi, W. C. Rapid and Accurate Determination of Lipopolysaccharide O-Antigen Types in Klebsiella pneumoniae with a Novel PCR-Based O-Genotyping Method. *J. Clin. Microbiol.* **2016**, *54* (3), 666-675.
(9) Clarke, B. R.; Ovchinnikova, O. G.; Kelly, S. D.; Williamson, M. L.; Butler, J. E.; Liu, B.; Wang, L.; Gou, X.; Follador, R.; Lowary, T. L.; et al. Molecular basis for the structural diversity in serogroup O2-antigen polysaccharides in Klebsiella pneumoniae. *J. Biol. Chem.* **2018**, *293* (13), 4666-4679.
(10) Szijarto, V.; Guachalla, L. M.; Hartl, K.; Varga, C.; Banerjee, P.; Stojkovic, K.; Kaszowska, M.; Nagy, E.; Lukasiewicz, J.; Nagy, G. Both clades of the epidemic KPC-producing Klebsiella pneumoniae clone ST258 share a modified galactan O-antigen type. *Int. J. Med. Microbiol.* **2016**, *306* (2), 89-98.
