# Supplementary material for: Semisynthetic Glycoconjugate Vaccine Lead against Klebsiella pneumoniae Serotype O2afg Induces Functional Antibodies and Reduces the Burden of Acute Pneumonia
Source: J Am Chem Soc. 2024 Dec 12;146(51):35356–66. doi: 10.1021/jacs.4c13972 (PMC11673581; doi:10.1021/jacs.4c13972)

Supplementary Materials for
A semi-synthetic glycoconjugate vaccine lead against *Klebsiella pneumoniae* serotype O2afg induces functional antibodies and reduces the burden of acute pneumonia

Dacheng Shen, Bruna M. S. Seco, Luiz Gustavo Teixeira Alves, Ling Yao, Maria Bräutigam, Bastian Opitz, Martin Witzenrath, Bettina C. Fries, Peter H. Seeberger*

*Corresponding Author: peter.seeberger@mpikg.mpg.de

The file includes:

NMR spectra of new compounds

^{1}H-NMR of **1**

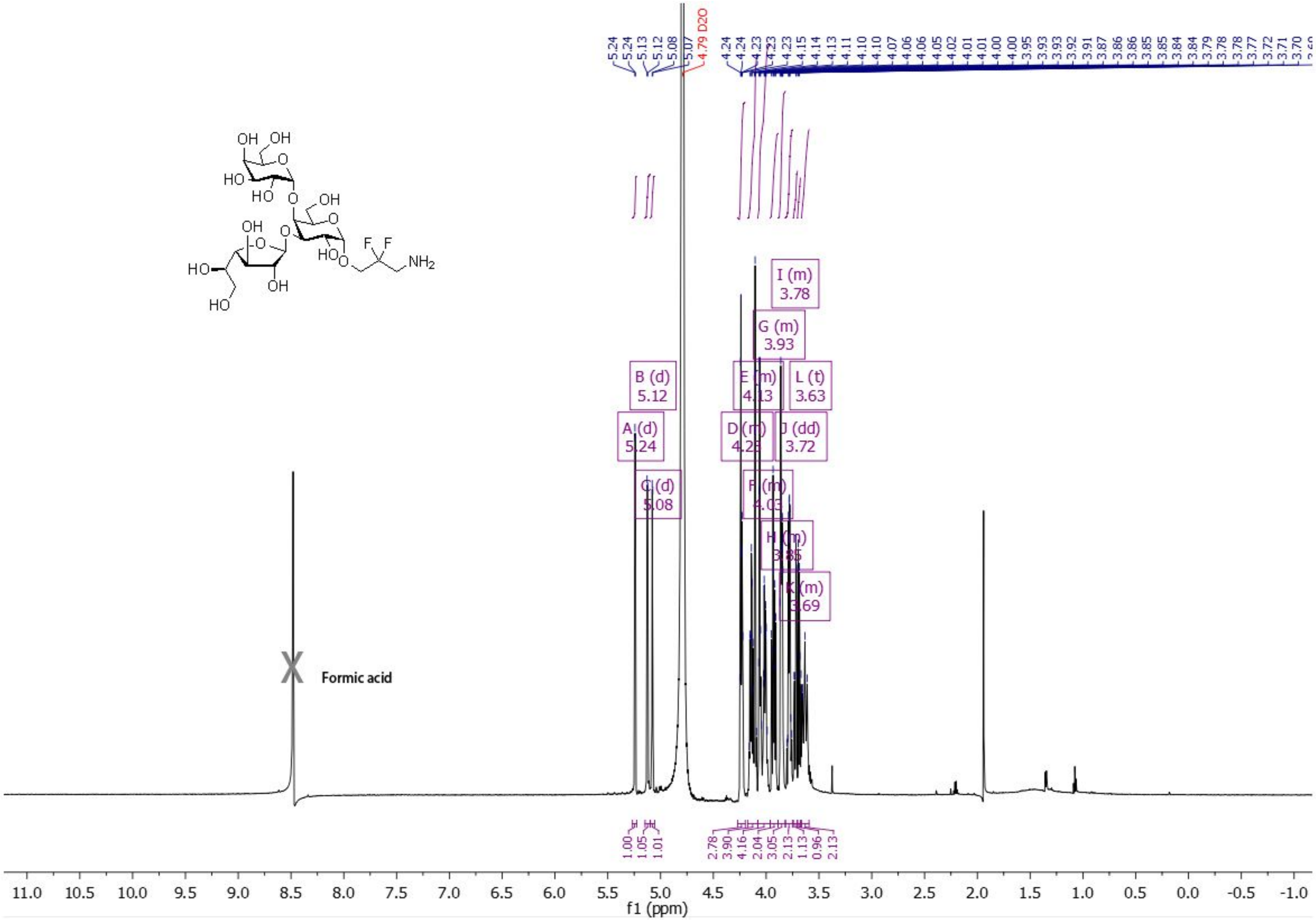

Formic acid
A (d) 5.24
B (d) 5.12
C (d) 5.08
D (m) 4.23
E (m) 4.13
F (m) 4.03
G (m) 3.93
H (m) 3.85
I (m) 3.78
J (dd) 3.72
K (m) 3.69
L (t) 3.63
4.79 D2O
f1 (ppm)

^{13}C-NMR of **1**

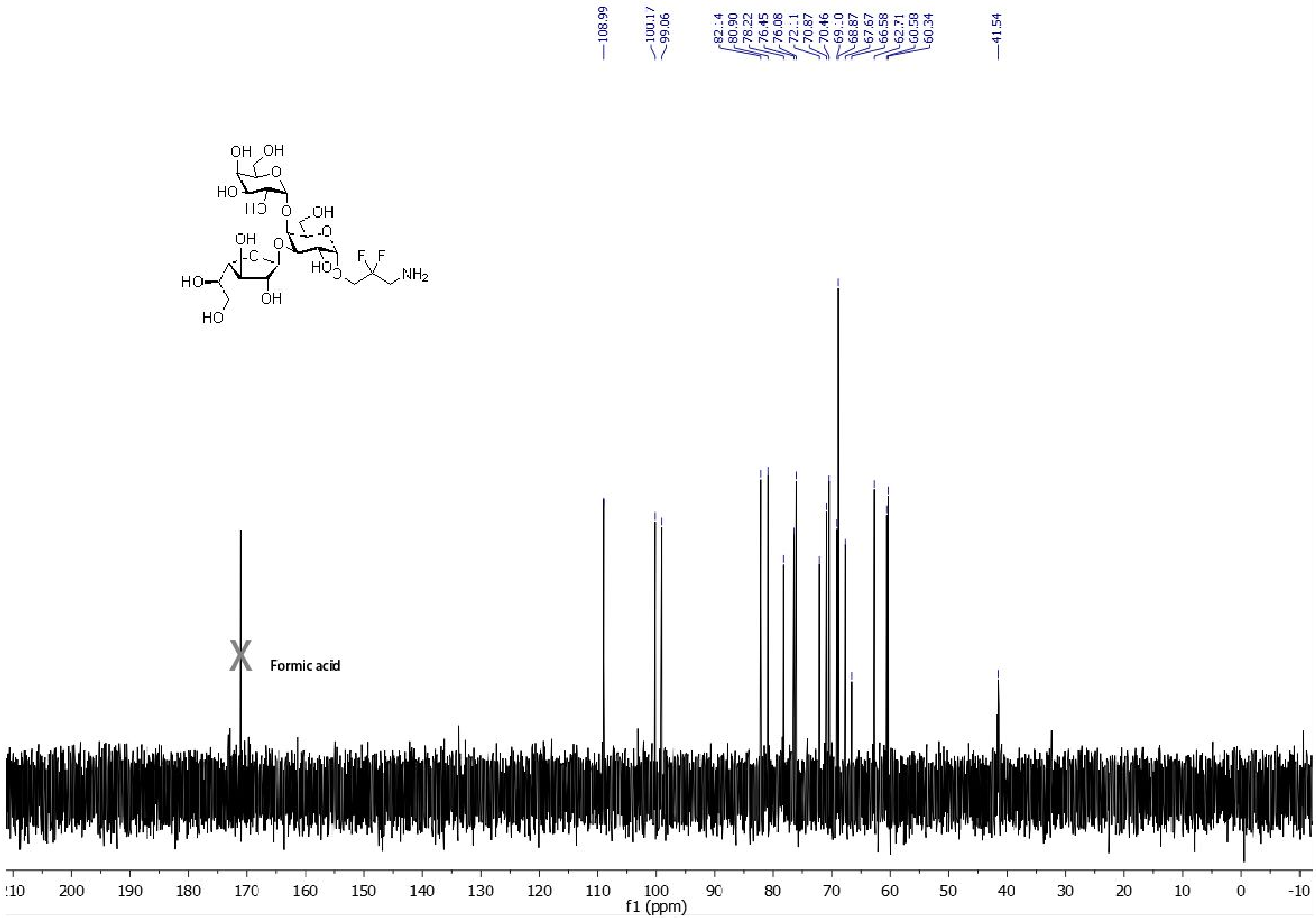
108.99
100.17
99.06
82.14
80.90
78.22
76.45
76.08
72.11
70.87
70.46
69.10
68.87
67.67
66.58
62.71
60.58
60.34
41.54
Formic acid
f1 (ppm)

H-C HSQC of **1**

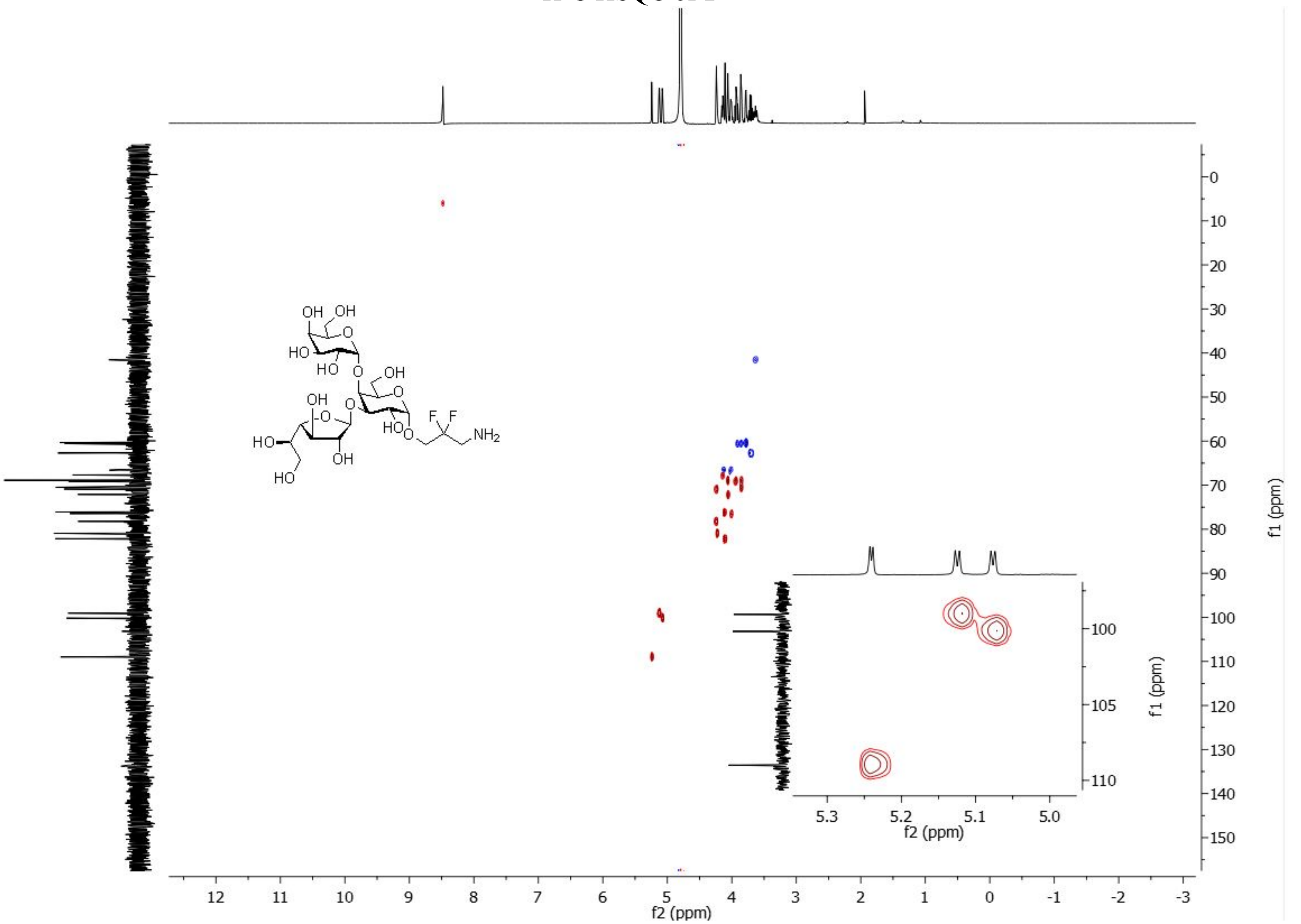

^{1}H-NMR of **2**

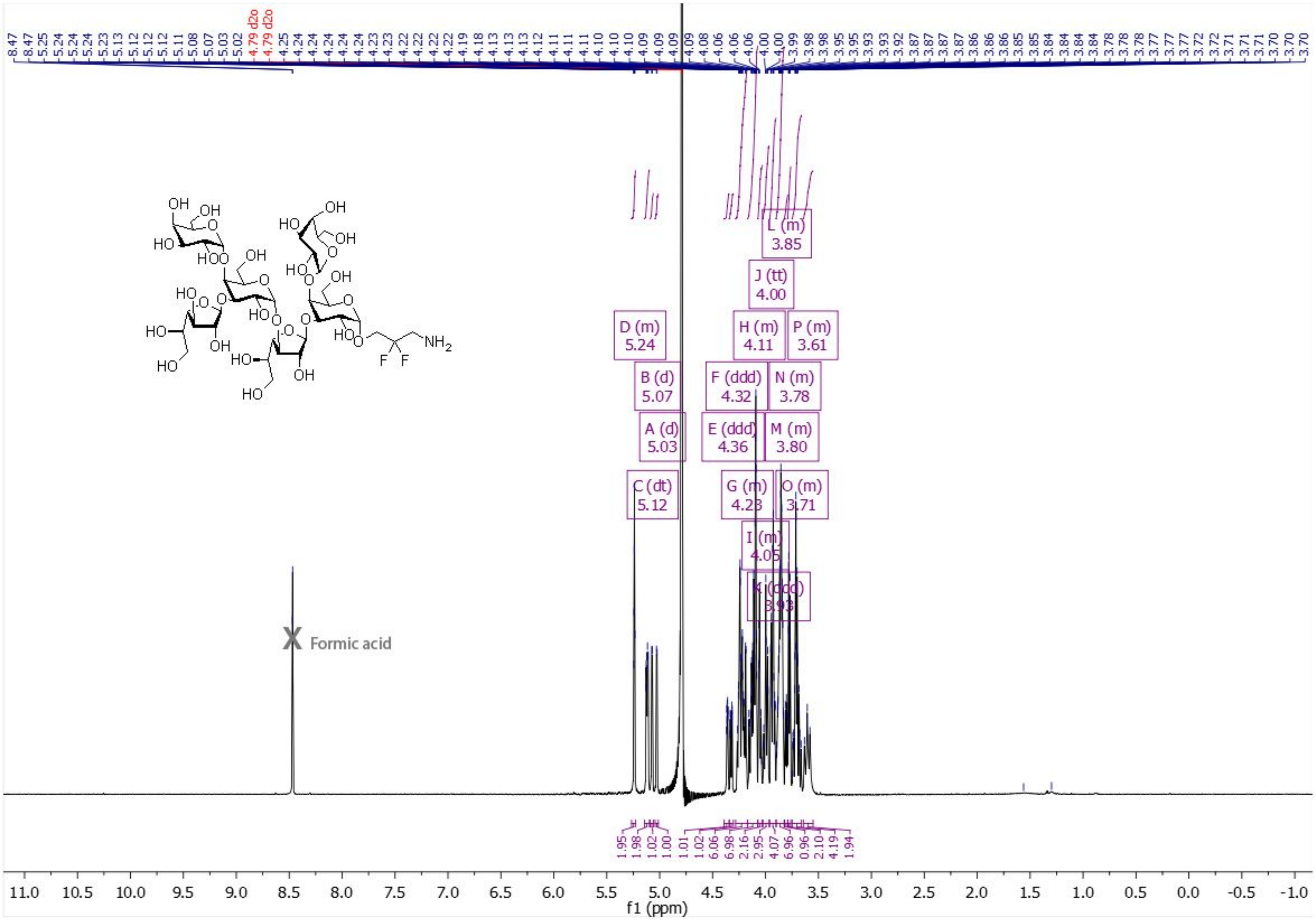
Formic acid
D (m) 5.24
B (d) 5.07
A (d) 5.03
C (dt) 5.12
L (m) 3.85
J (tt) 4.00
H (m) 4.11
P (m) 3.61
F (ddd) 4.32
N (m) 3.78
E (ddd) 4.36
M (m) 3.80
G (m) 4.23
O (m) 3.71
I (m) 4.05
K (ddd) 3.93
f1 (ppm)

^{13}C-NMR of **2**

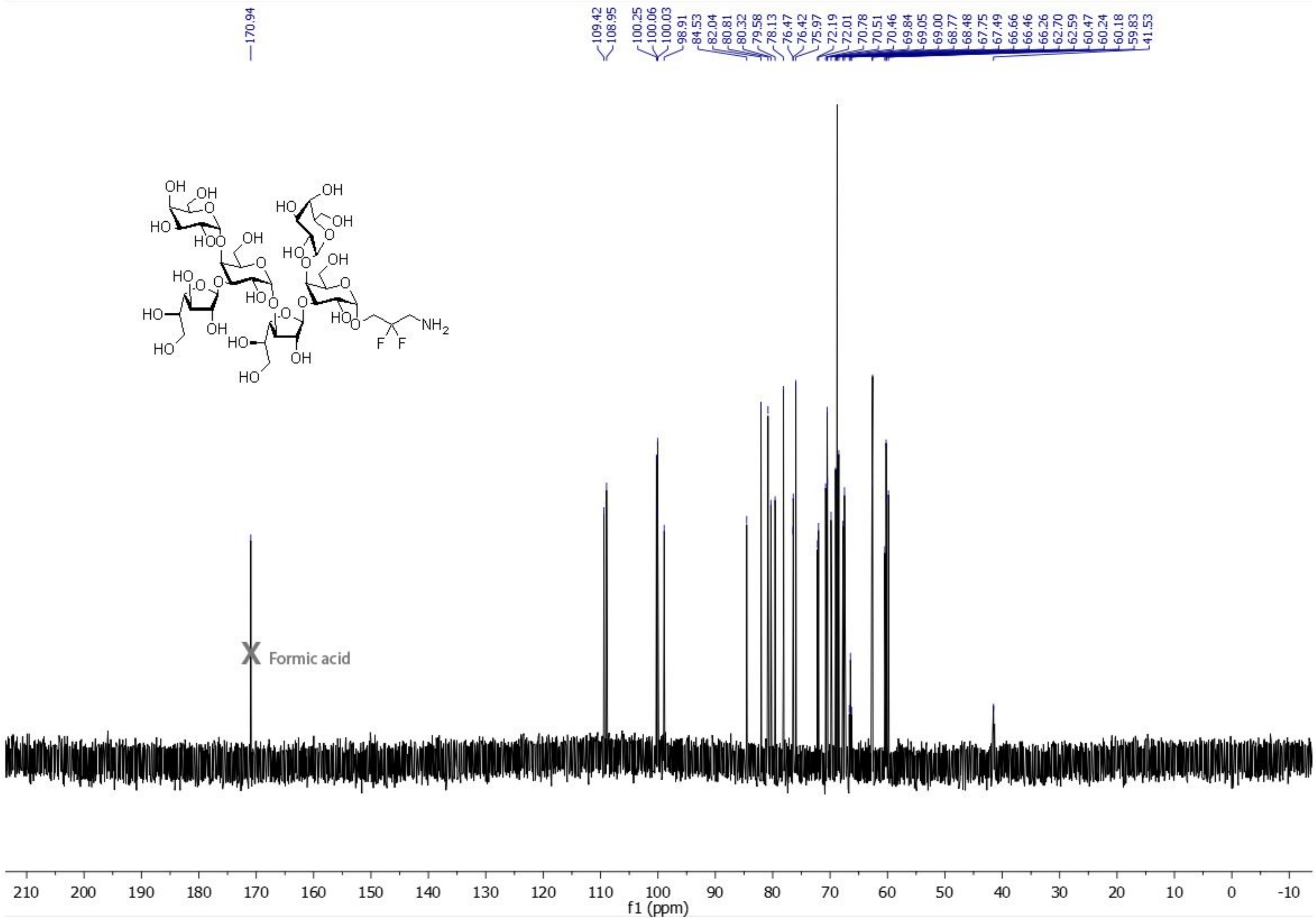
170.94
109.42
108.95
100.25
100.06
100.03
98.91
84.53
82.04
80.81
80.32
79.58
78.13
76.47
76.42
75.97
72.19
72.01
70.78
70.51
70.46
69.84
69.05
69.00
68.77
68.48
67.75
67.49
66.66
66.46
66.26
62.70
62.59
60.47
60.24
60.18
59.83
41.53
X Formic acid
210 200 190 180 170 160 150 140 130 120 110 100 90 80 70 60 50 40 30 20 10 0 -10
f1 (ppm)

H-C HSQC of **2**

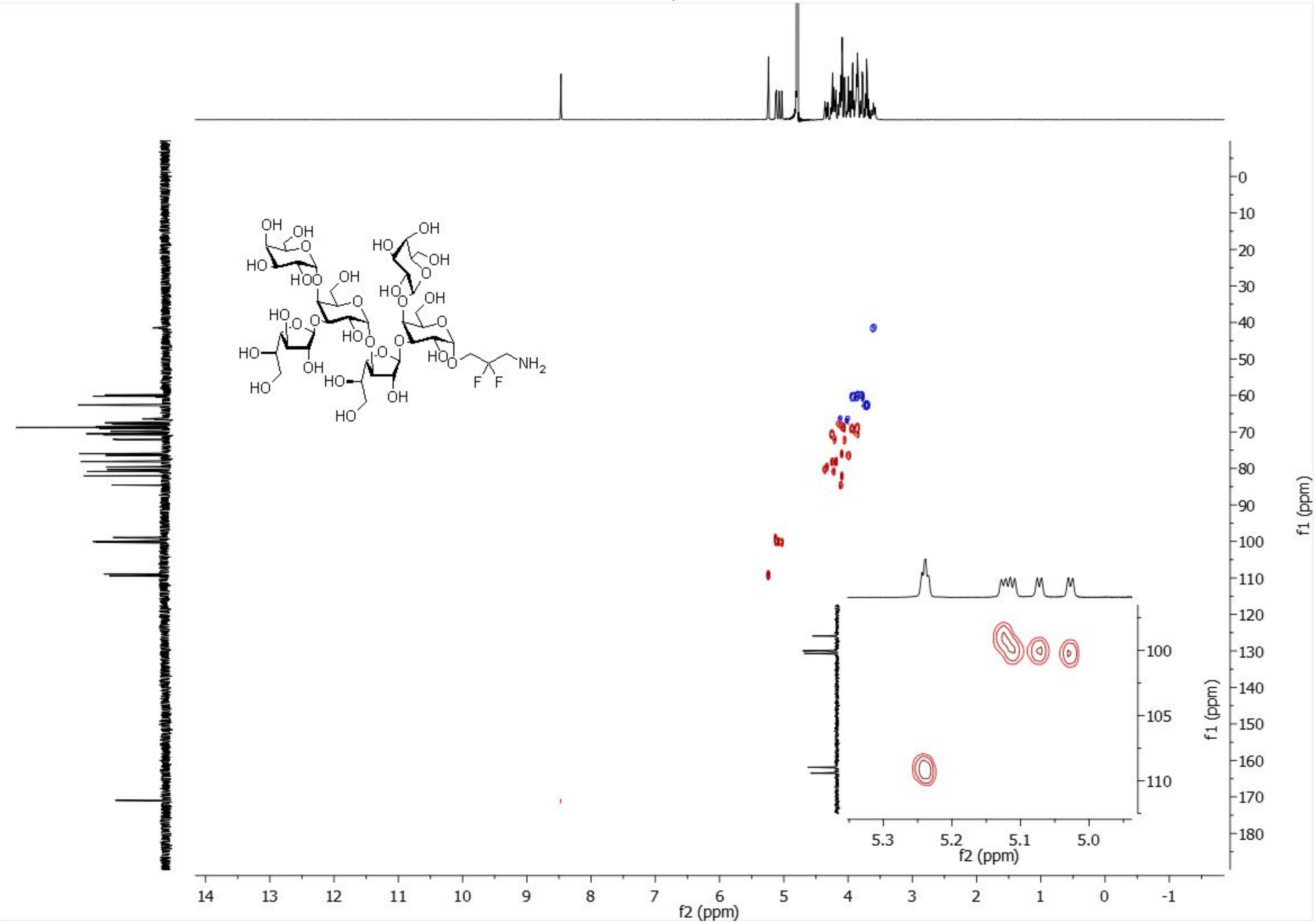

Coupled H-C HSQC of **2**

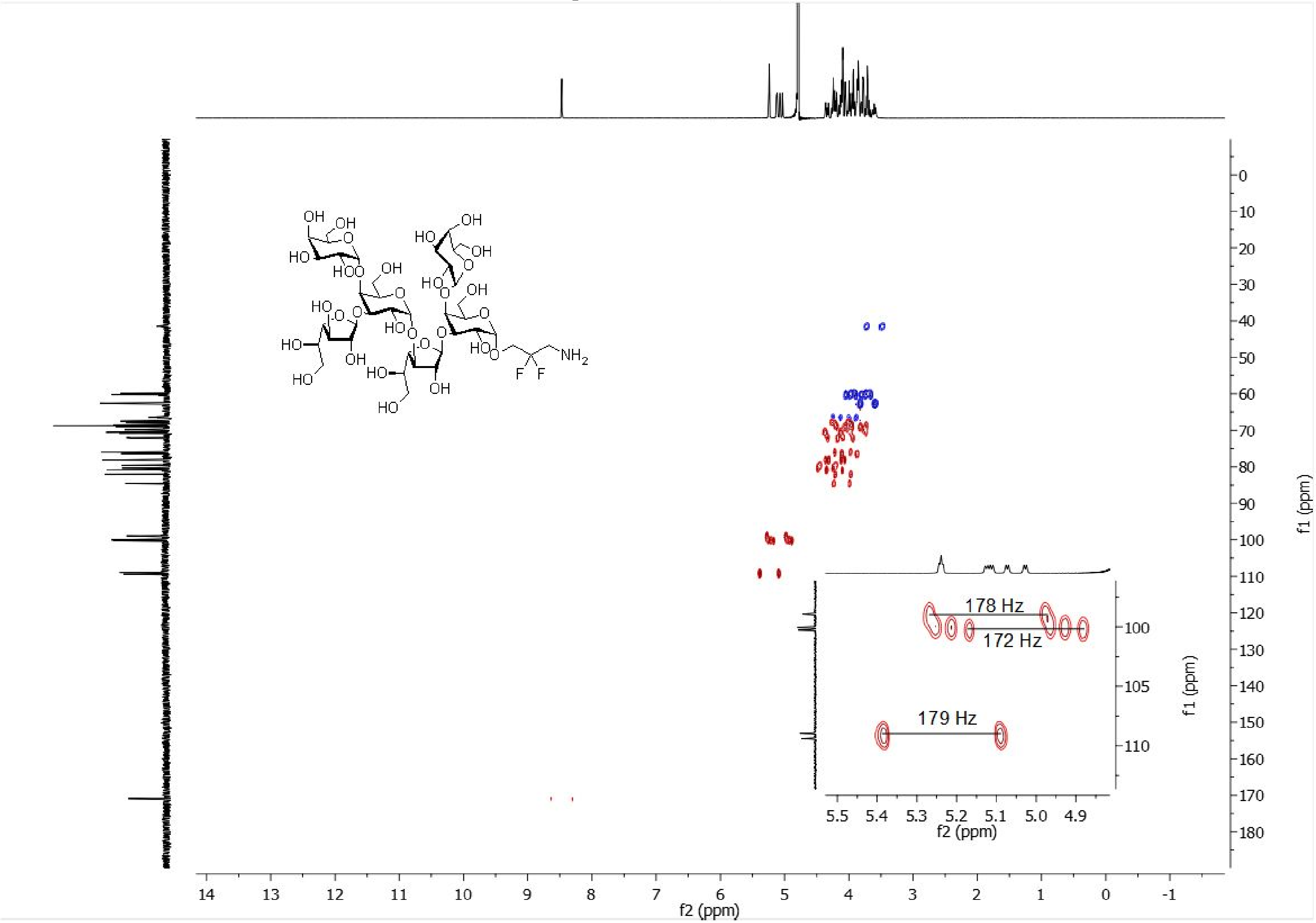

^{1}H-NMR of **3**

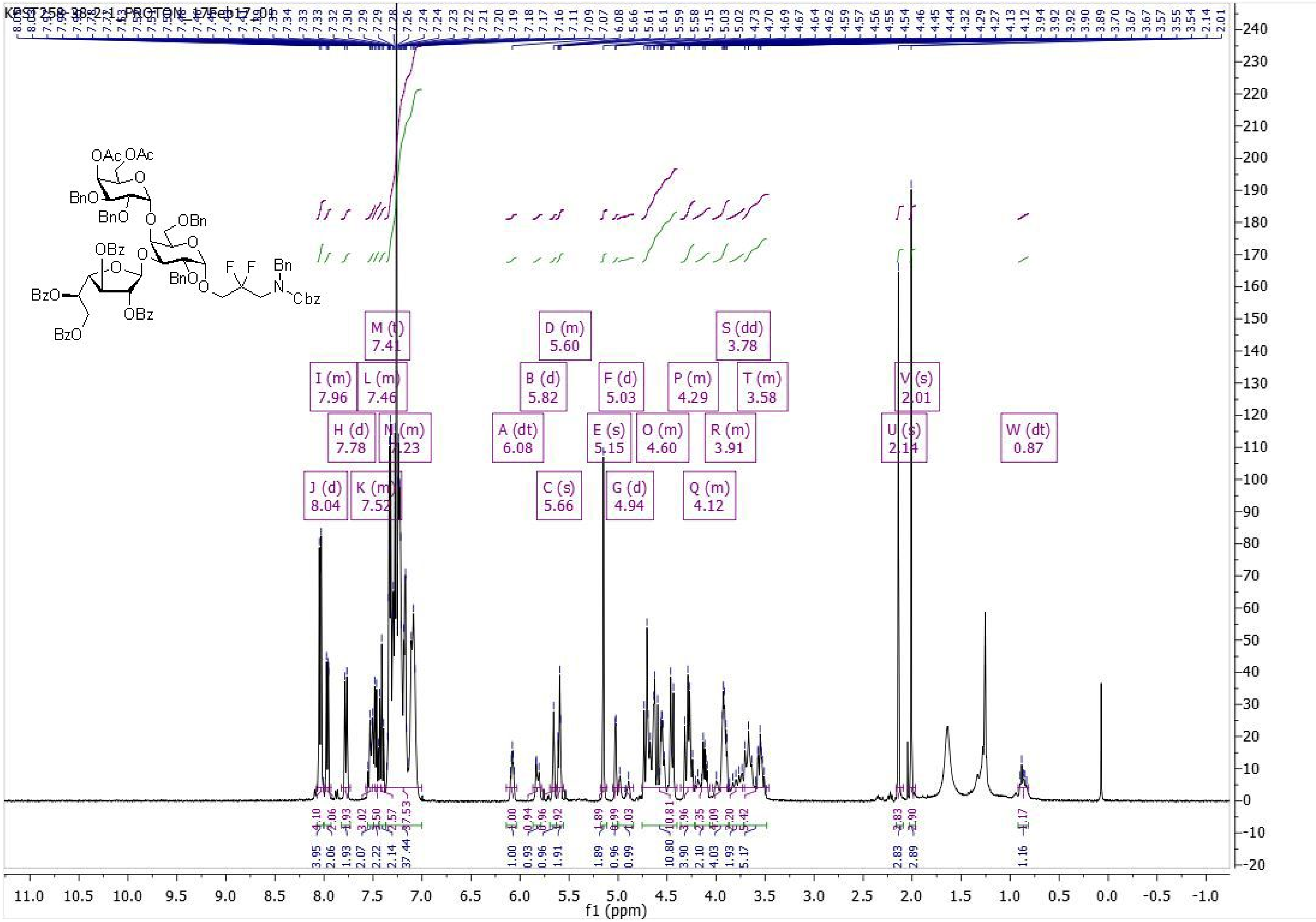

^{13}C-NMR of **3**

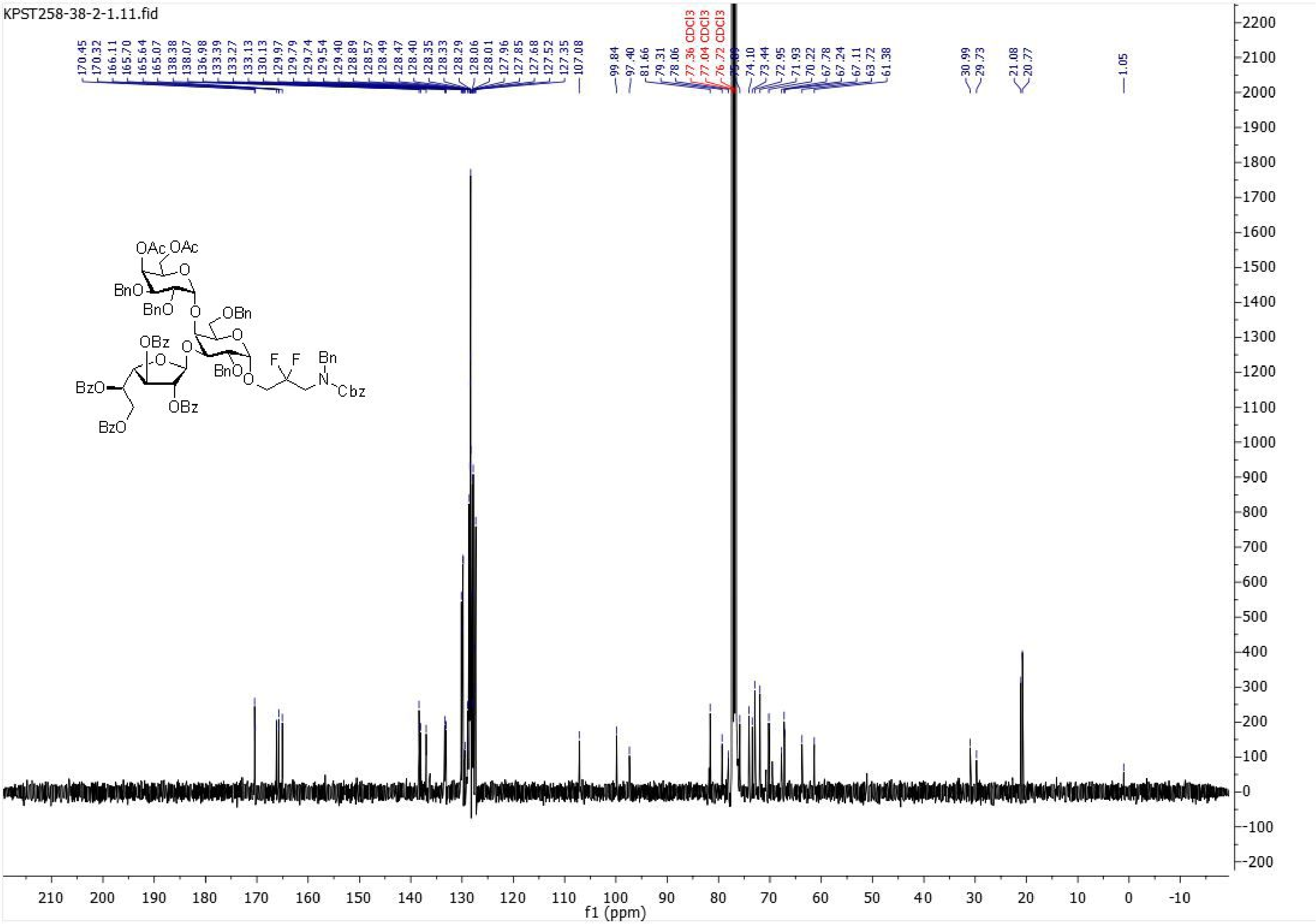
KPST258-38-2-1.11.fid
170.45
170.32
166.11
165.70
165.64
165.07
138.38
138.07
136.98
133.39
133.27
133.13
130.13
129.97
129.79
129.74
129.54
129.40
128.89
128.57
128.49
128.47
128.40
128.35
128.33
128.29
128.06
128.01
127.96
127.85
127.68
127.52
127.35
107.08
99.84
97.40
81.66
79.31
78.06
77.36 CDCl3
77.04 CDCl3
76.72 CDCl3
74.10
73.44
72.95
71.93
70.22
67.78
67.24
67.11
63.72
61.38
30.99
29.73
21.08
20.77
1.05
OAc
OAc
BnO
BnO
OBn
OBz
BzO
OBz
BzO
BnO
F
F
Bn
N
Cbz
f1 (ppm)

H-C HSQC of **3**

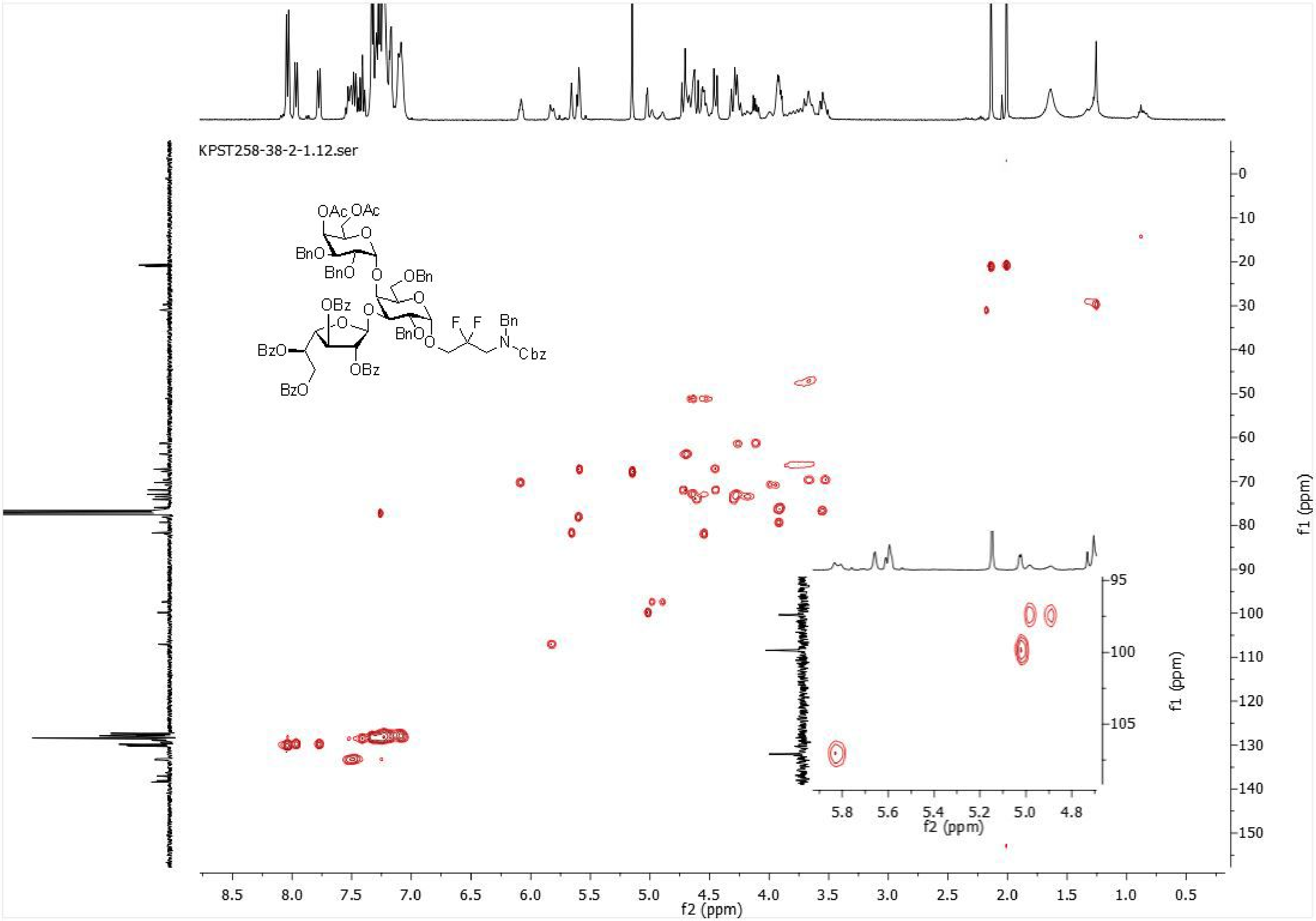

KPST258-38-2-1.12.ser
OAc OAc
BnO
BnO
OBn
OBz
BzO
OBz
BzO
BnO
F F
Bn
N
Cbz
f2 (ppm)
f1 (ppm)

^{1}H-NMR of **8**

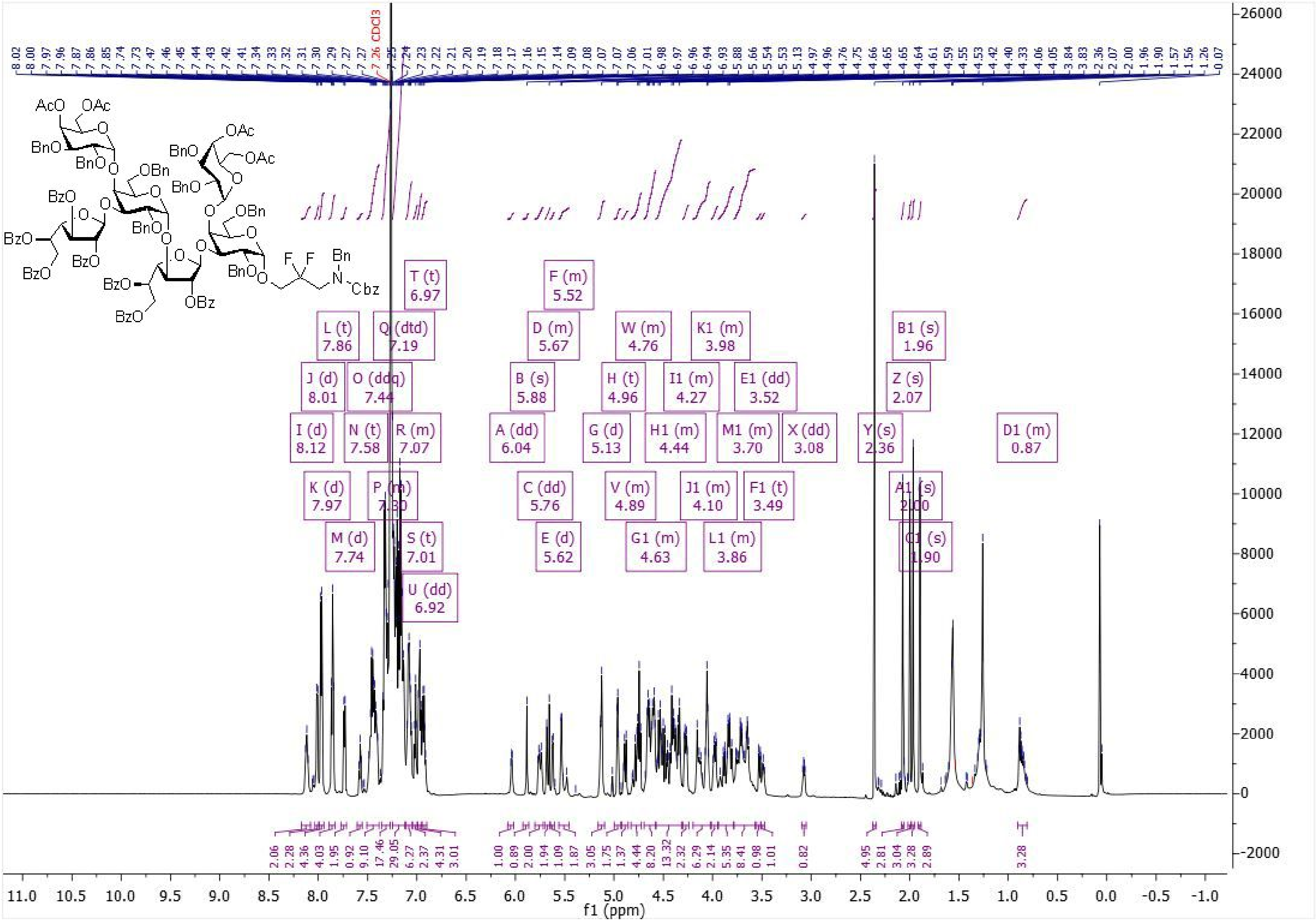

^{13}C-NMR of **8**

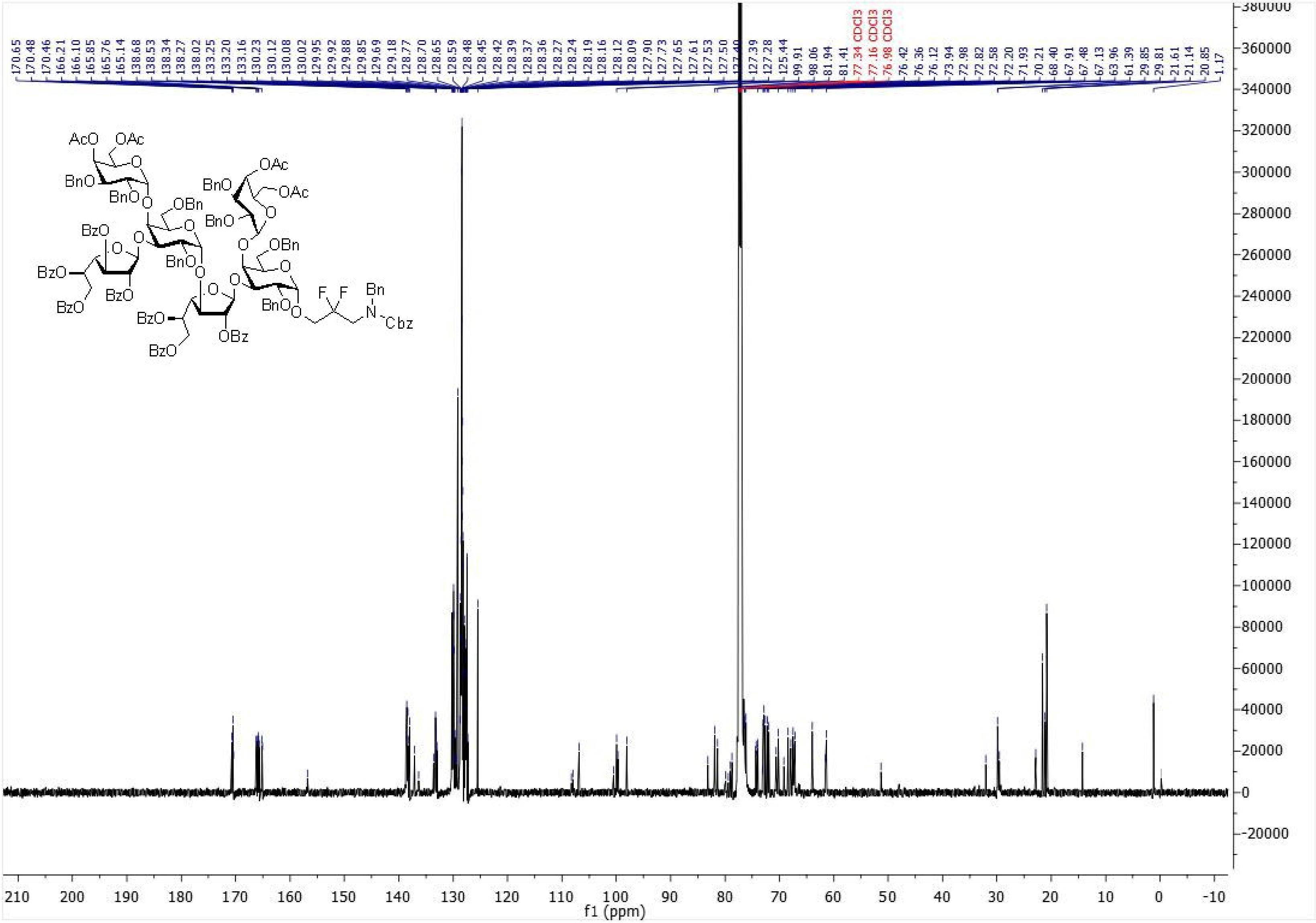

H-C HSQC of **8**

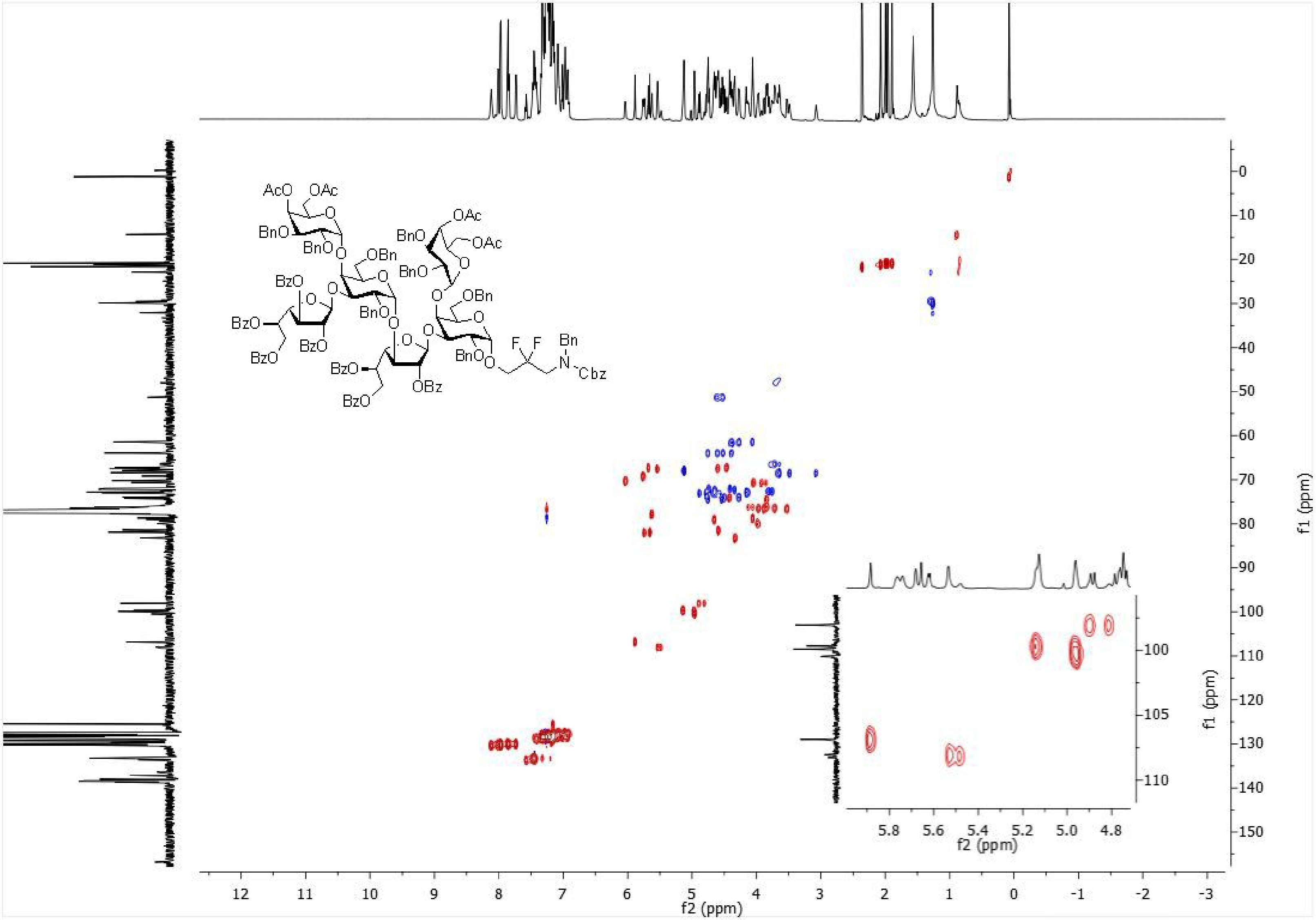

AcO
OAc
BnO
BnO
OBn
BnO
OAc
OAc
BnO
BzO
OBn
BzO
BnO
BzO
BzO
BnO
F F
Bn
N
Cbz
BzO
OBz
BzO
f1 (ppm)
f2 (ppm)

^{1}H-NMR of **9**

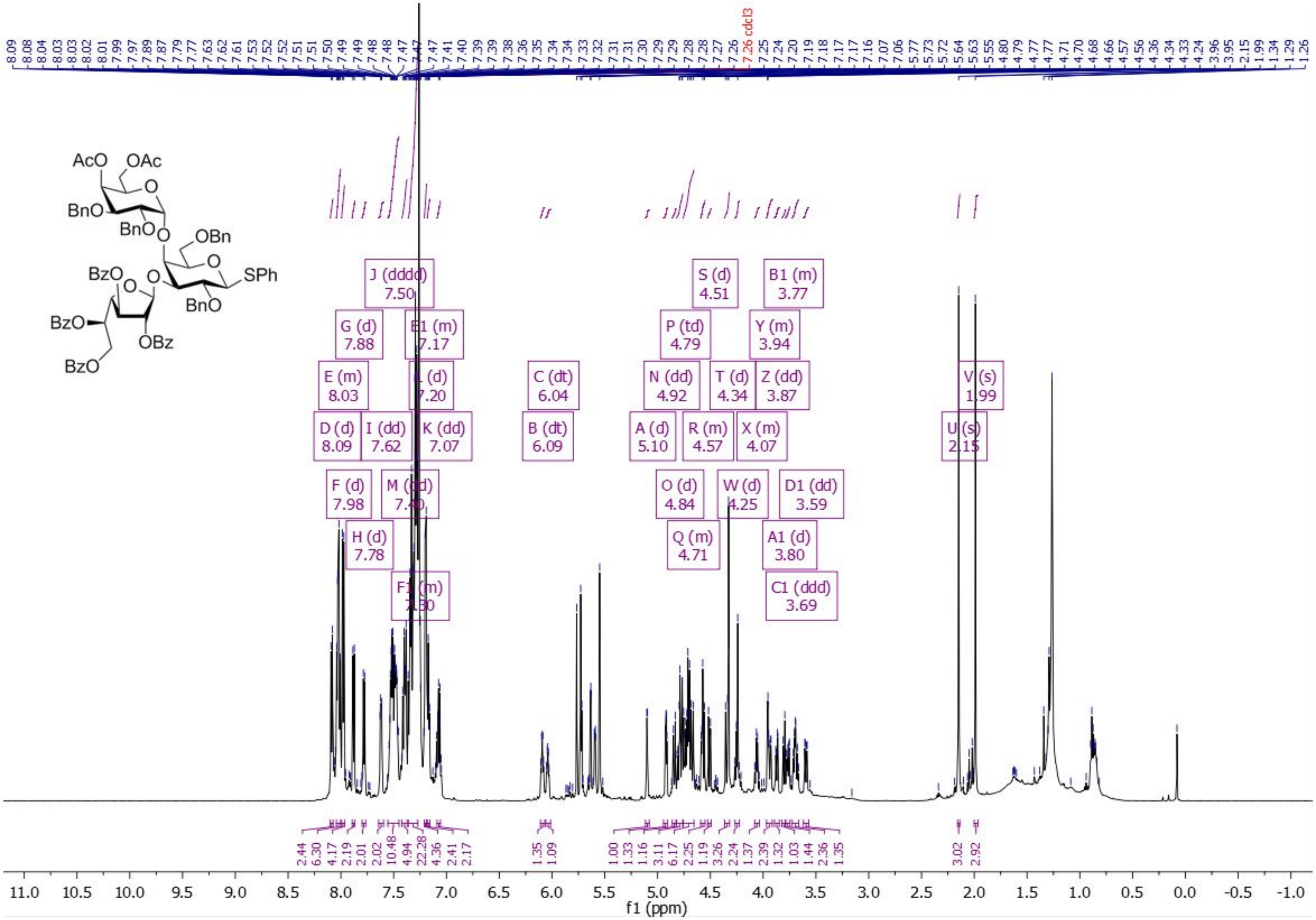

AcO
OAc
BnO
BnO
OBn
SPh
BzO
BnO
BzO
OBz
BzO
J (dddd) 7.50
G (d) 7.88
E1 (m) 7.17
E (m) 8.03
L (d) 7.20
D (d) 8.09
I (dd) 7.62
K (dd) 7.07
F (d) 7.98
M (dd) 7.40
H (d) 7.78
F1 (m) 7.30
C (dt) 6.04
B (dt) 6.09
S (d) 4.51
B1 (m) 3.77
P (td) 4.79
Y (m) 3.94
N (dd) 4.92
T (d) 4.34
Z (dd) 3.87
A (d) 5.10
R (m) 4.57
X (m) 4.07
O (d) 4.84
W (d) 4.25
D1 (dd) 3.59
Q (m) 4.71
A1 (d) 3.80
C1 (ddd) 3.69
V (s) 1.99
U (s) 2.15
7.26 cdcl3
f1 (ppm)

^{13}C-NMR of **9**

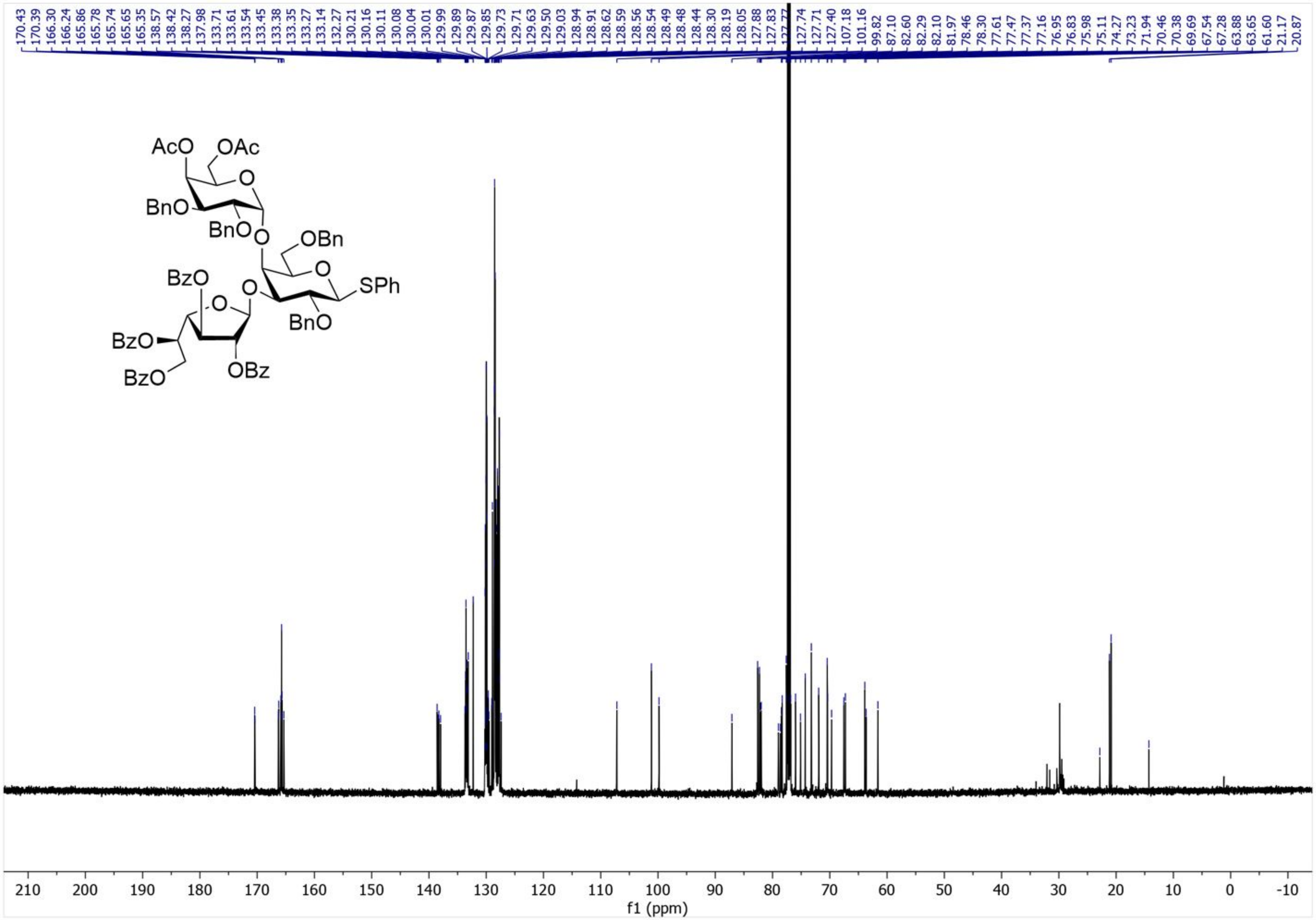
170.43
170.39
166.30
166.24
165.86
165.78
165.74
165.65
165.35
138.57
138.42
138.27
137.98
133.71
133.61
133.54
133.45
133.38
133.35
133.27
133.14
132.27
130.21
130.16
130.11
130.08
130.04
130.01
129.99
129.89
129.87
129.85
129.73
129.71
129.63
129.50
129.03
128.94
128.91
128.62
128.59
128.56
128.54
128.49
128.48
128.44
128.30
128.19
128.05
127.88
127.83
127.74
127.71
127.40
107.18
101.16
99.82
87.10
82.60
82.29
82.10
81.97
78.46
78.30
77.61
77.47
77.37
77.16
76.95
76.83
75.98
75.11
74.27
73.23
71.94
70.46
70.38
69.69
67.54
67.28
63.88
63.65
61.60
21.17
20.87
AcO
OAc
BnO
BnO
O
OBn
BzO
SPh
BzO
OBz
BnO
BzO
210
200
190
180
170
160
150
140
130
120
110
100
90
80
70
60
50
40
30
20
10
0
-10
f1 (ppm)

H-C HSQC of **9**

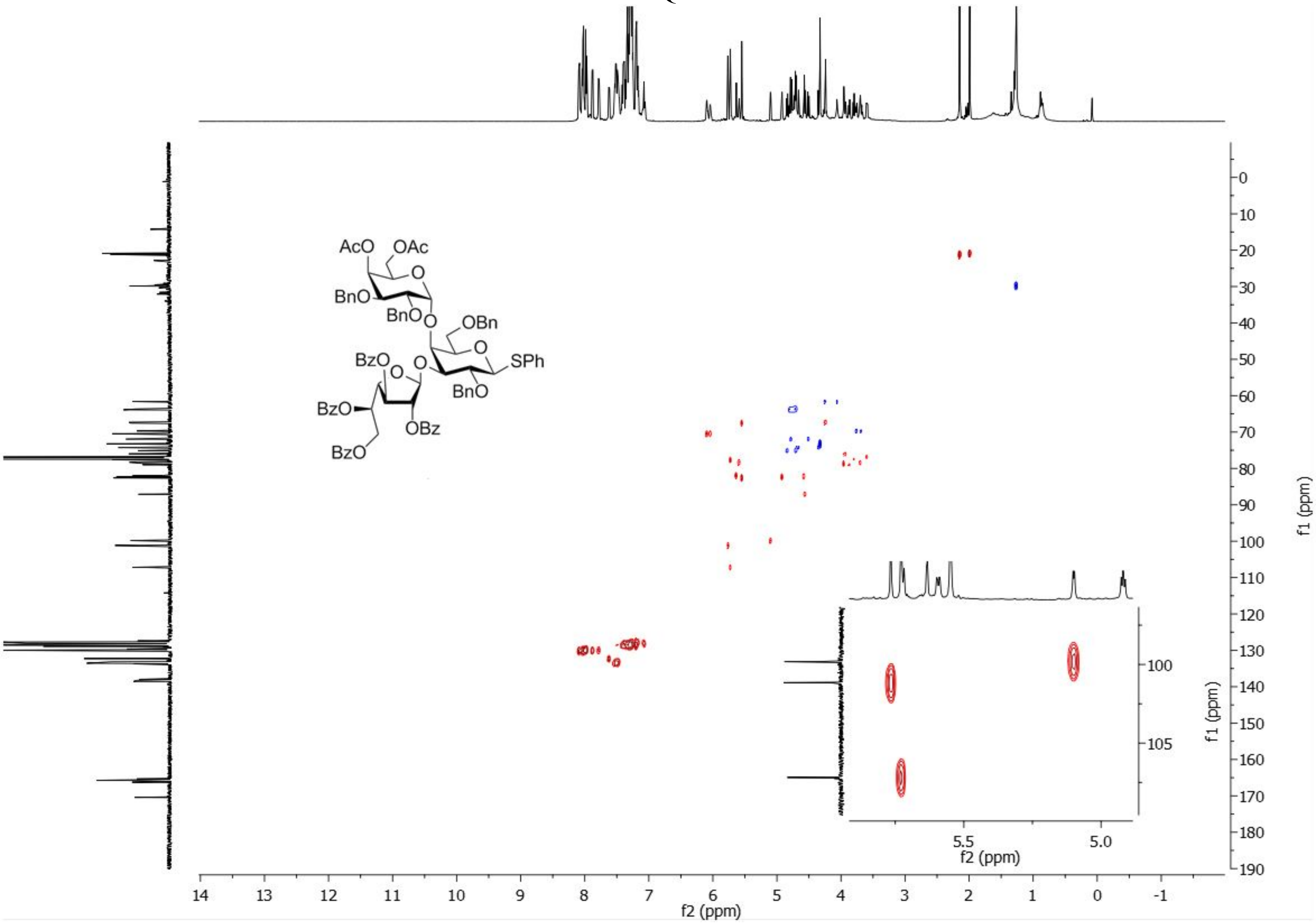

^{1}H-NMR of **23**

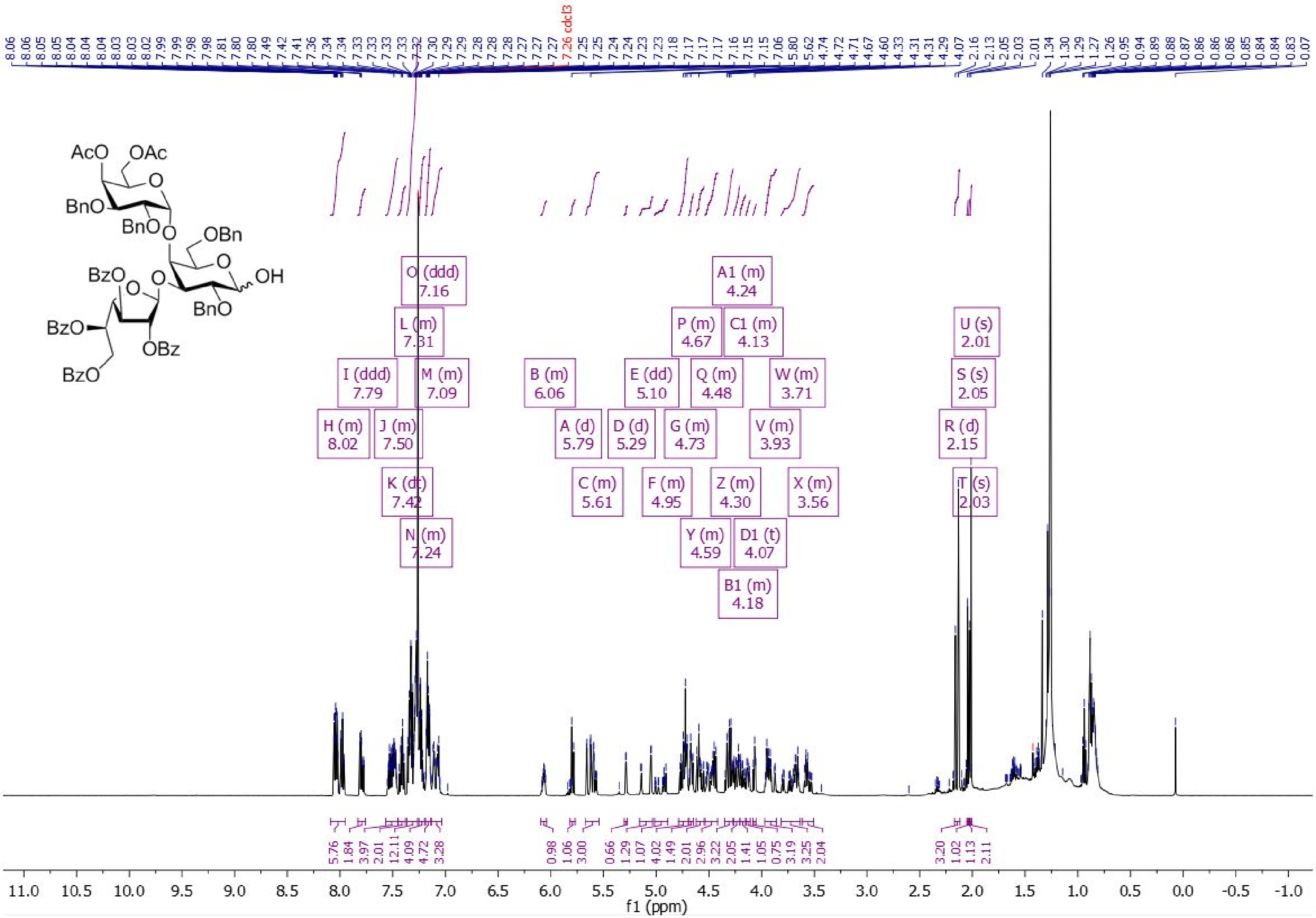

^{13}C-NMR of **23**

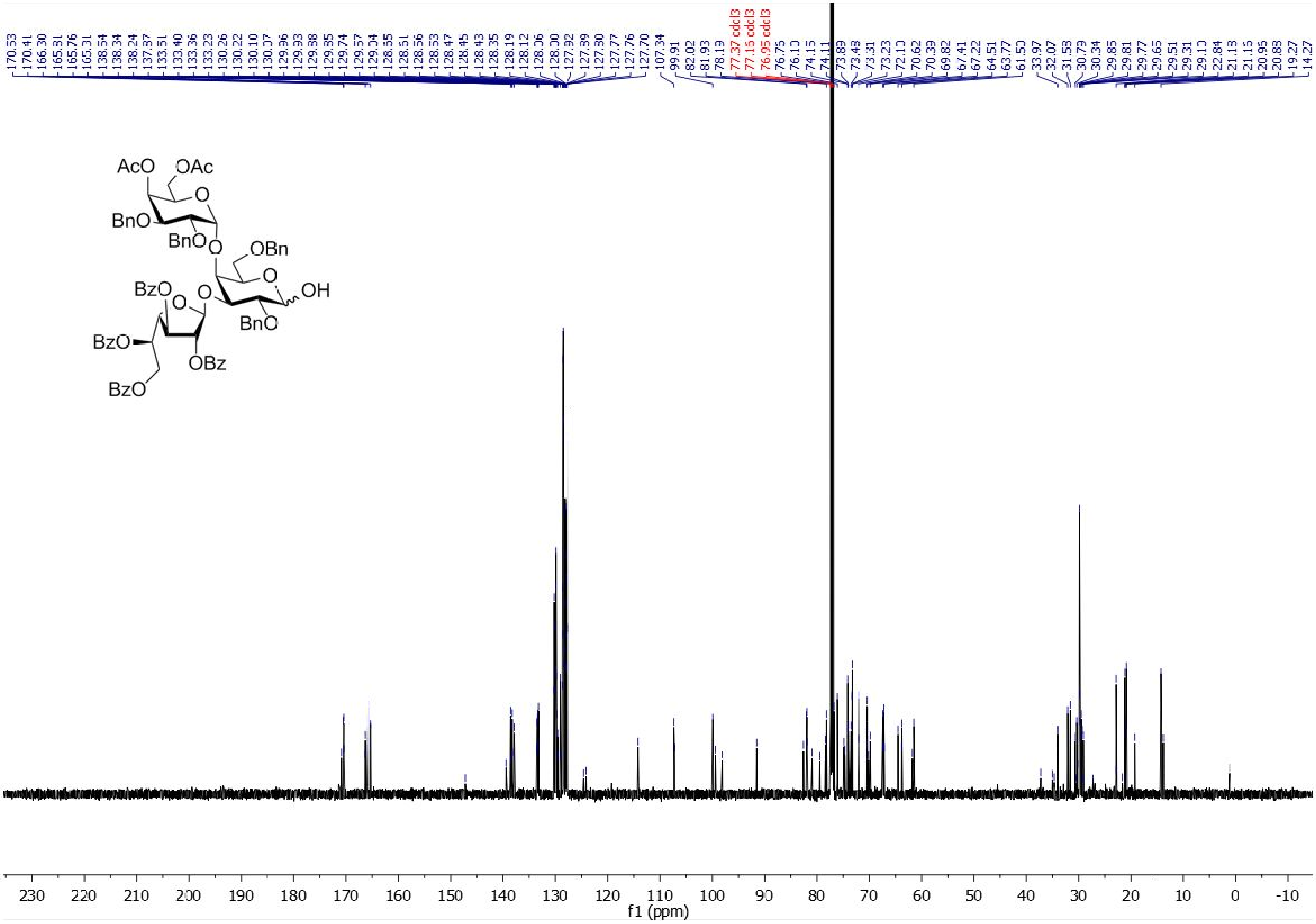

170.53
170.41
166.30
165.81
165.76
165.31
138.54
138.34
138.24
137.87
133.51
133.40
133.36
133.23
130.26
130.22
130.10
130.07
129.96
129.93
129.88
129.85
129.74
129.57
129.04
128.65
128.61
128.56
128.53
128.47
128.45
128.43
128.35
128.19
128.12
128.06
128.00
127.92
127.89
127.80
127.77
127.76
127.70
107.34
99.91
82.02
81.93
78.19
77.37 cdcl3
77.16 cdcl3
76.95 cdcl3
76.76
76.10
74.15
74.11
73.89
73.48
73.31
73.23
72.10
70.62
70.39
69.82
67.41
67.22
64.51
63.77
61.50
33.97
32.07
31.58
30.79
30.34
29.85
29.81
29.77
29.65
29.51
29.31
29.10
22.84
21.18
21.16
20.96
20.88
19.27
14.27
AcO
OAc
O
BnO
BnO
O
OBn
O
OH
BzO
O
O
BnO
BzO
OBz
BzO
230 220 210 200 190 180 170 160 150 140 130 120 110 100 90 80 70 60 50 40 30 20 10 0 -10
f1 (ppm)

H-C HSQC of **23**

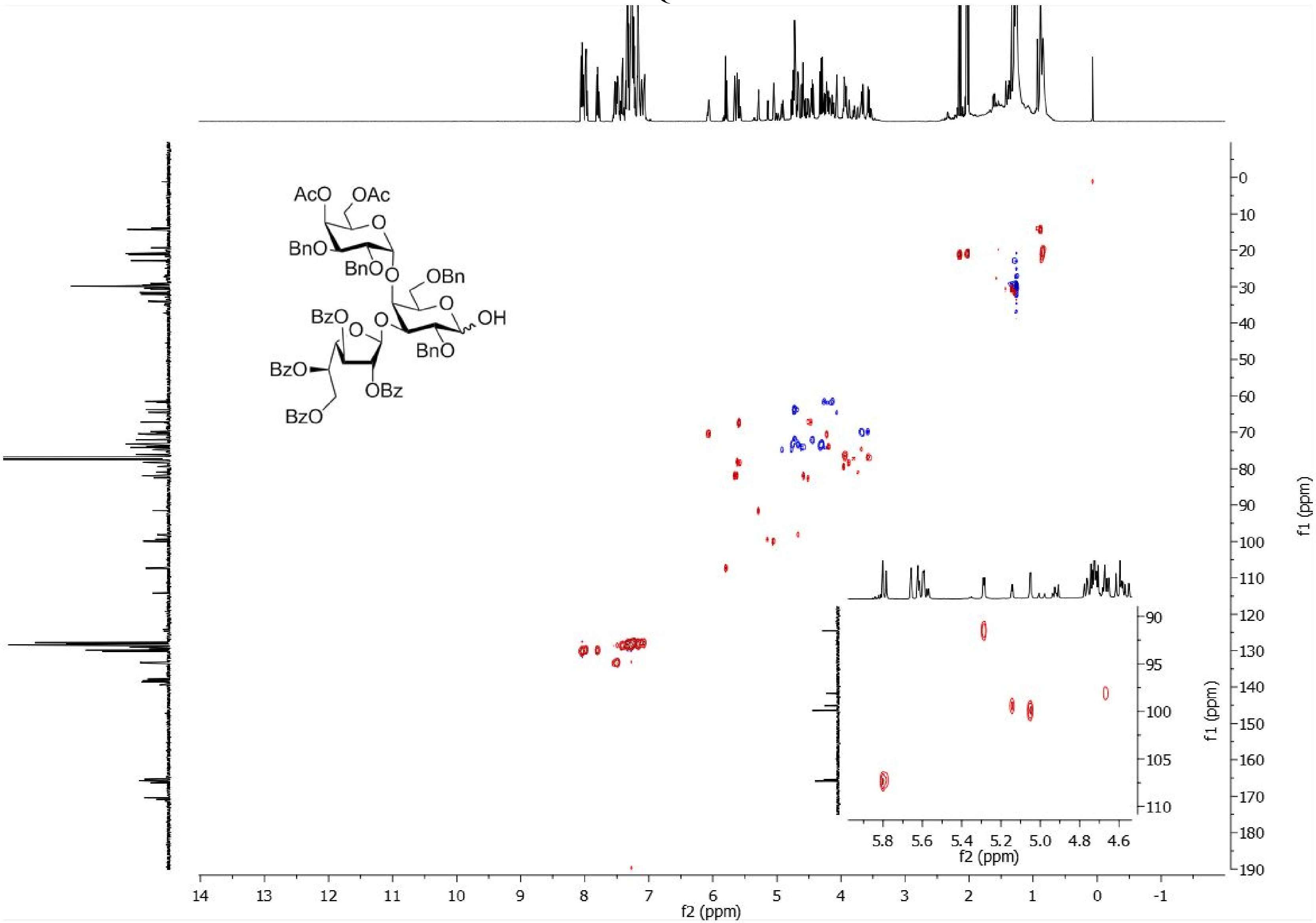

Coupled H-C HSQC of **23**

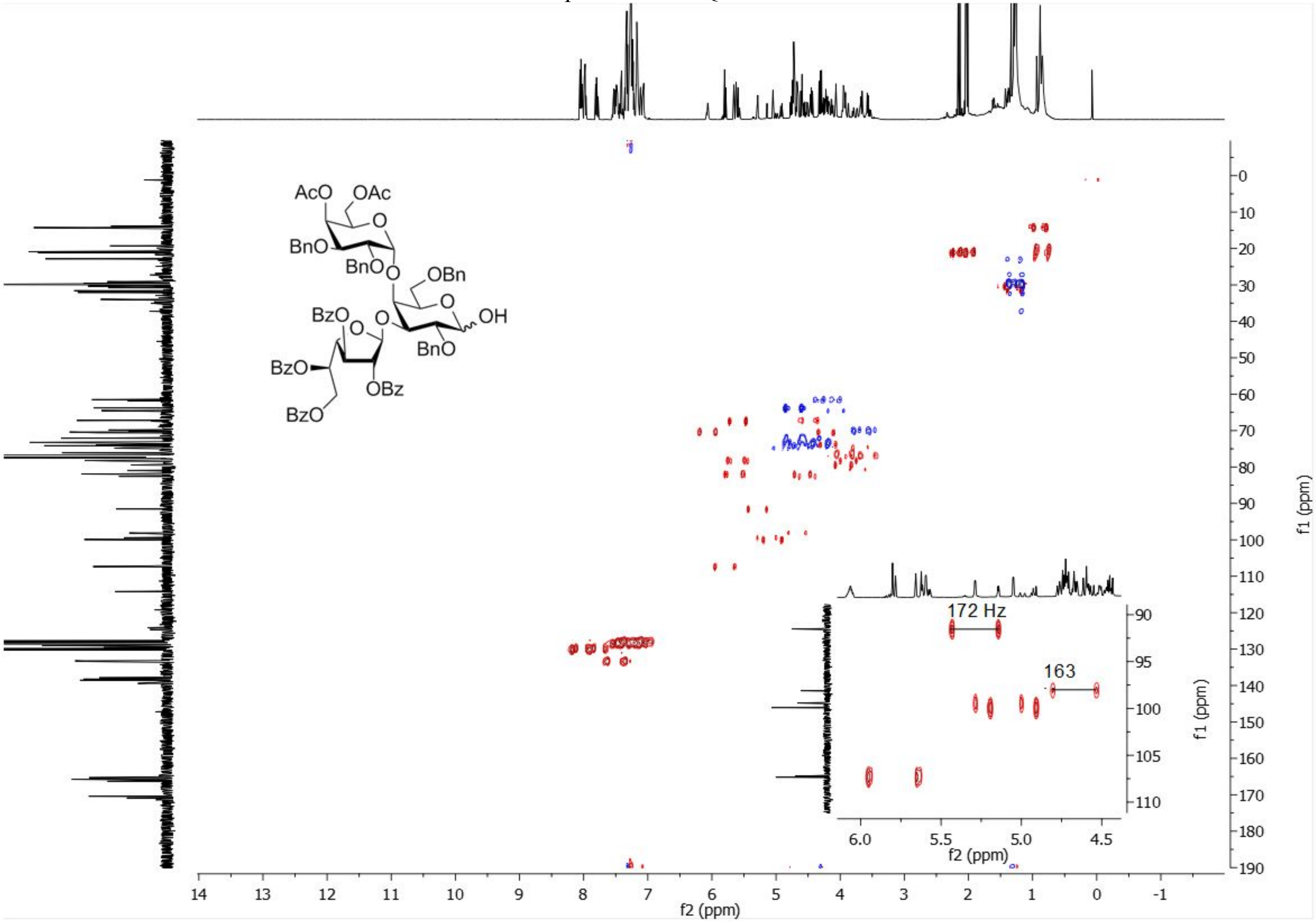

^{1}H-NMR of **24**

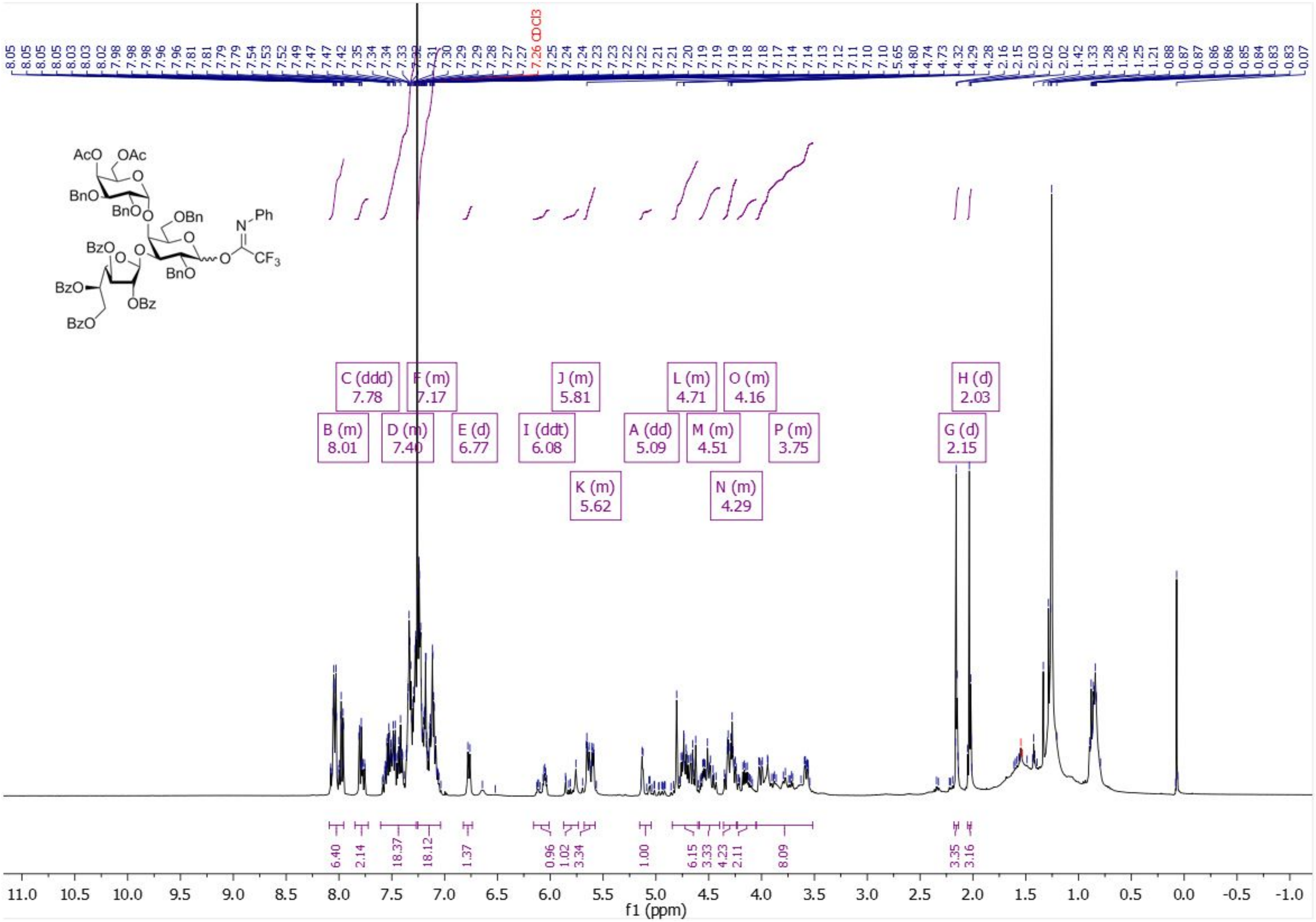

^{13}C-NMR of **24**

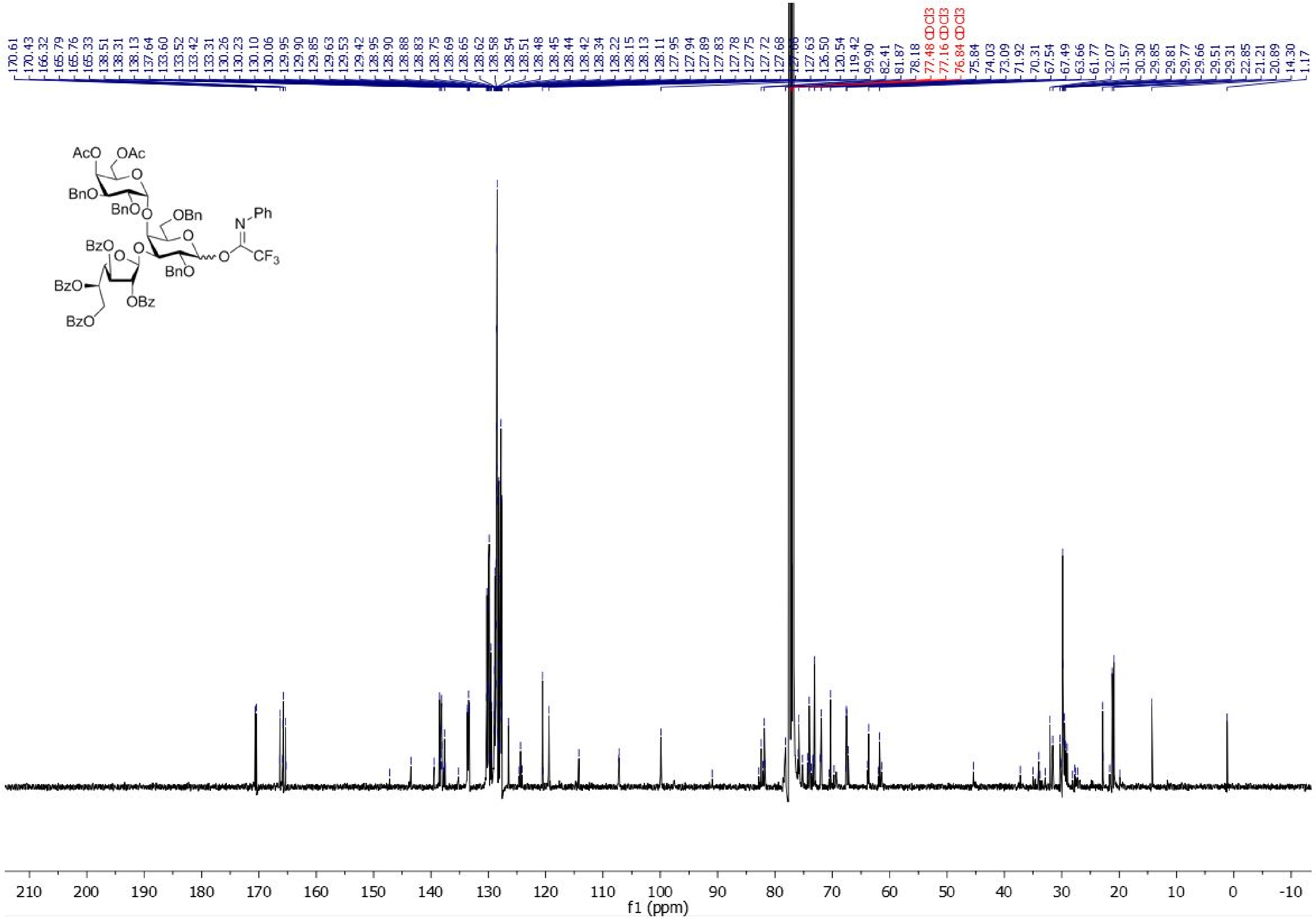

H-C HSQC of **24**

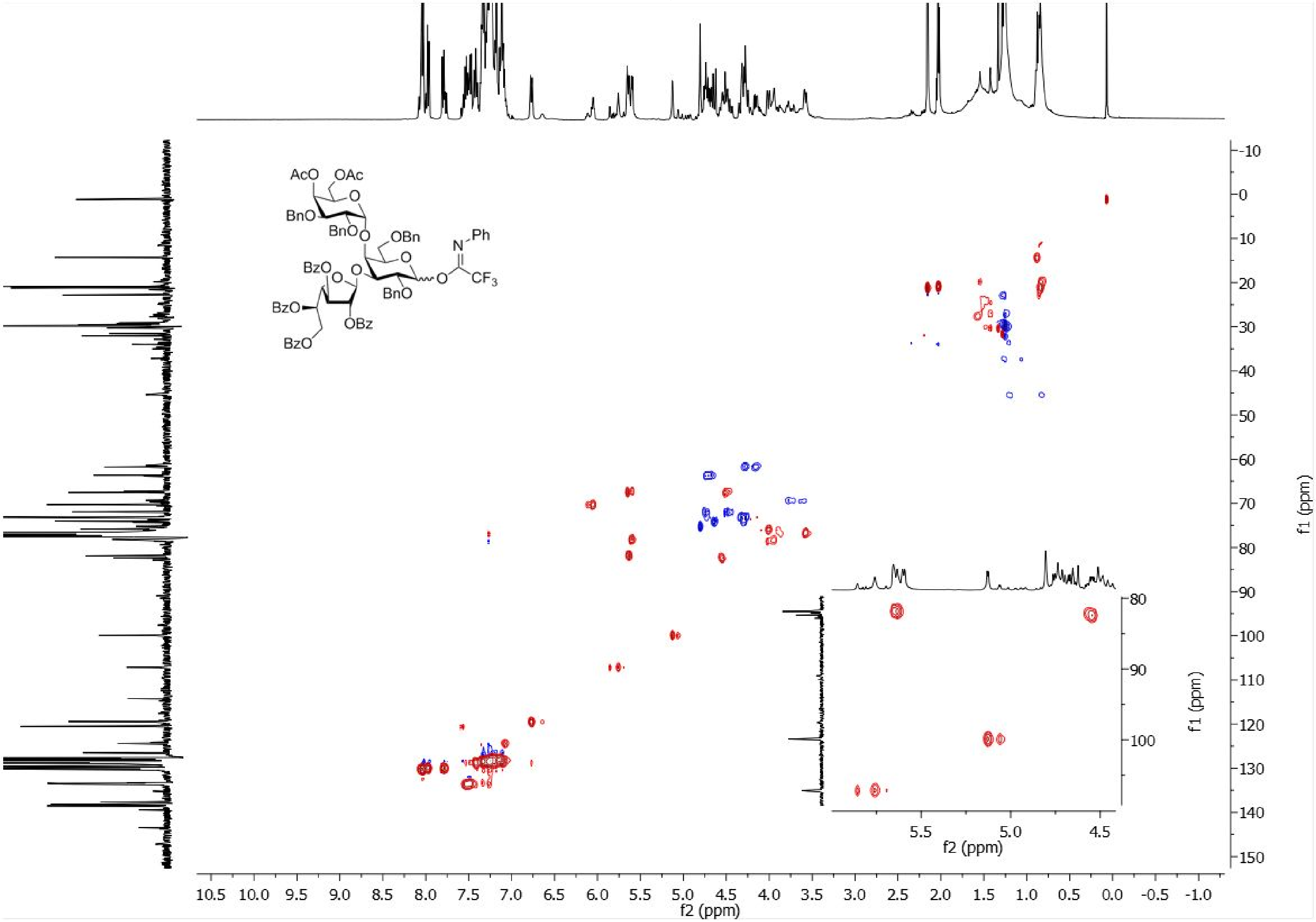

AcO
OAc
BnO
BnO
O
OBn
N
Ph
BzO
O
O
O
CF3
BnO
BzO
OBz
BzO
f1 (ppm)
f2 (ppm)

^{1}H-NMR of **10**

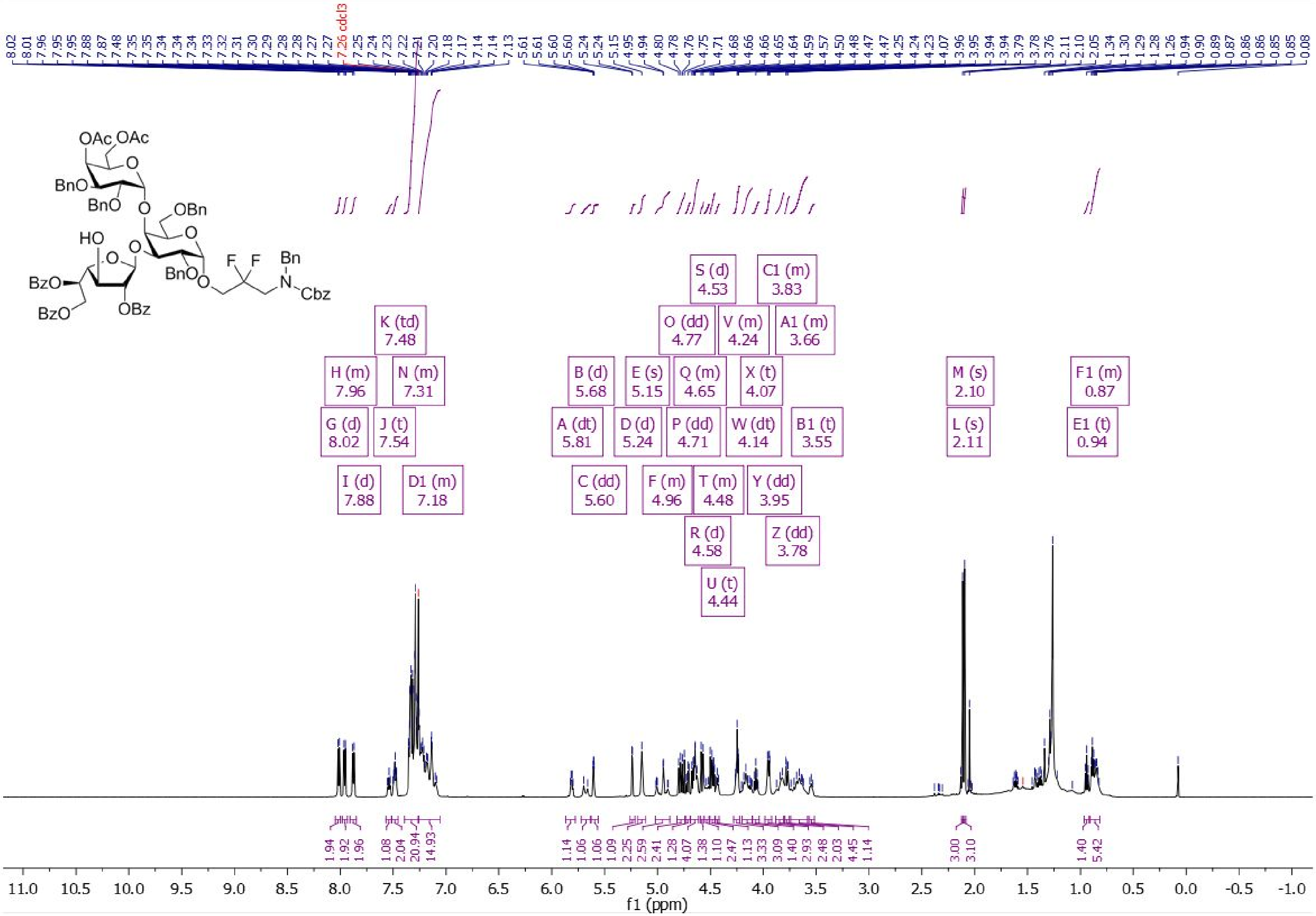

^{13}C-NMR of **10**

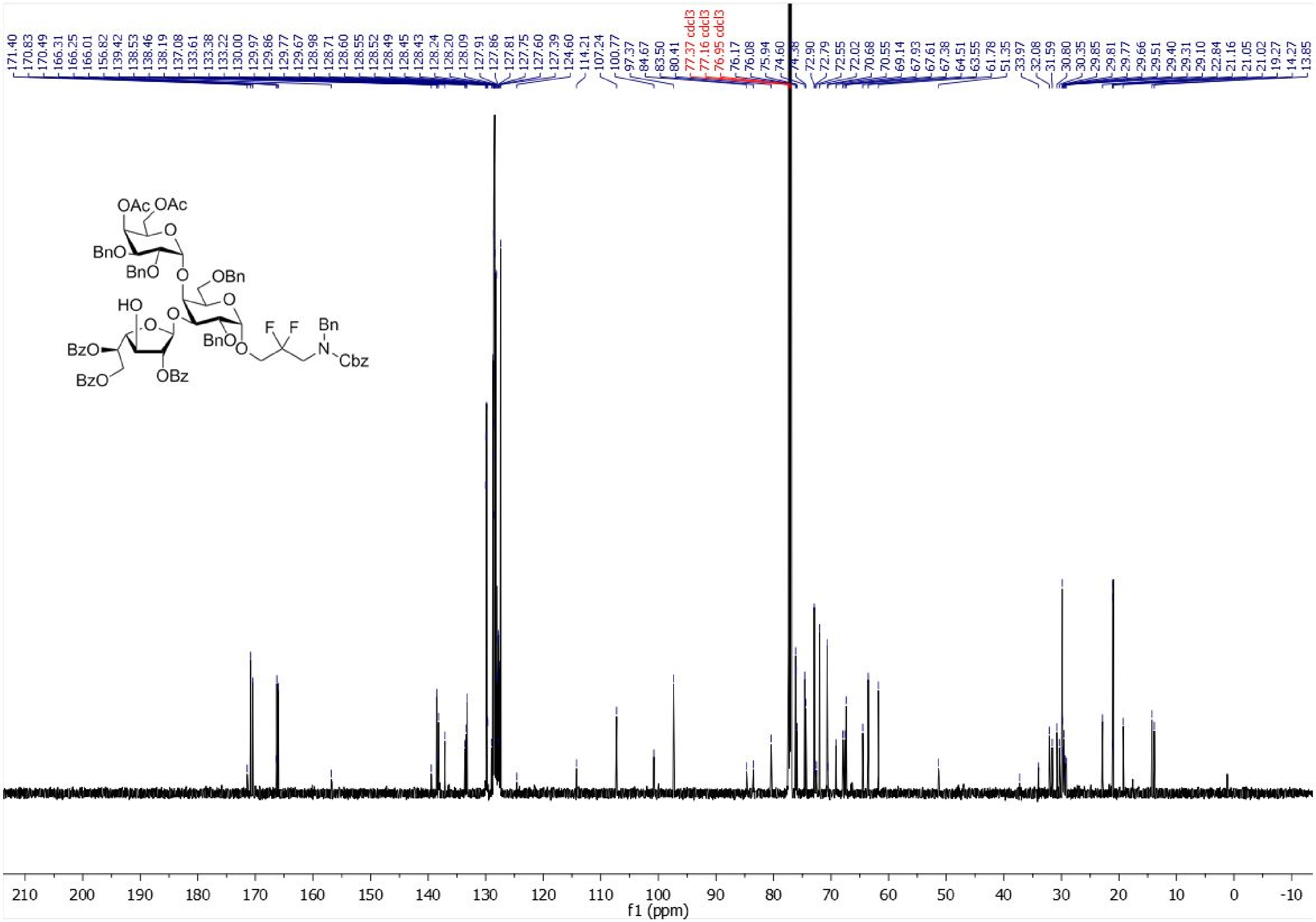

H-C HSQC of **10**

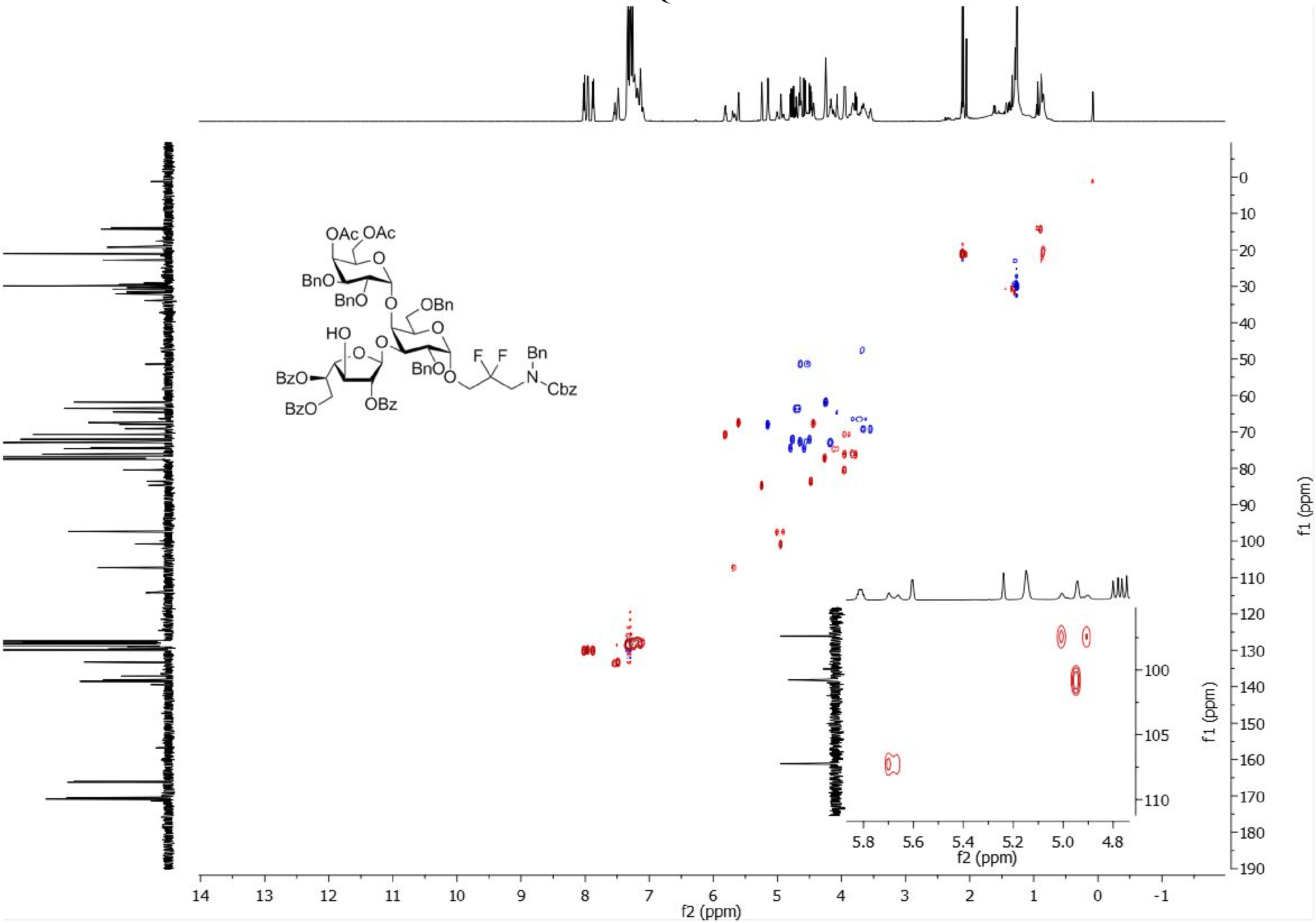

Coupled H-C HSQC of **10**

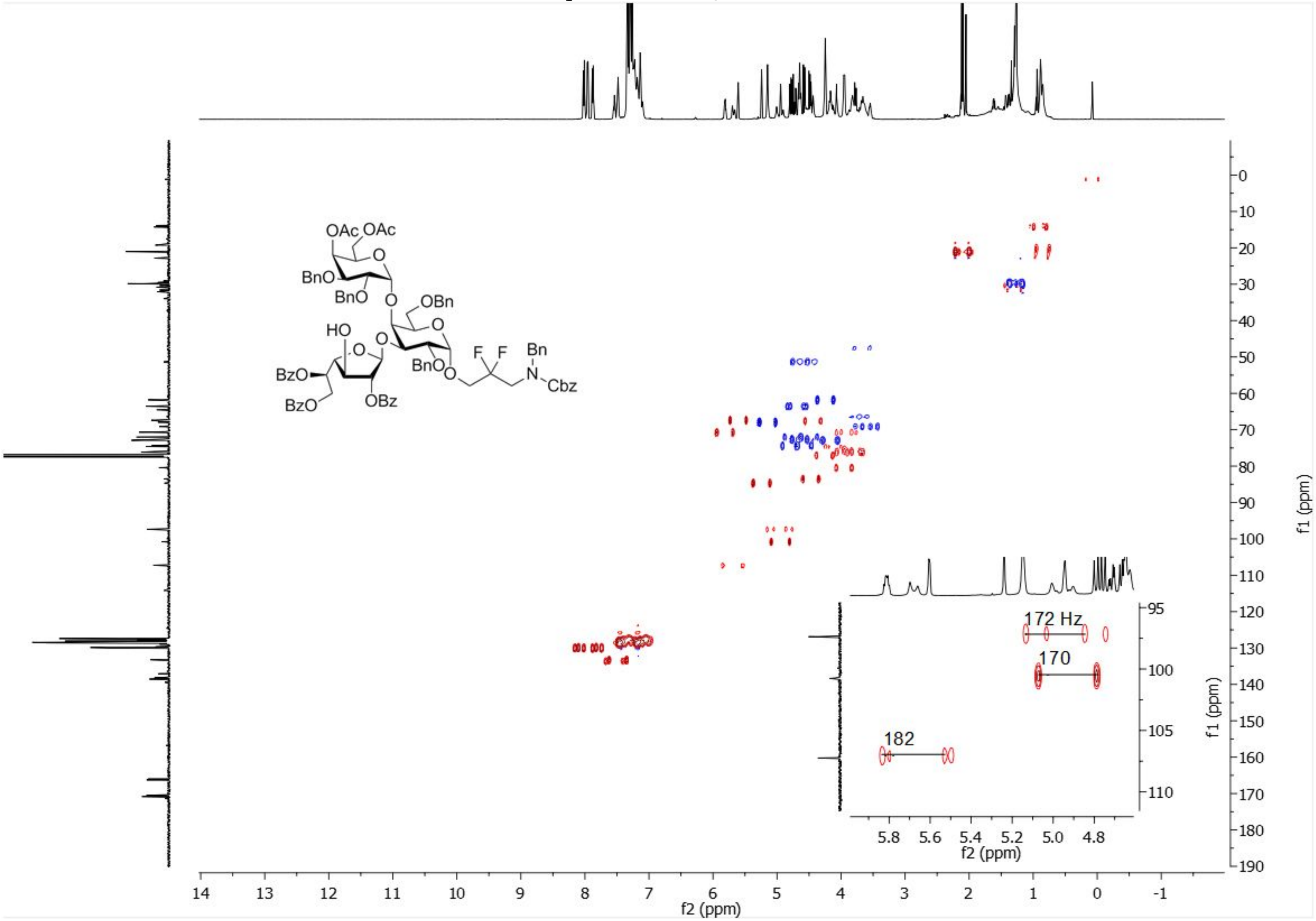

^{1}H-NMR of **11**

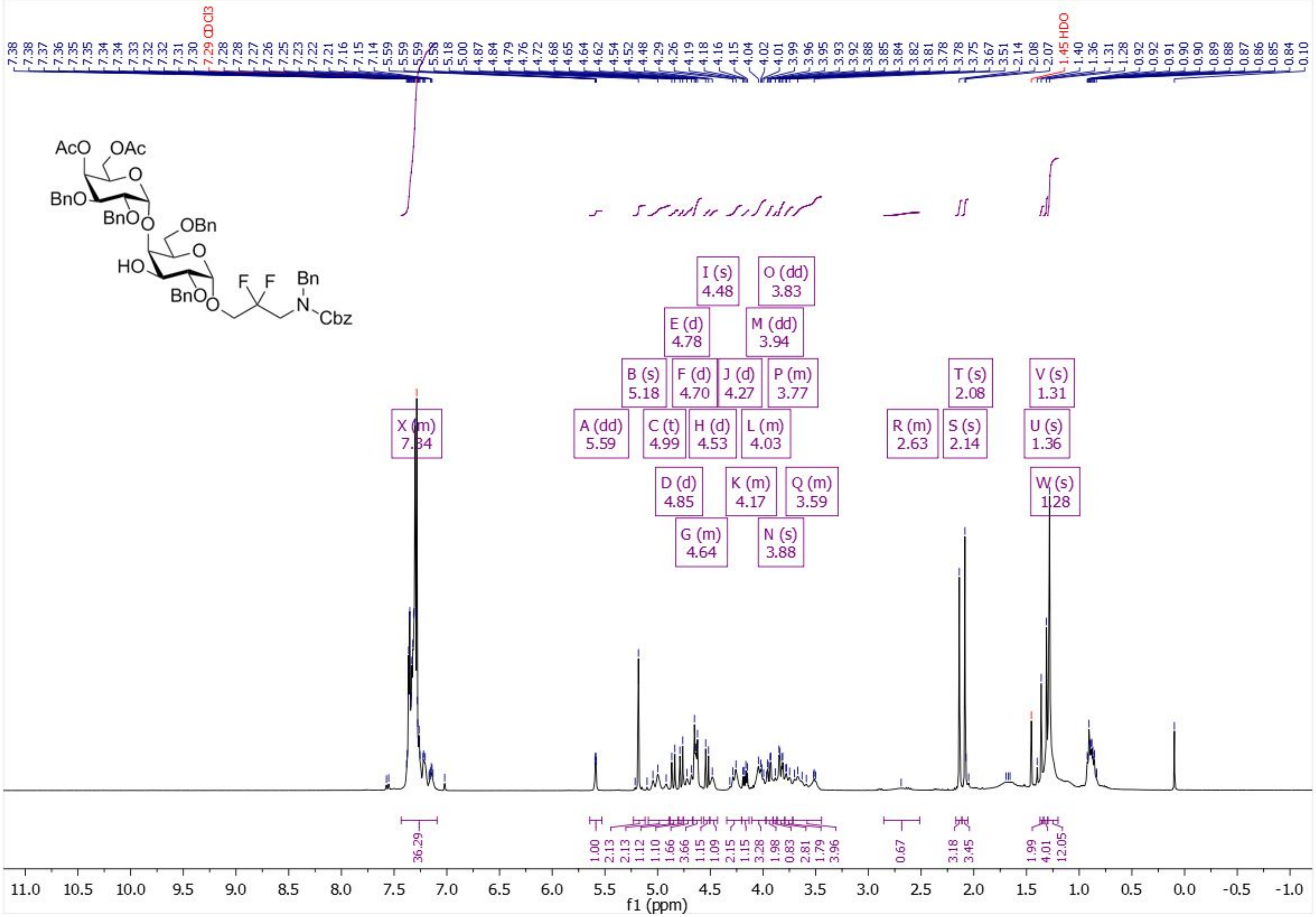

AcO OAc
BnO
BnO
OBn
HO
BnO
F F
Bn
N
Cbz
X (m) 7.34
A (dd) 5.59
B (s) 5.18
C (t) 4.99
D (d) 4.85
E (d) 4.78
F (d) 4.70
G (m) 4.64
H (d) 4.53
I (s) 4.48
J (d) 4.27
K (m) 4.17
L (m) 4.03
M (dd) 3.94
N (s) 3.88
O (dd) 3.83
P (m) 3.77
Q (m) 3.59
R (m) 2.63
S (s) 2.14
T (s) 2.08
U (s) 1.36
V (s) 1.31
W (s) 1.28
CDCl3
HDO
f1 (ppm)

^{13}C-NMR of **11**

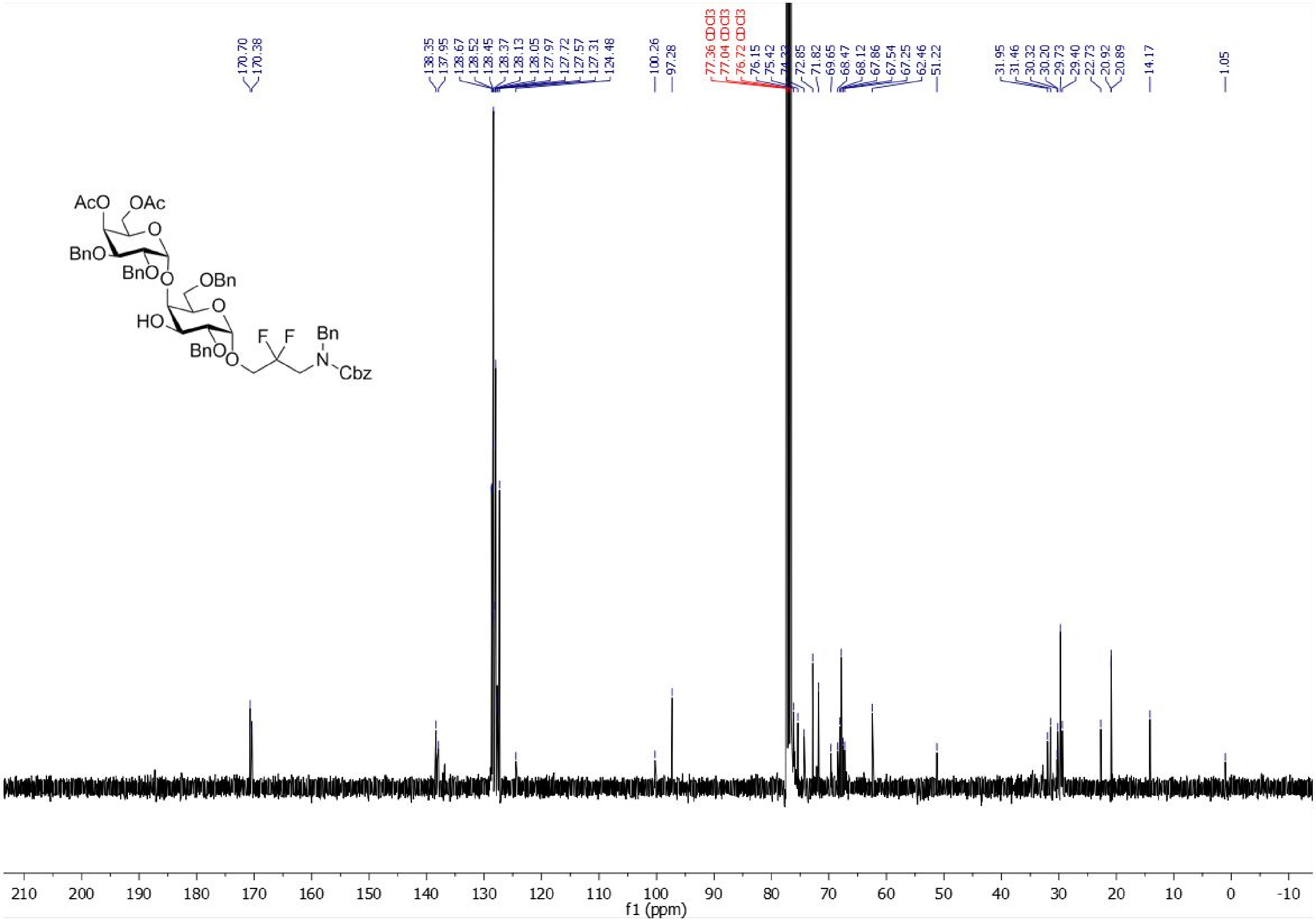
170.70
170.38
138.35
137.95
128.67
128.52
128.45
128.37
128.13
128.05
127.97
127.72
127.57
127.31
124.48
100.26
97.28
77.36 CDCl3
77.04 CDCl3
76.72 CDCl3
76.15
75.42
72.85
71.82
69.65
68.47
68.12
67.86
67.54
67.25
62.46
51.22
31.95
31.46
30.32
30.20
29.73
29.40
22.73
20.92
20.89
14.17
1.05
AcO
OAc
BnO
BnO
OBn
HO
BnO
F
F
Bn
N
Cbz
210 200 190 180 170 160 150 140 130 120 110 100 90 80 70 60 50 40 30 20 10 0 -10
f1 (ppm)

H-C HSQC of **11**

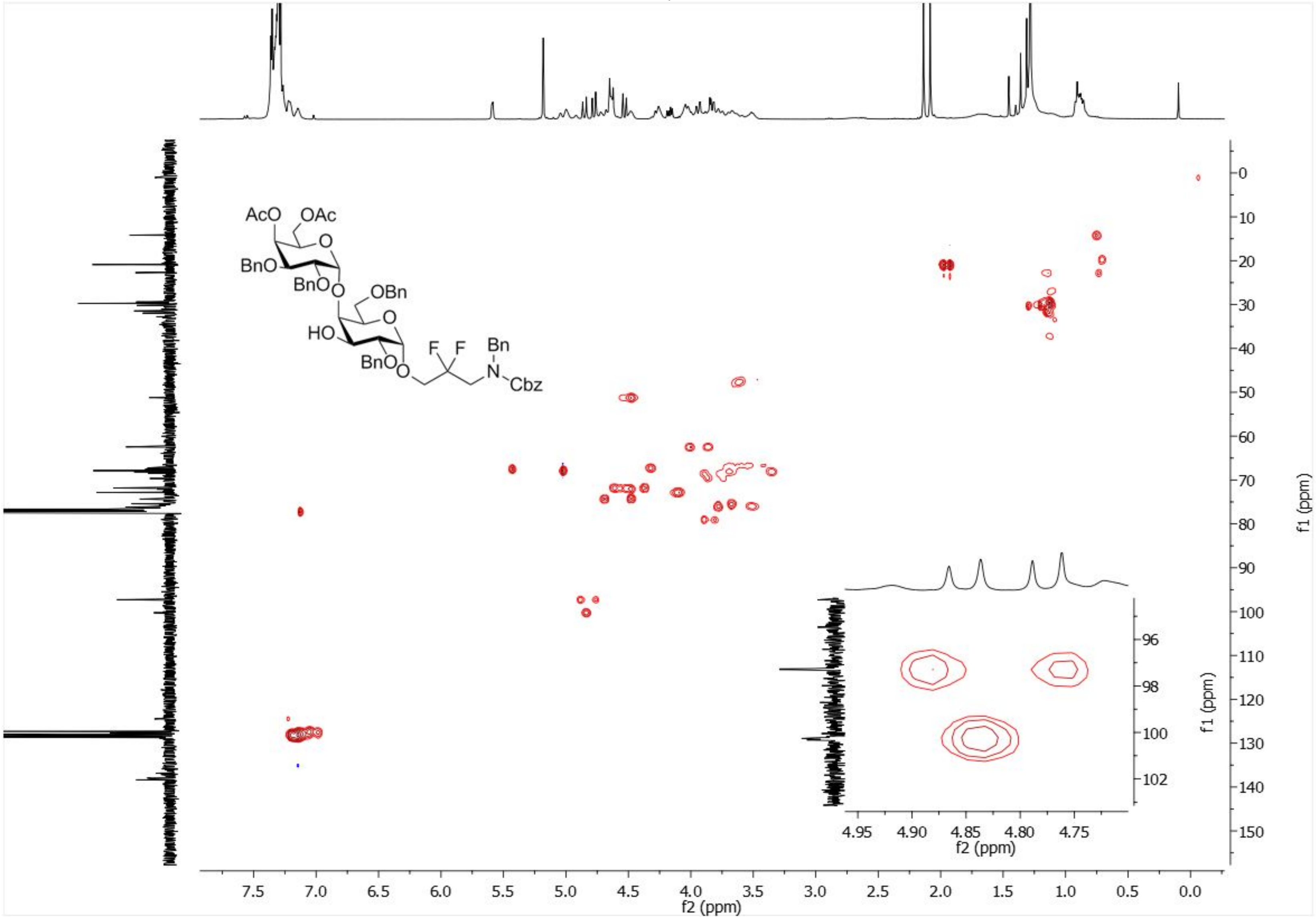

^{1}H-NMR of **16**

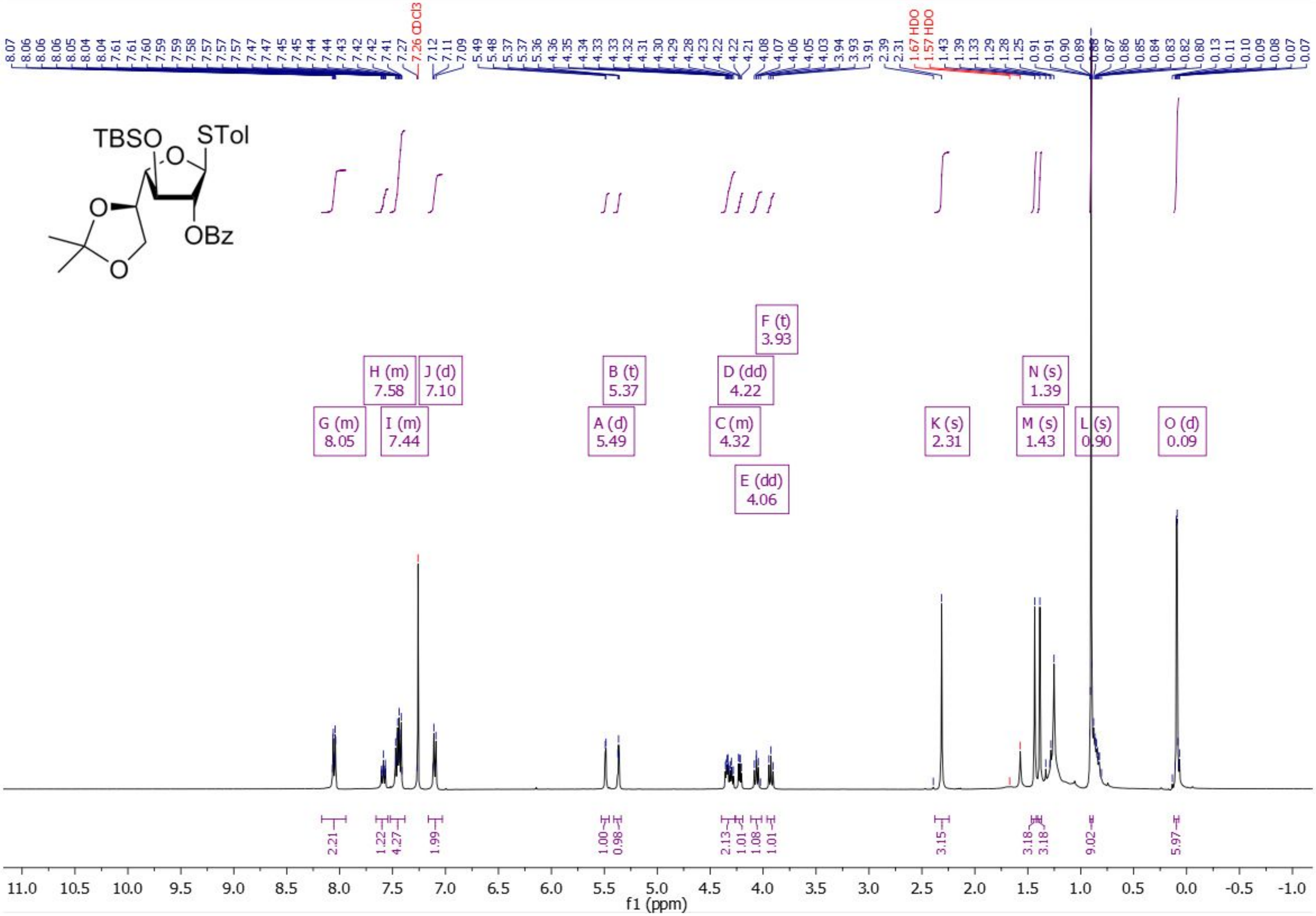

^{13}C-NMR of **16**

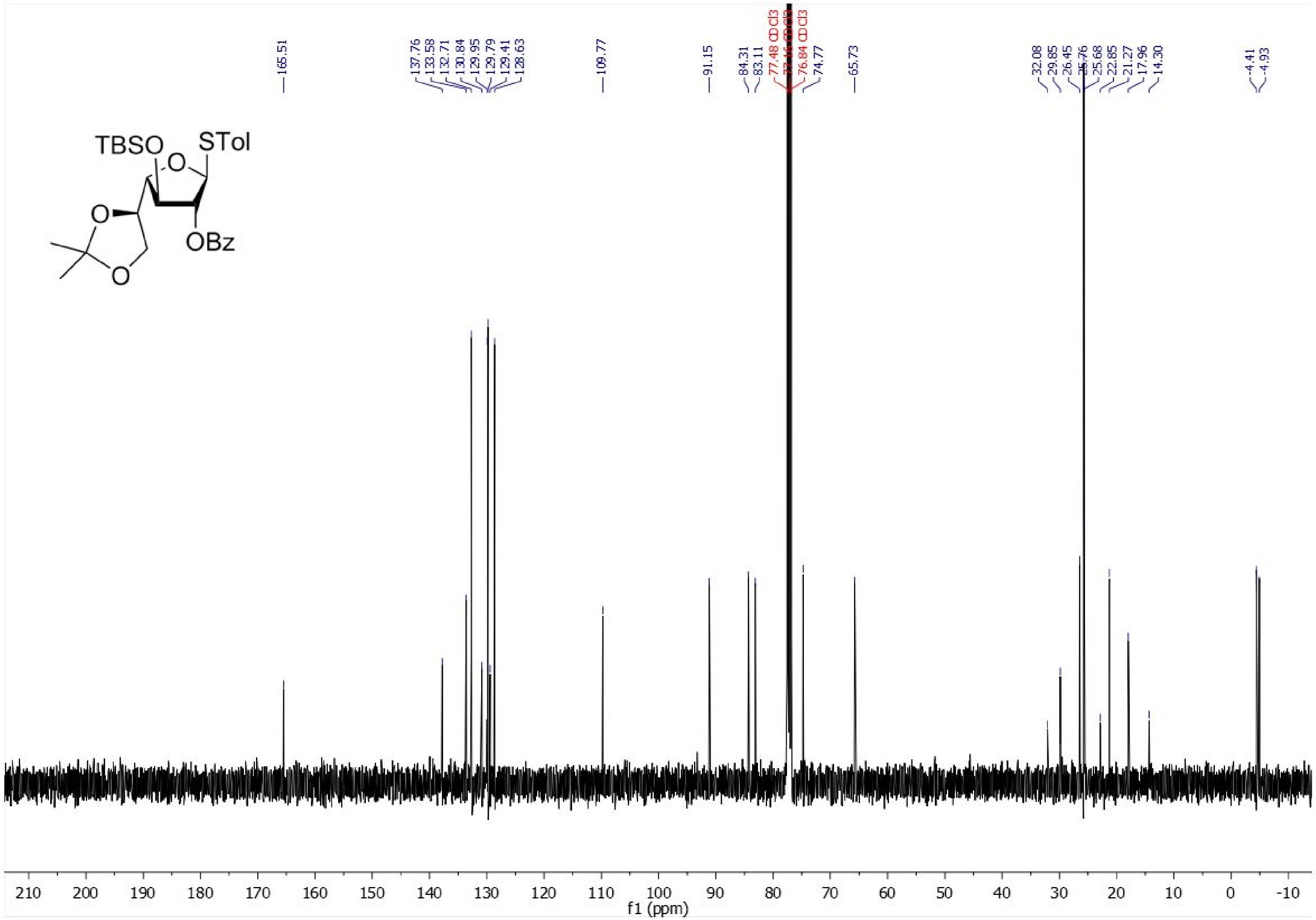
TBSO
STol
O
O
O
OBz
165.51
137.76
133.58
132.71
130.84
129.95
129.79
129.41
128.63
109.77
91.15
84.31
83.11
77.48 CDCl3
76.84 CDCl3
74.77
65.73
32.08
29.85
26.45
25.76
25.68
22.85
21.27
17.96
14.30
-4.41
-4.93
210
200
190
180
170
160
150
140
130
120
110
100
90
80
70
60
50
40
30
20
10
0
-10
f1 (ppm)

H-C HSQC of **16**

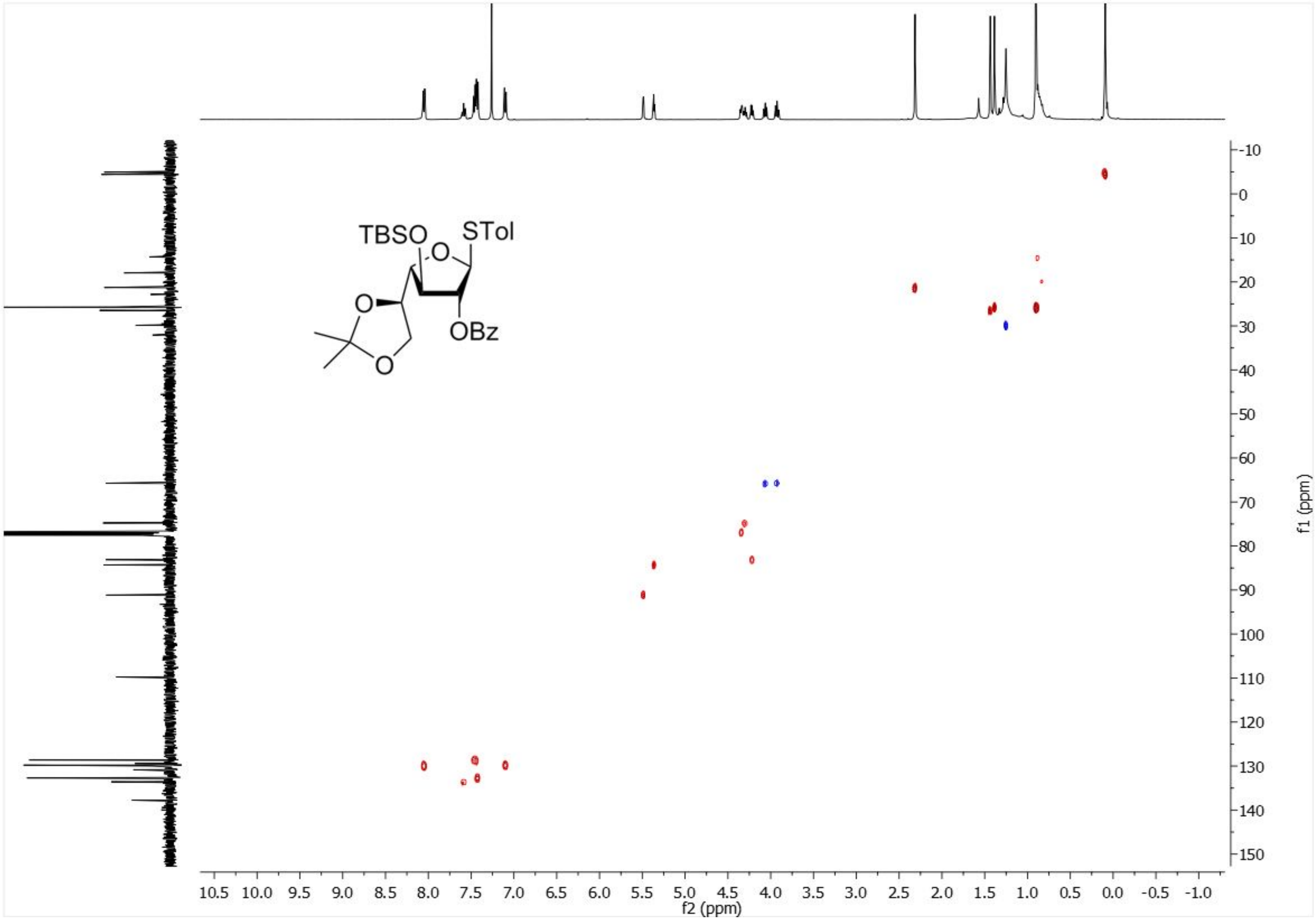

^{1}H-NMR of **18**

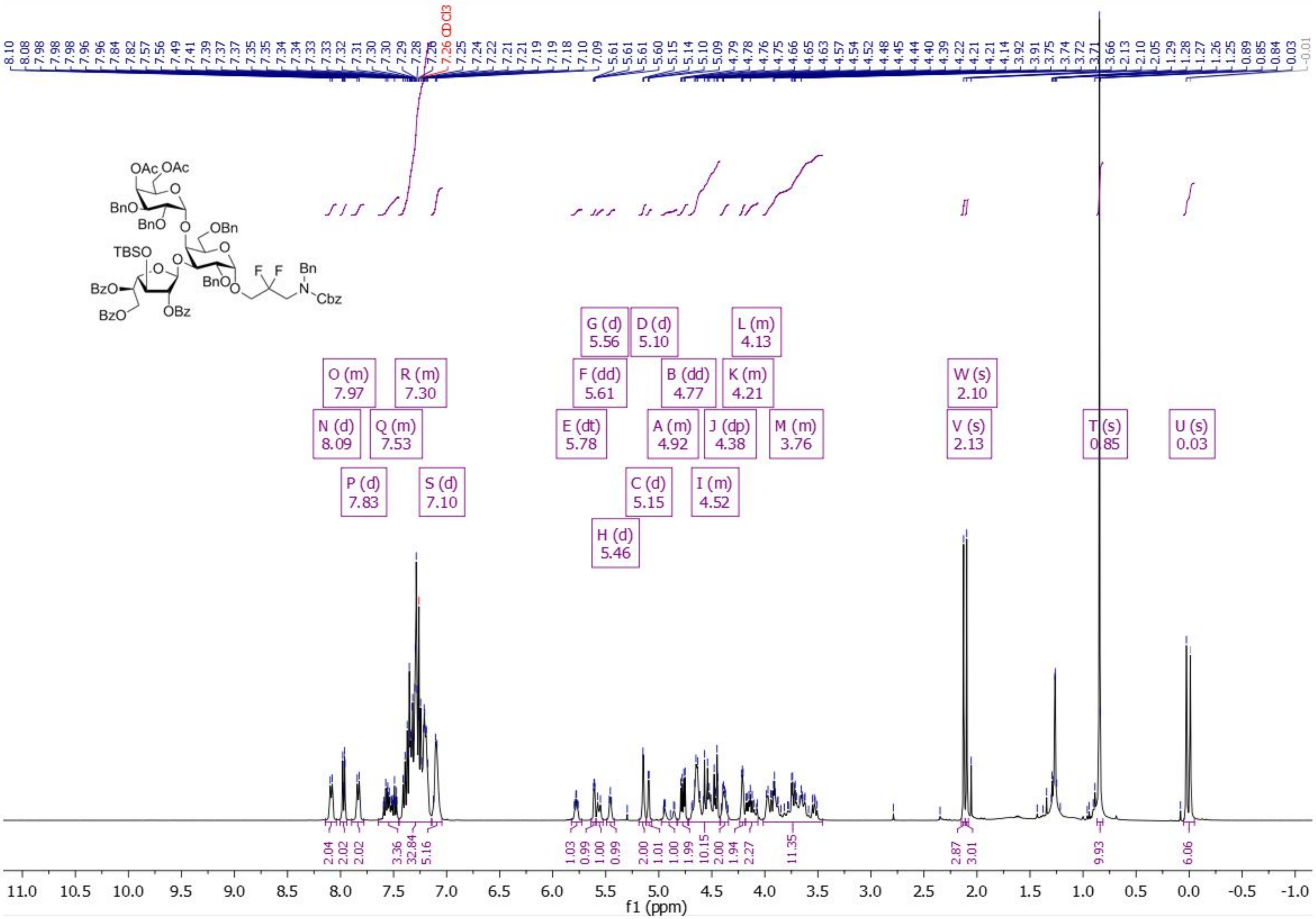

^{13}C-NMR of **18**

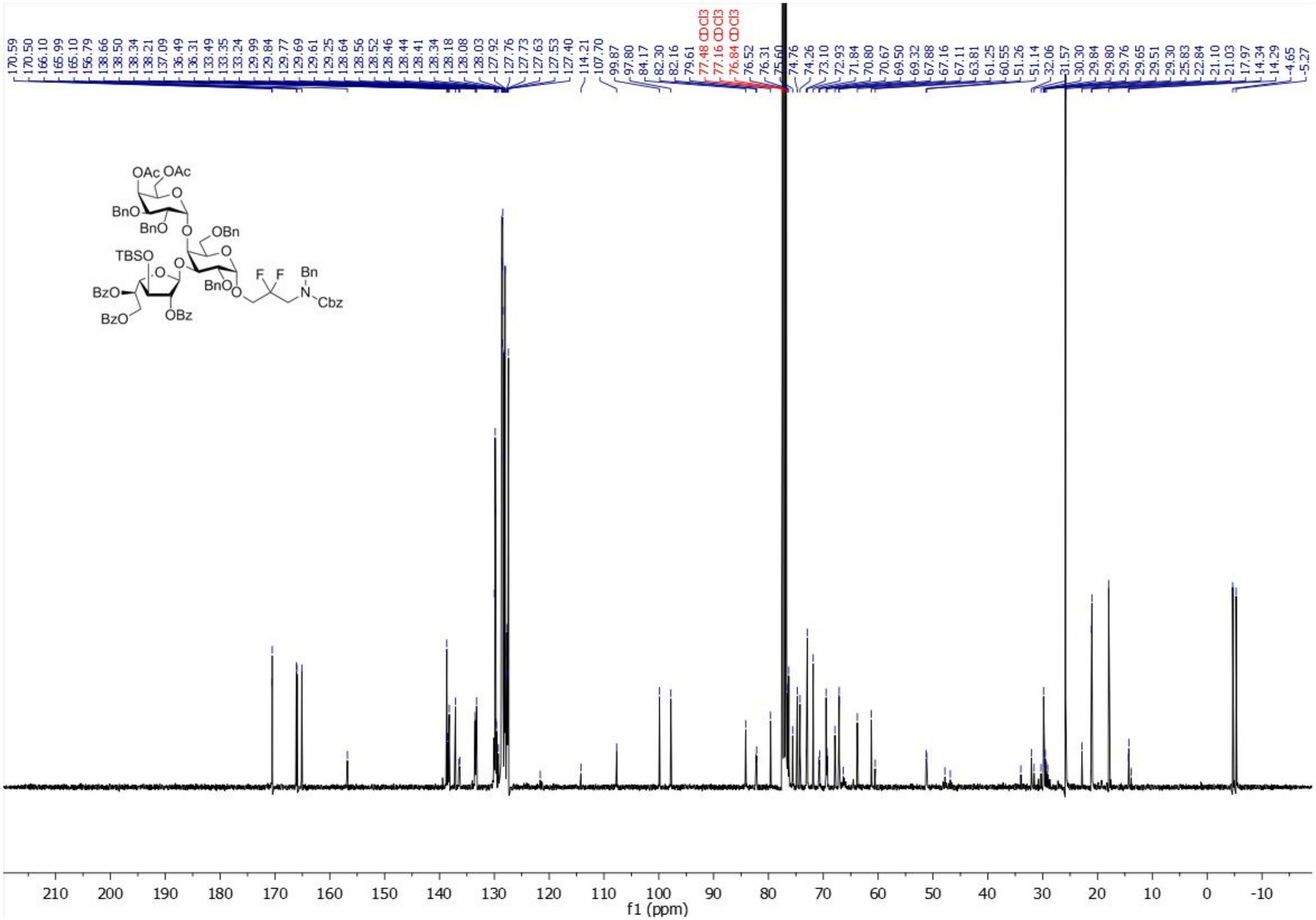
170.59
170.50
166.10
165.99
165.10
156.79
138.66
138.50
138.34
138.21
137.09
136.49
136.31
133.49
133.35
133.24
129.99
129.84
129.77
129.69
129.61
129.25
128.64
128.56
128.52
128.46
128.44
128.41
128.34
128.18
128.08
128.03
127.92
127.76
127.73
127.63
127.53
127.40
114.21
107.70
99.87
97.80
84.17
82.30
82.16
79.61
77.48 CDCl3
77.16 CDCl3
76.84 CDCl3
76.52
76.31
75.60
74.76
74.26
73.10
72.93
71.84
70.80
70.67
69.50
69.32
67.88
67.16
67.11
63.81
61.25
60.55
51.26
51.14
32.06
31.57
30.30
29.84
29.80
29.76
29.65
29.51
29.30
25.83
22.84
21.10
21.03
17.97
14.34
14.29
-4.65
-5.27
OAc OAc
BnO
BnO
O
OBn
TBSO
O
BzO
BzO
OBz
BnO
F F
Bn
N
Cbz
210 200 190 180 170 160 150 140 130 120 110 100 90 80 70 60 50 40 30 20 10 0 -10
f1 (ppm)

H-C HSQC of **18**

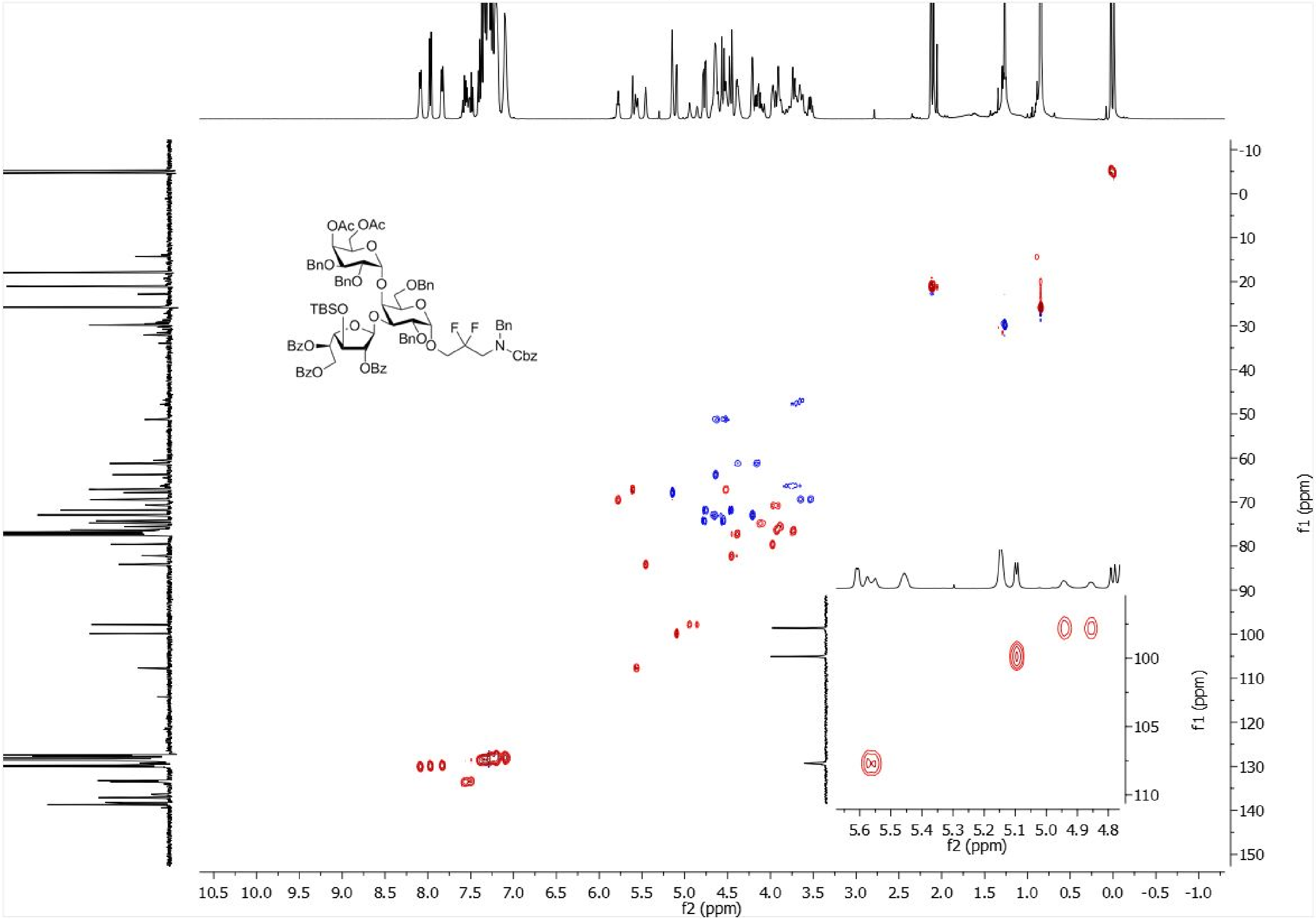
OAc OAc
O
BnO
BnO
O
OBn
O
TBSO
O
O
BnO
O
F F
Bn
N
Cbz
BzO
BzO
OBz
f1 (ppm)
f2 (ppm)

^{1}H-NMR of **20**

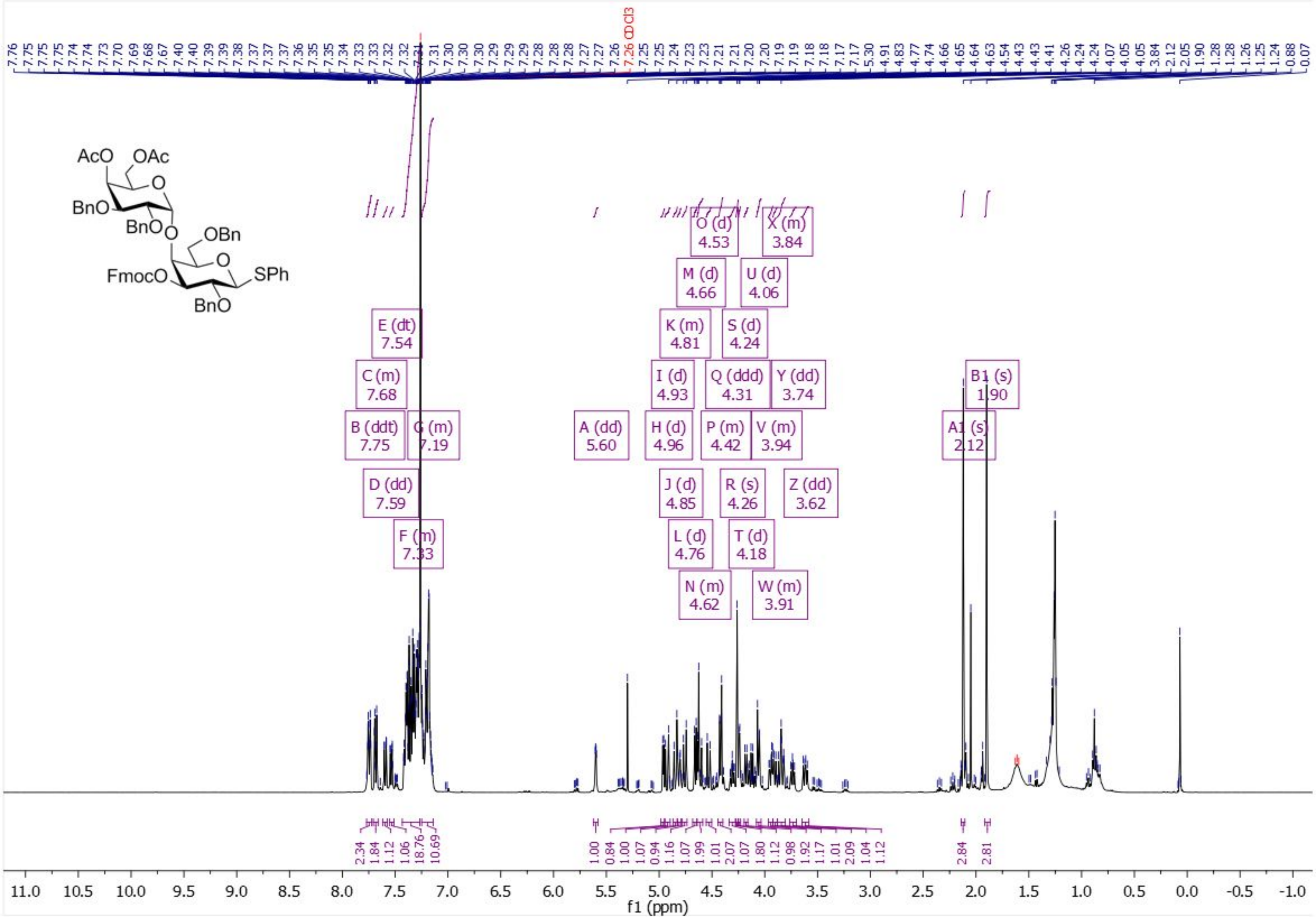

^{13}C-NMR of **20**

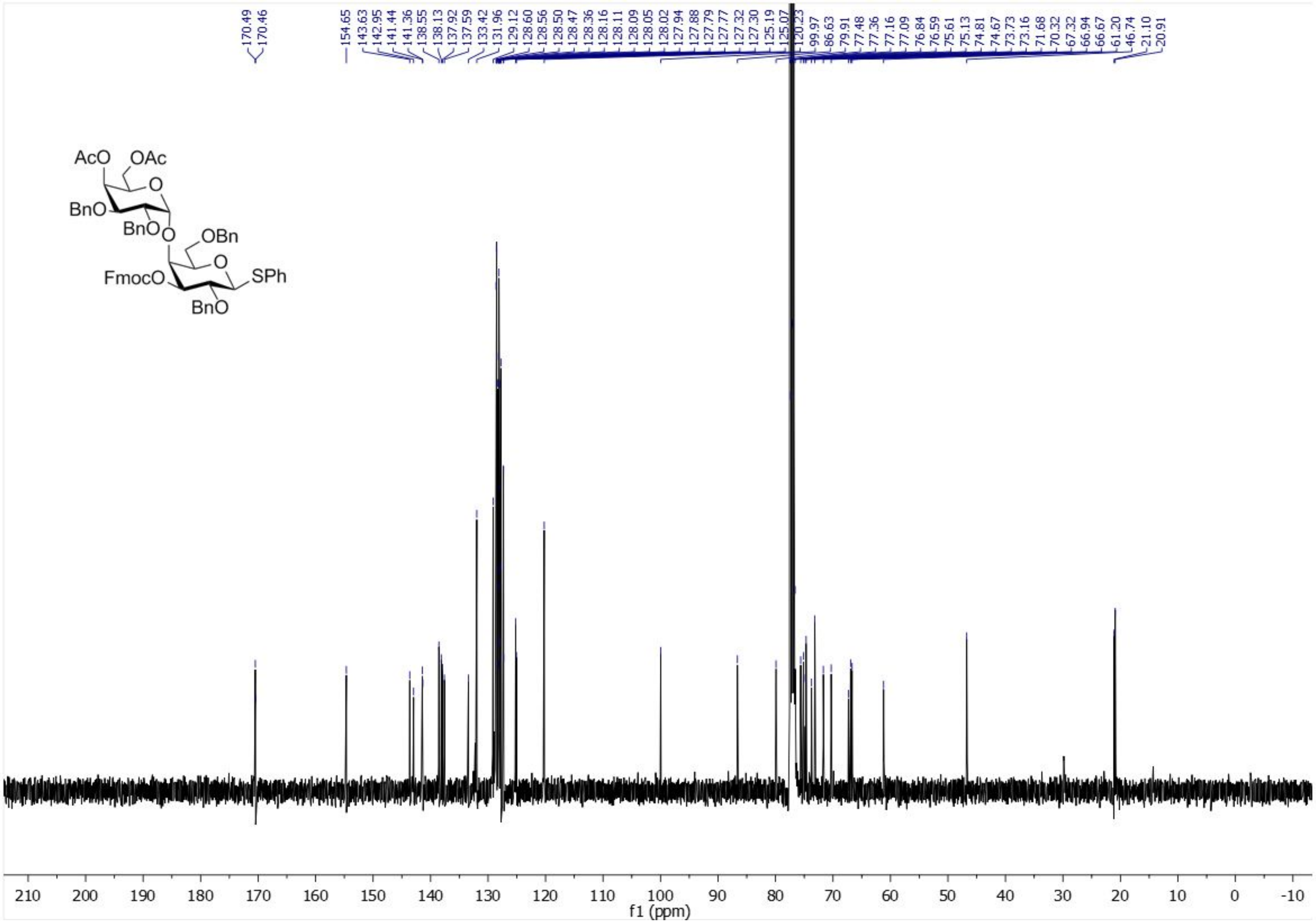
170.49
170.46
154.65
143.63
142.95
141.44
141.36
138.55
138.13
137.92
137.59
133.42
131.96
129.12
128.60
128.56
128.50
128.47
128.36
128.16
128.11
128.09
128.05
128.02
127.94
127.88
127.79
127.77
127.32
127.30
125.19
125.07
120.23
99.97
86.63
79.91
77.48
77.36
77.16
77.09
76.84
76.59
75.61
75.13
74.81
74.67
73.73
73.16
71.68
70.32
67.32
66.94
66.67
61.20
46.74
21.10
20.91
AcO
OAc
O
BnO
BnO
O
OBn
O
FmocO
SPh
BnO
210 200 190 180 170 160 150 140 130 120 110 100 90 80 70 60 50 40 30 20 10 0 -10
f1 (ppm)

H-C HSQC of **20**

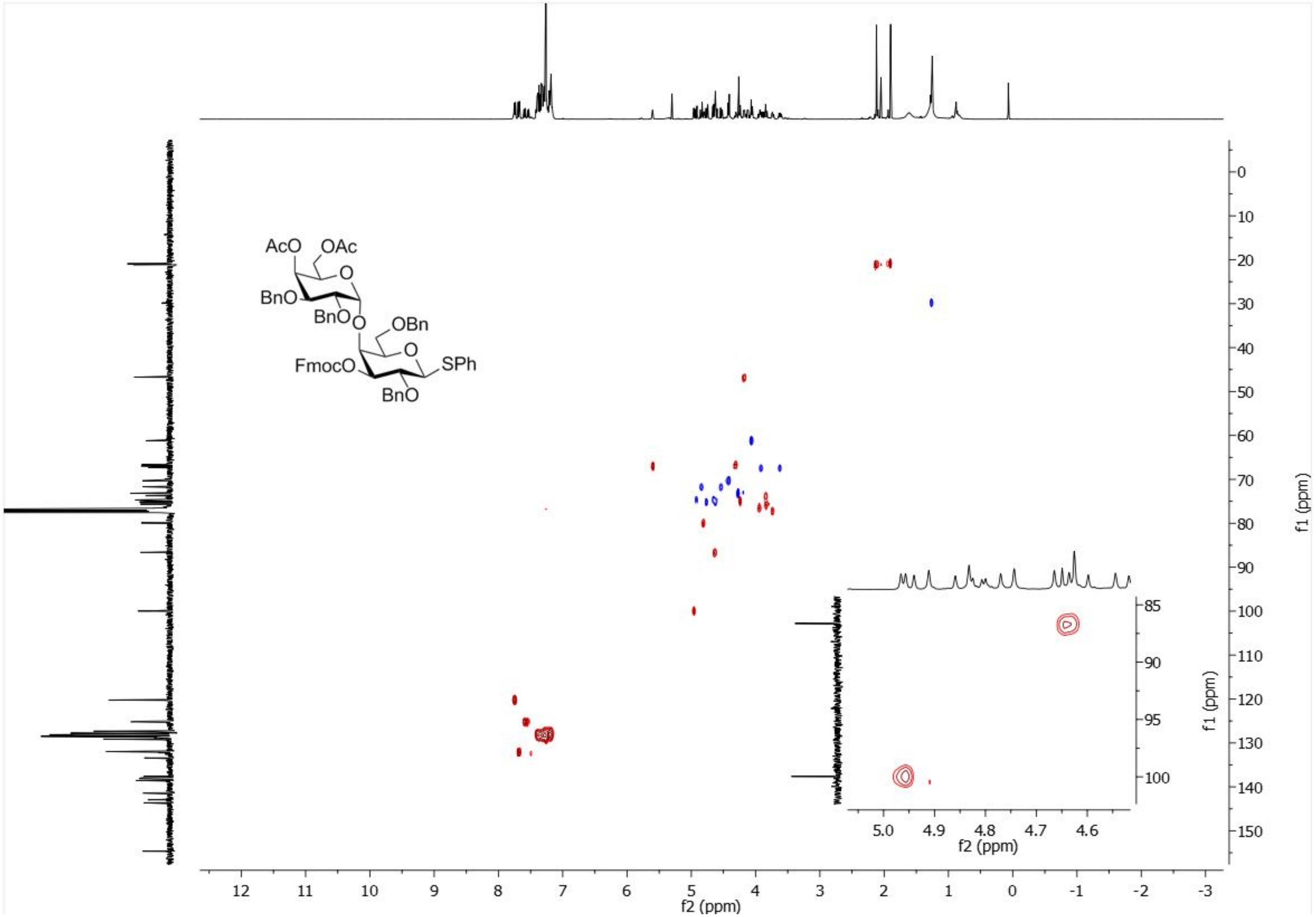

^{1}H-NMR of **21**

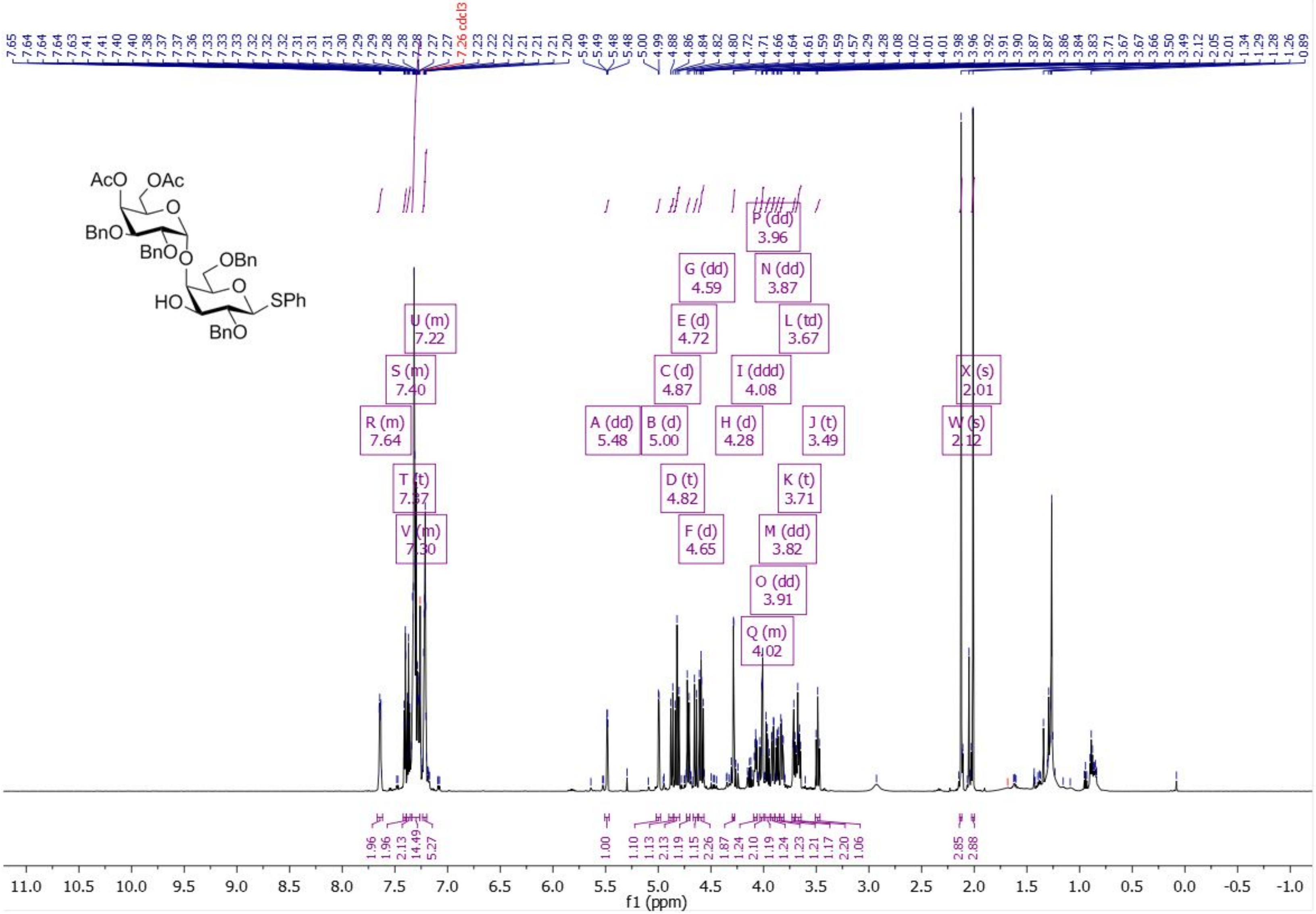
AcO OAc
O
BnO
BnO O
OBn
O
HO
SPh
BnO
7.26 cdcl3
R (m) 7.64
S (m) 7.40
T (t) 7.37
V (m) 7.30
U (m) 7.22
A (dd) 5.48
B (d) 5.00
C (d) 4.87
D (t) 4.82
E (d) 4.72
F (d) 4.65
G (dd) 4.59
H (d) 4.28
I (ddd) 4.08
Q (m) 4.02
P (dd) 3.96
O (dd) 3.91
N (dd) 3.87
M (dd) 3.82
K (t) 3.71
L (td) 3.67
J (t) 3.49
W (s) 2.12
X (s) 2.01
f1 (ppm)

^{13}C-NMR of **21**

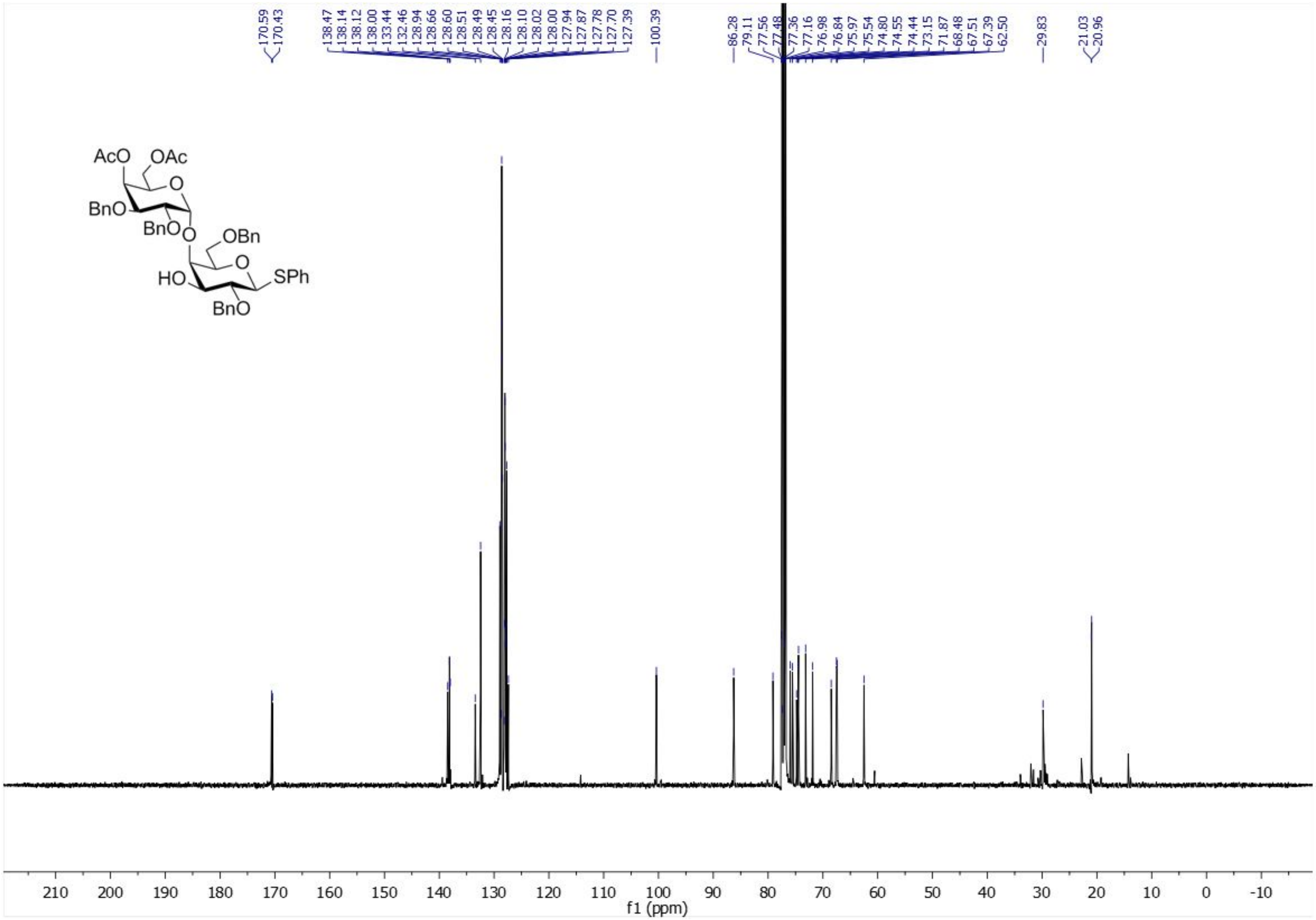
170.59
170.43
138.47
138.14
138.12
138.00
133.44
132.46
128.94
128.66
128.60
128.51
128.49
128.45
128.16
128.10
128.02
128.00
127.94
127.87
127.78
127.70
127.39
100.39
86.28
79.11
77.56
77.48
77.36
77.16
76.98
76.84
75.97
75.54
74.80
74.55
74.44
73.15
71.87
68.48
67.51
67.39
62.50
29.83
21.03
20.96
AcO
OAc
O
BnO
BnO
O
OBn
O
HO
SPh
BnO
210
200
190
180
170
160
150
140
130
120
110
100
90
80
70
60
50
40
30
20
10
0
-10
f1 (ppm)

H-C HSQC of **21**

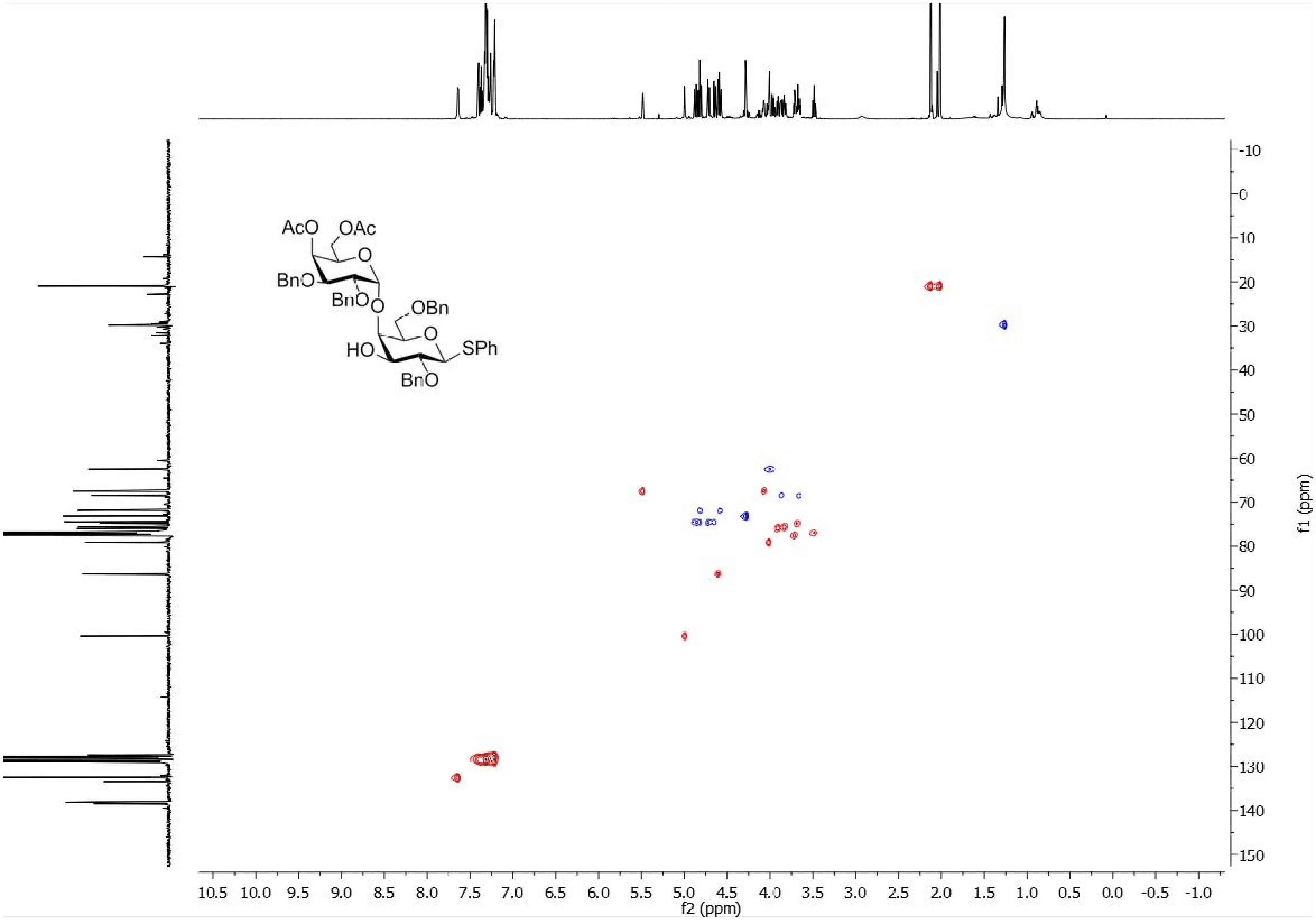

Coupled H-C HSQC of **21**

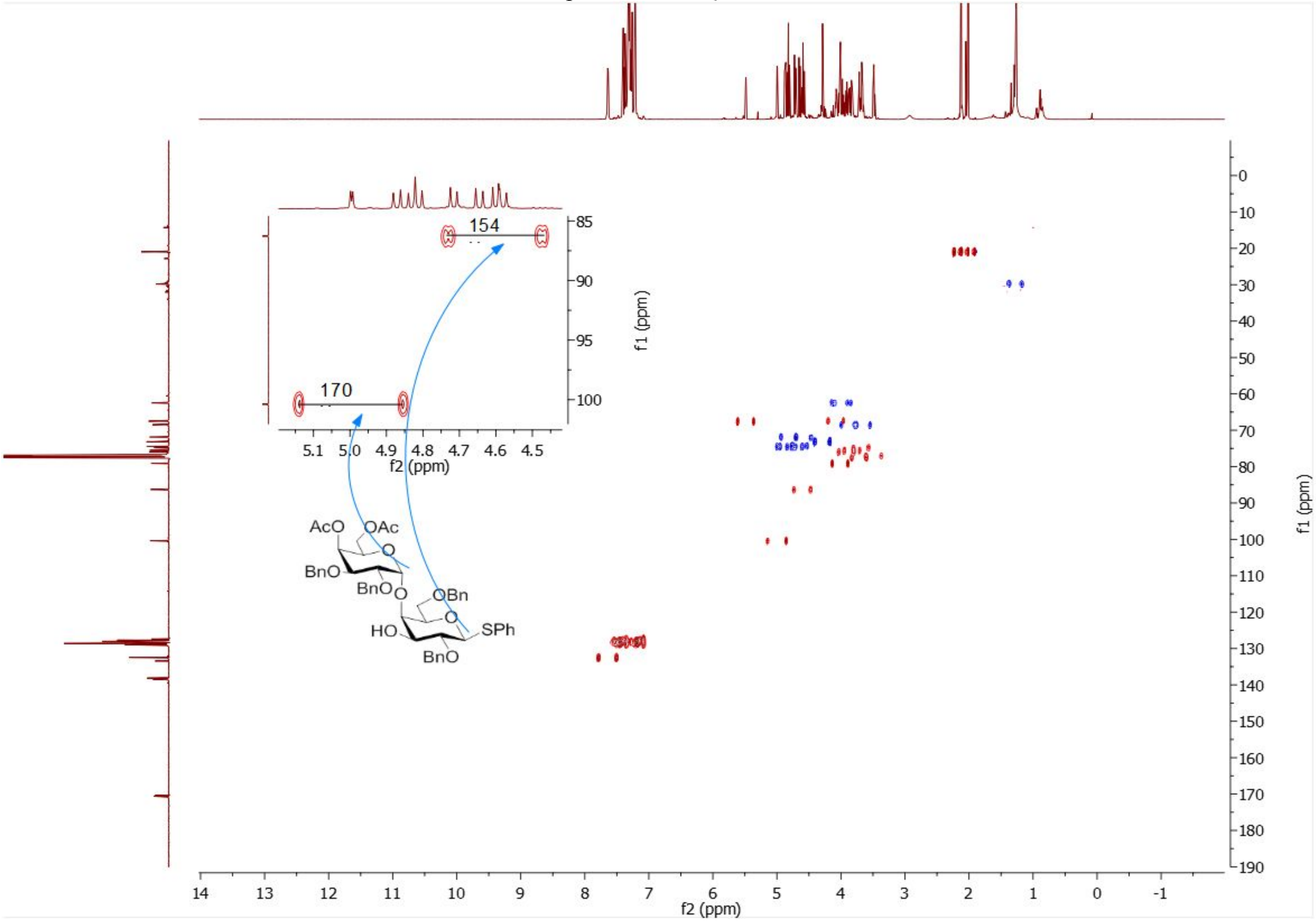

^{1}H-NMR of **S-1**

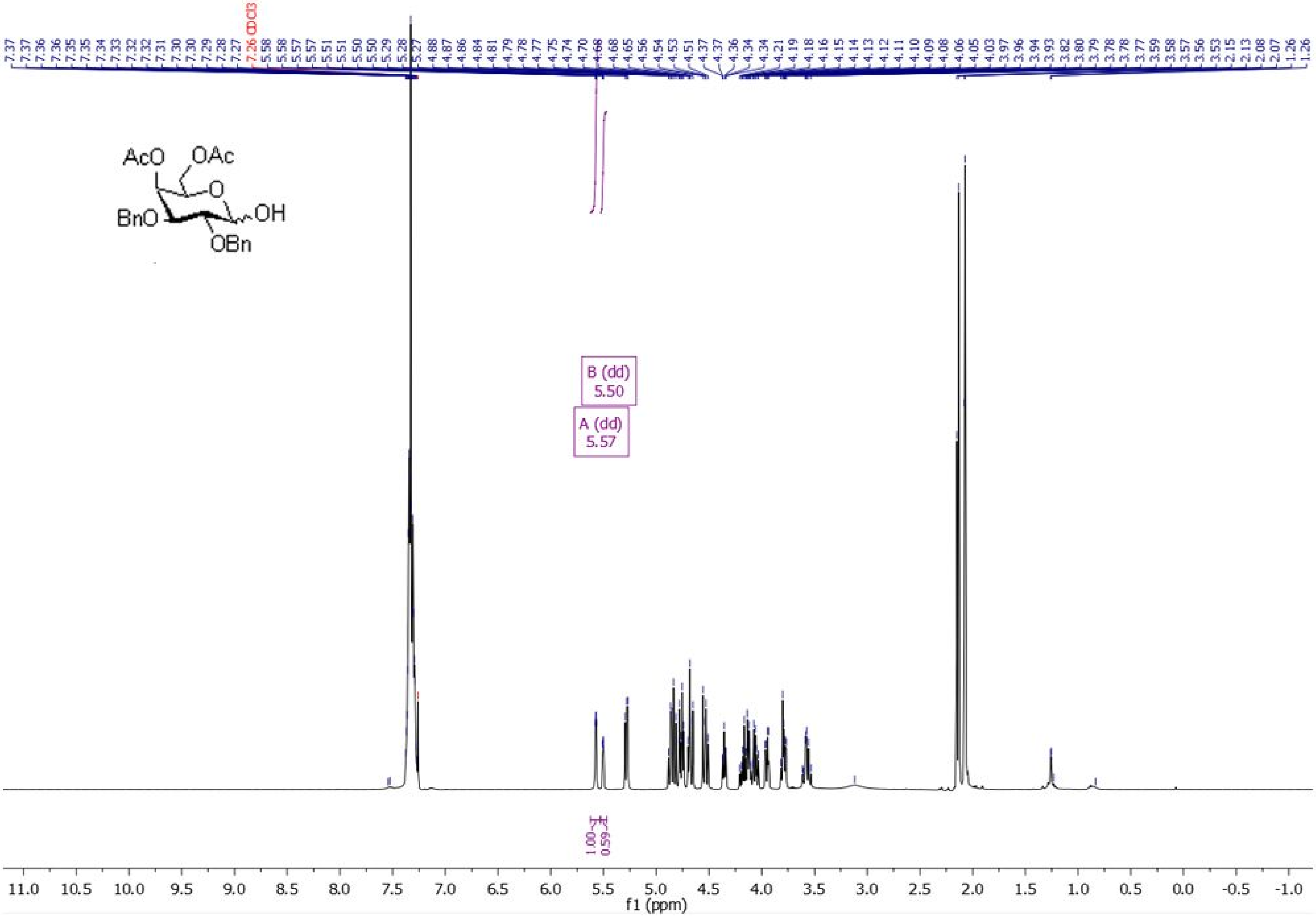
AcO
OAc
O
BnO
OH
OBn
B (dd)
5.50
A (dd)
5.57
1.00
0.59
11.0 10.5 10.0 9.5 9.0 8.5 8.0 7.5 7.0 6.5 6.0 5.5 5.0 4.5 4.0 3.5 3.0 2.5 2.0 1.5 1.0 0.5 0.0 -0.5 -1.0
f1 (ppm)

^{13}C-NMR of **S-1**

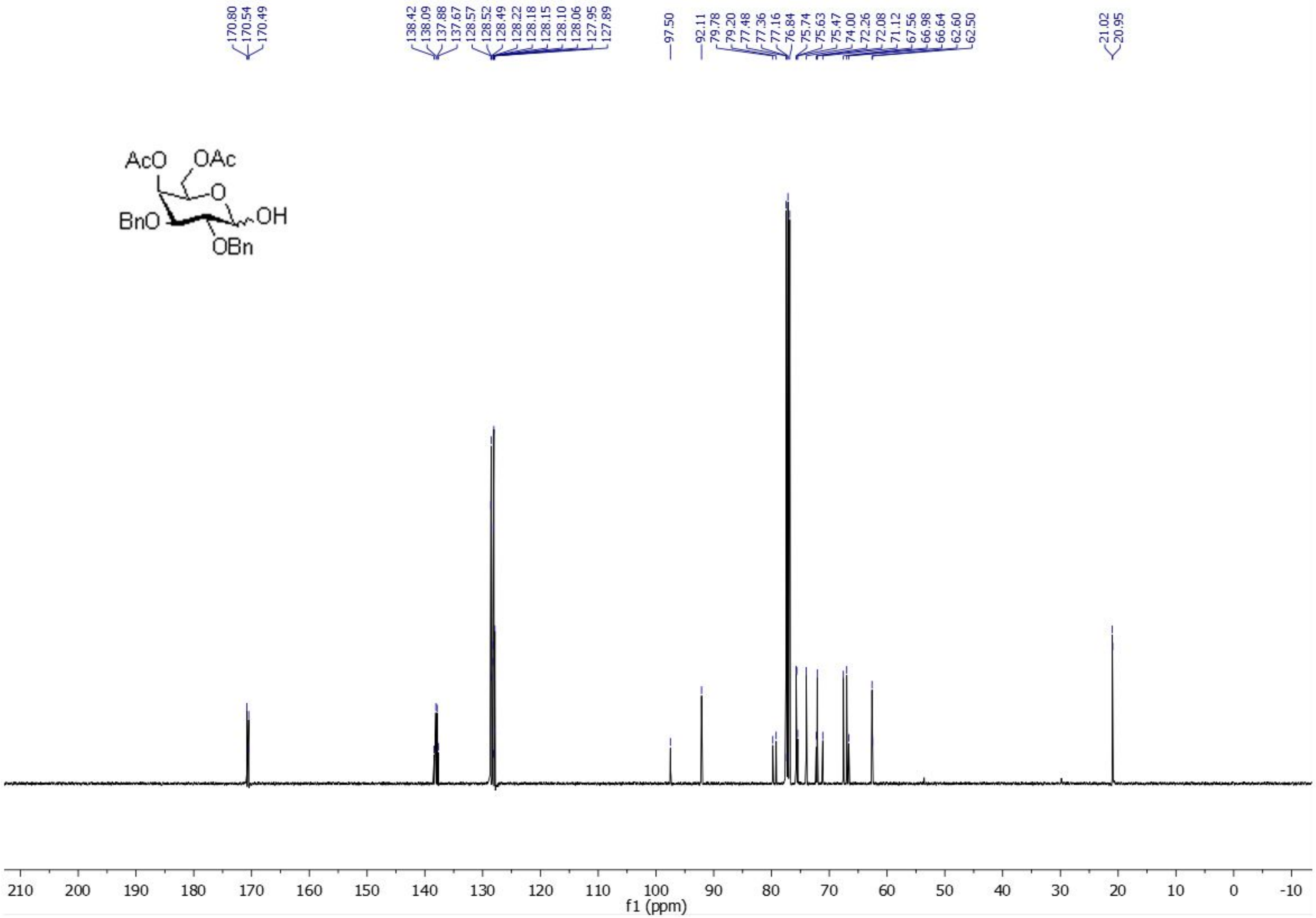
170.80
170.54
170.49
138.42
138.09
137.88
137.67
128.57
128.52
128.49
128.22
128.18
128.15
128.10
128.06
127.95
127.89
97.50
92.11
79.78
79.20
77.48
77.36
77.16
76.84
75.74
75.63
75.47
74.00
72.26
72.08
71.12
67.56
66.98
66.64
62.60
62.50
21.02
20.95
AcO
OAc
O
BnO
OH
OBn
210 200 190 180 170 160 150 140 130 120 110 100 90 80 70 60 50 40 30 20 10 0 -10
f1 (ppm)

H-C HSQC of **S-1**

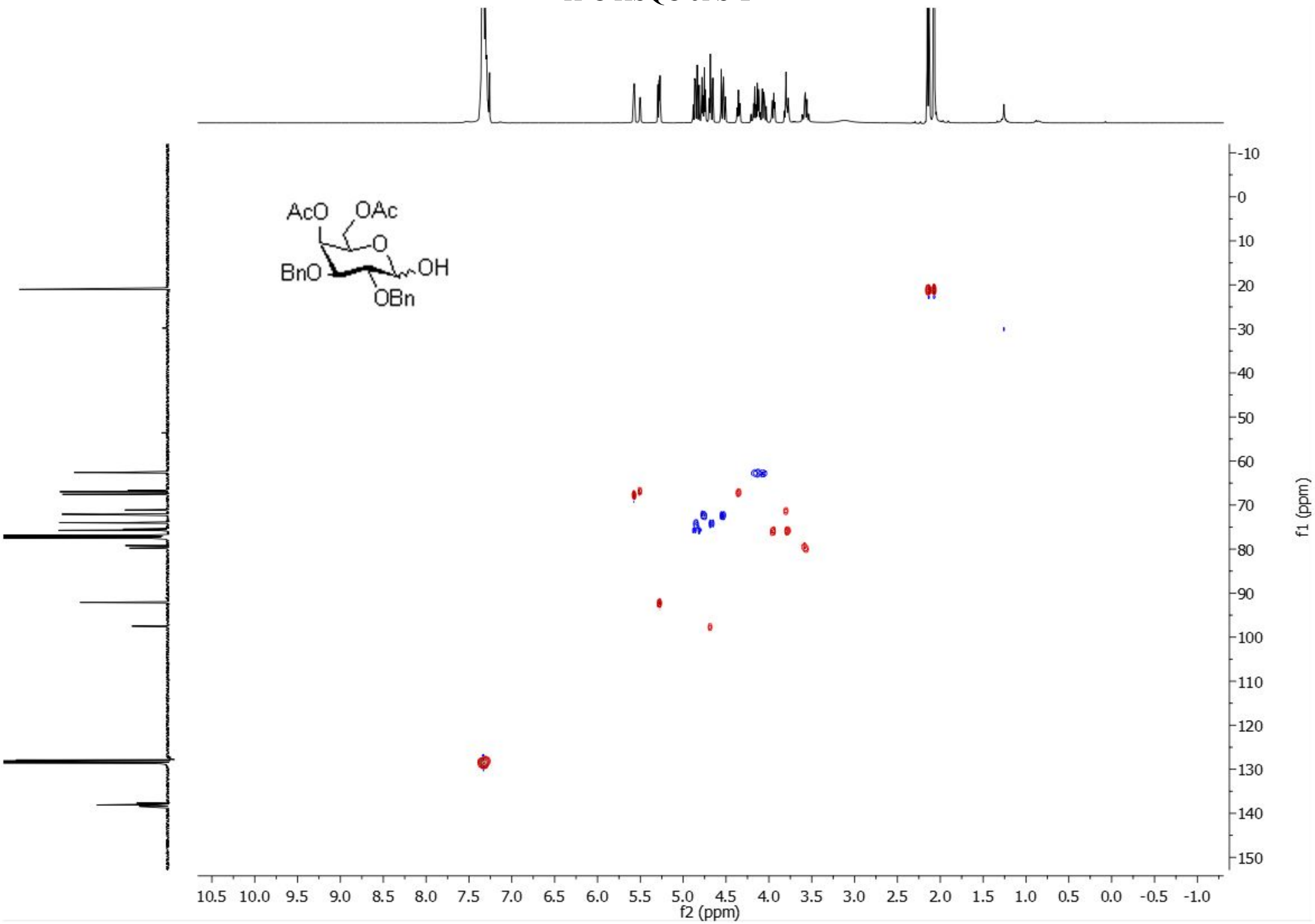

^{1}H-NMR of **19**

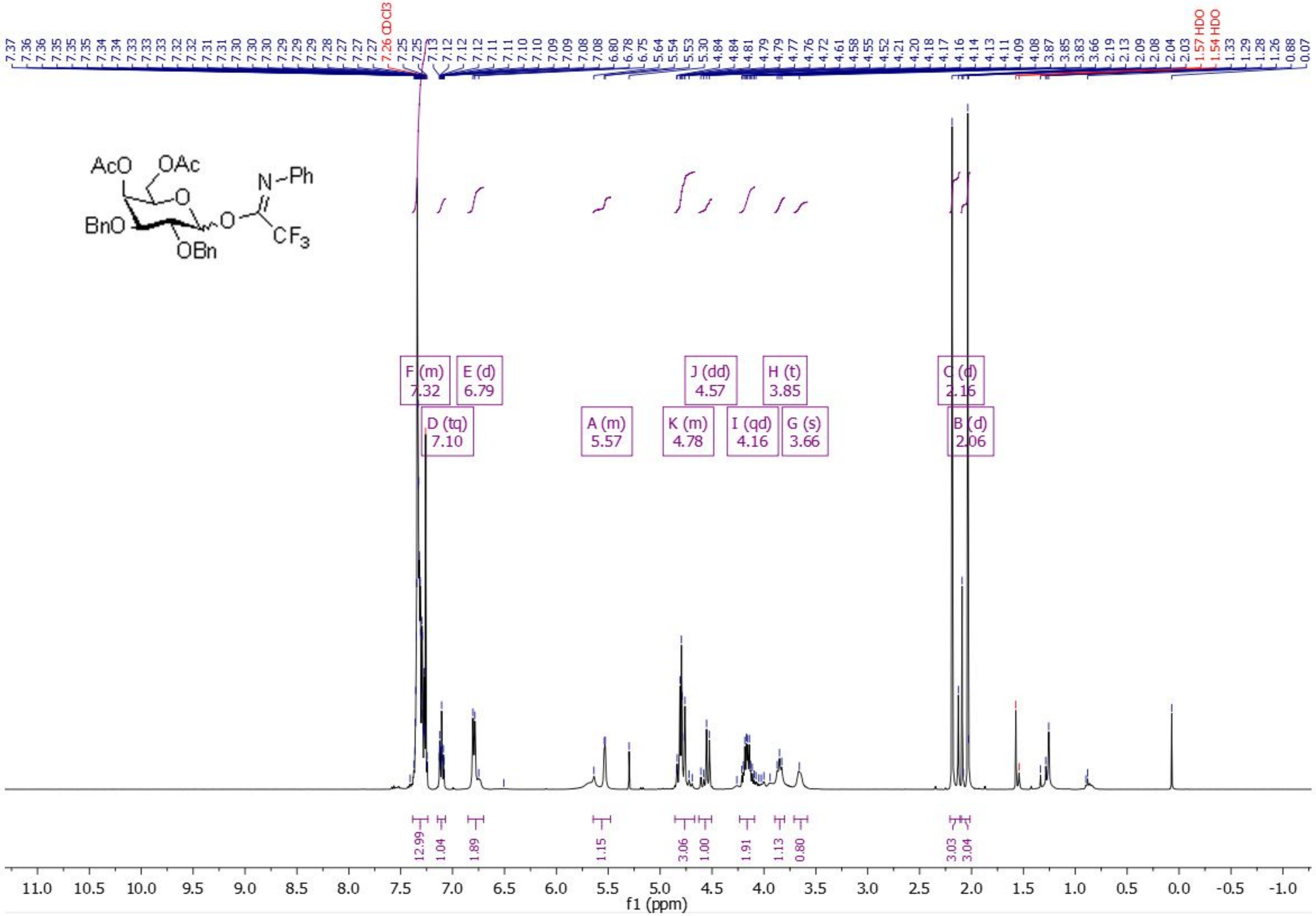

^{13}C-NMR of **19**

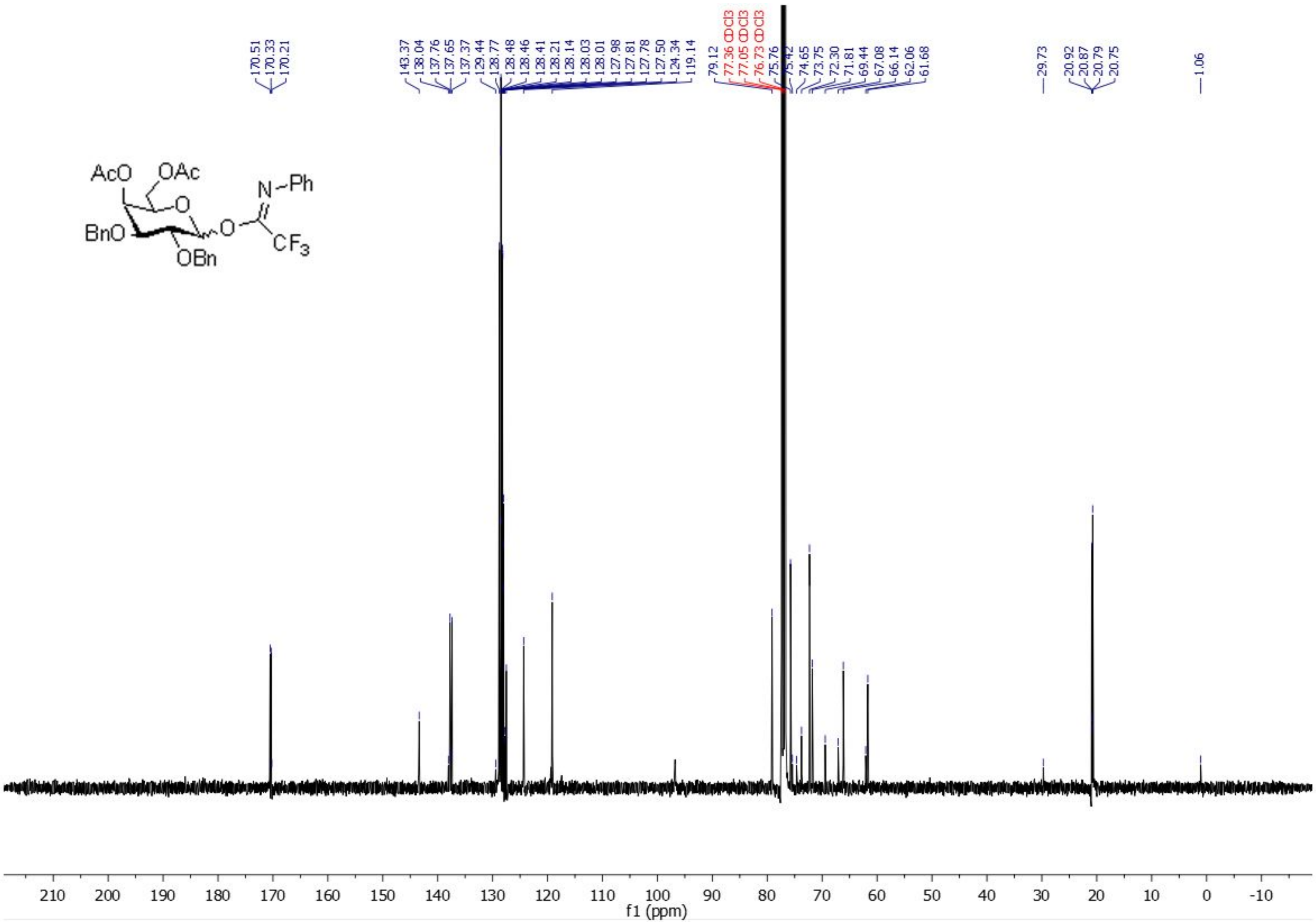
170.51
170.33
170.21
143.37
138.04
137.76
137.65
137.37
129.44
128.77
128.48
128.46
128.41
128.21
128.14
128.03
128.01
127.98
127.81
127.78
127.50
124.34
119.14
79.12
77.36 CDCl3
77.05 CDCl3
76.73 CDCl3
75.76
75.42
74.65
73.75
72.30
71.81
69.44
67.08
66.14
62.06
61.68
29.73
20.92
20.87
20.79
20.75
1.06
AcO
OAc
N-Ph
O
BnO
O
CF3
OBn
210 200 190 180 170 160 150 140 130 120 110 100 90 80 70 60 50 40 30 20 10 0 -10
f1 (ppm)

H-C HSQC of **19**

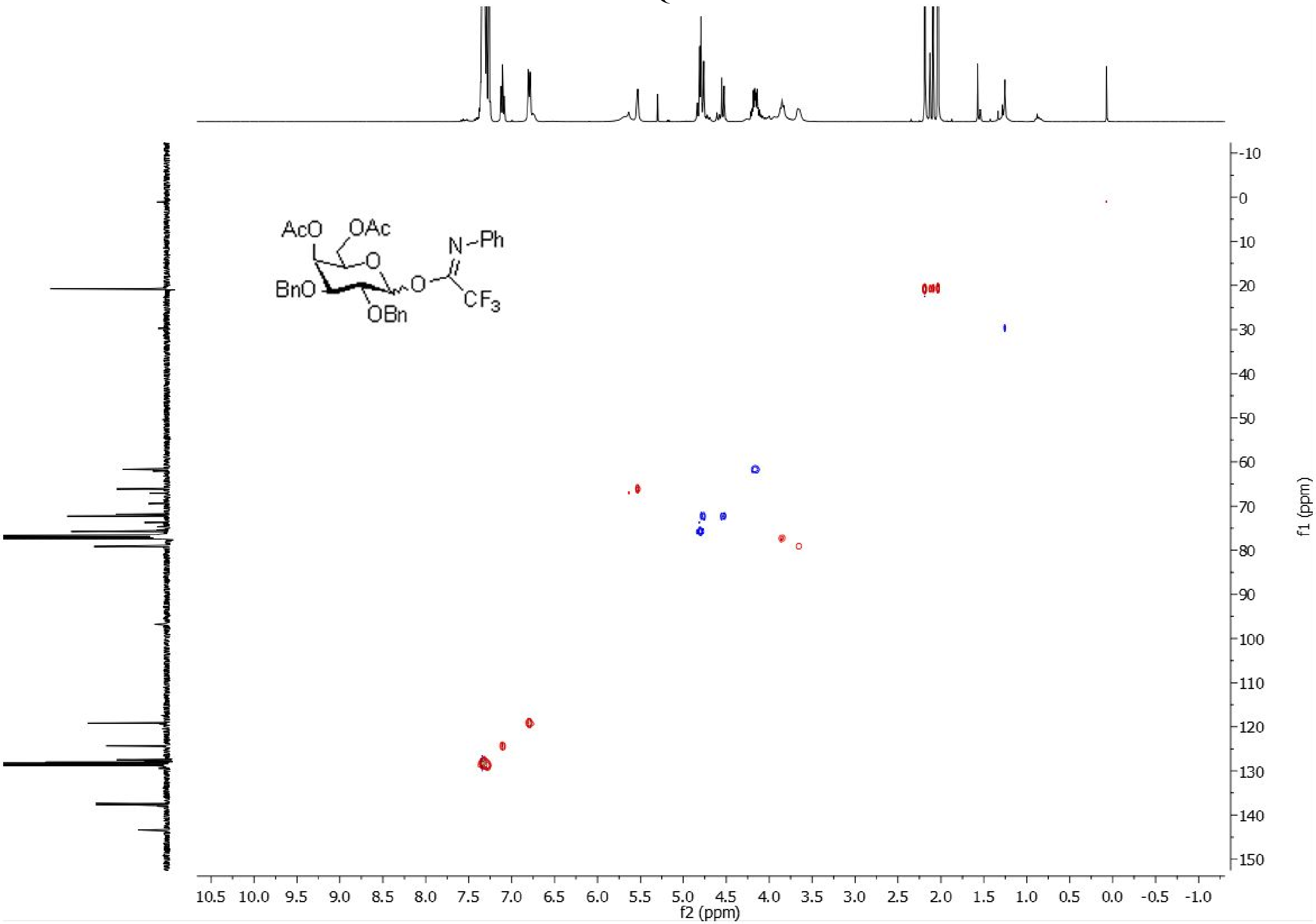

^{1}H-NMR of **4**

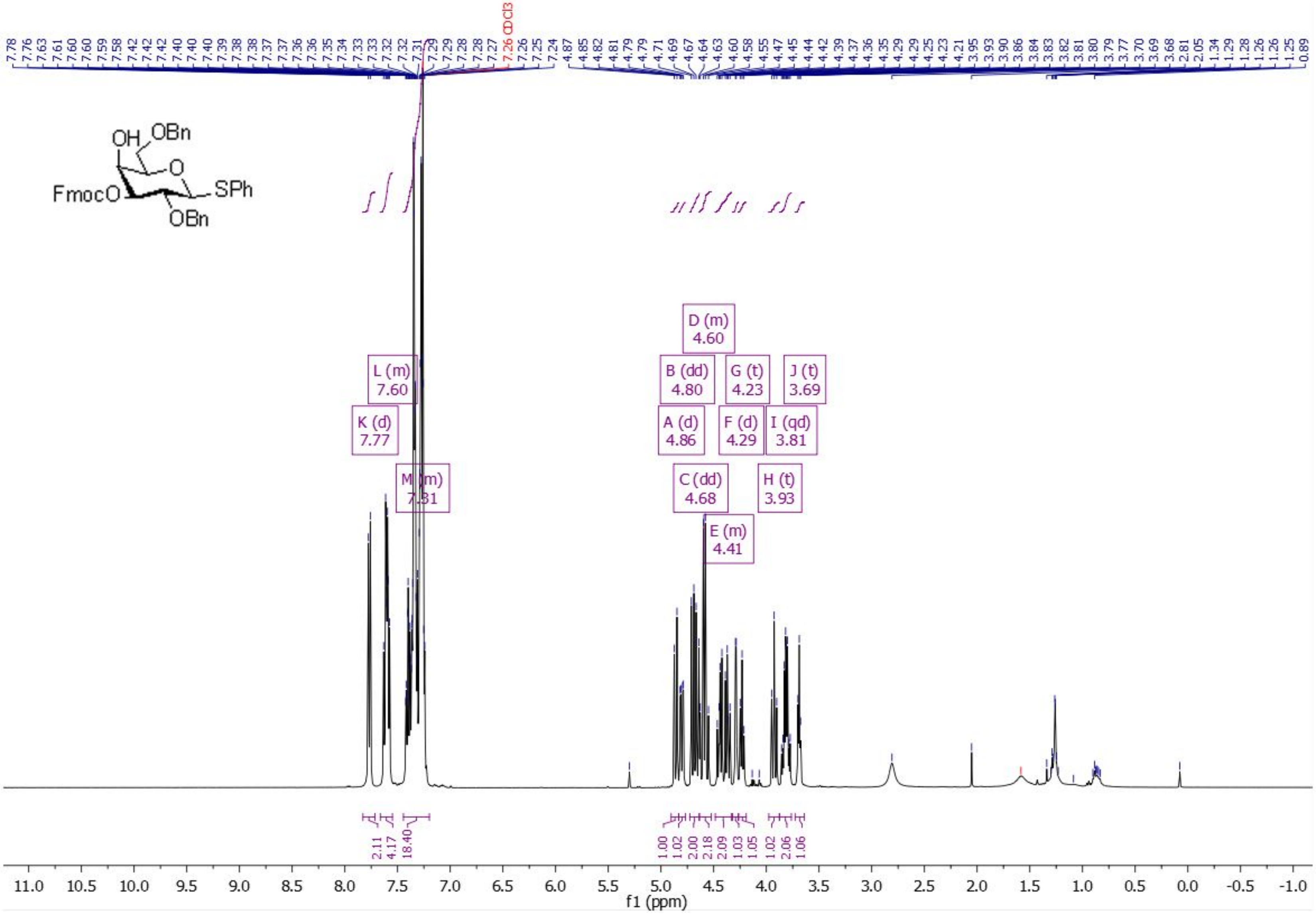

^{13}C-NMR of **4**

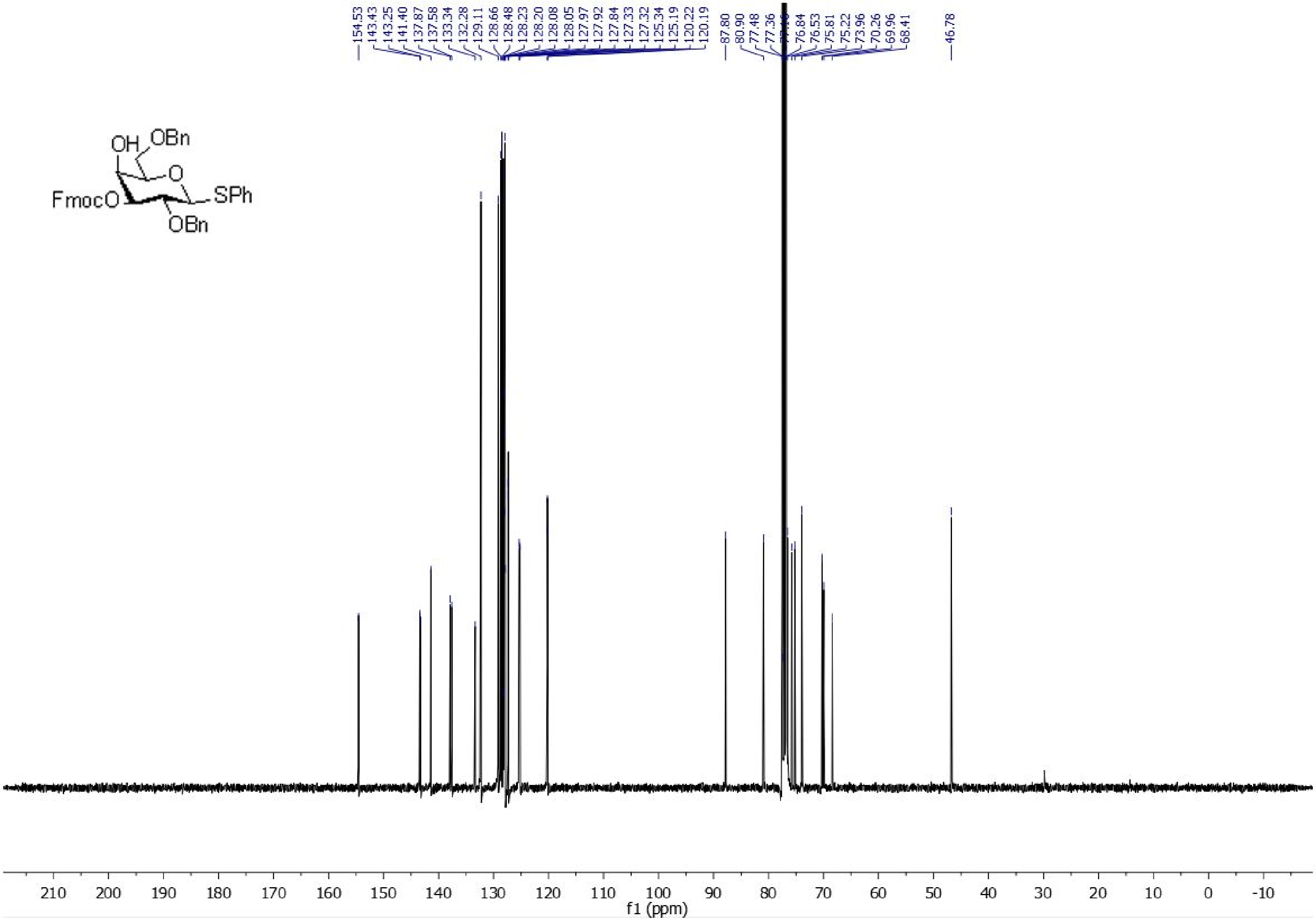
154.53
143.43
143.25
141.40
137.87
137.58
133.34
132.28
129.11
128.66
128.48
128.23
128.20
128.08
128.05
127.97
127.92
127.84
127.33
127.32
125.34
125.19
120.22
120.19
87.80
80.90
77.48
77.36
76.84
76.53
75.81
75.22
73.96
70.26
69.96
68.41
46.78
OH
OBn
O
FmocO
SPh
OBn
210 200 190 180 170 160 150 140 130 120 110 100 90 80 70 60 50 40 30 20 10 0 -10
f1 (ppm)

H-C HSQC of **4**

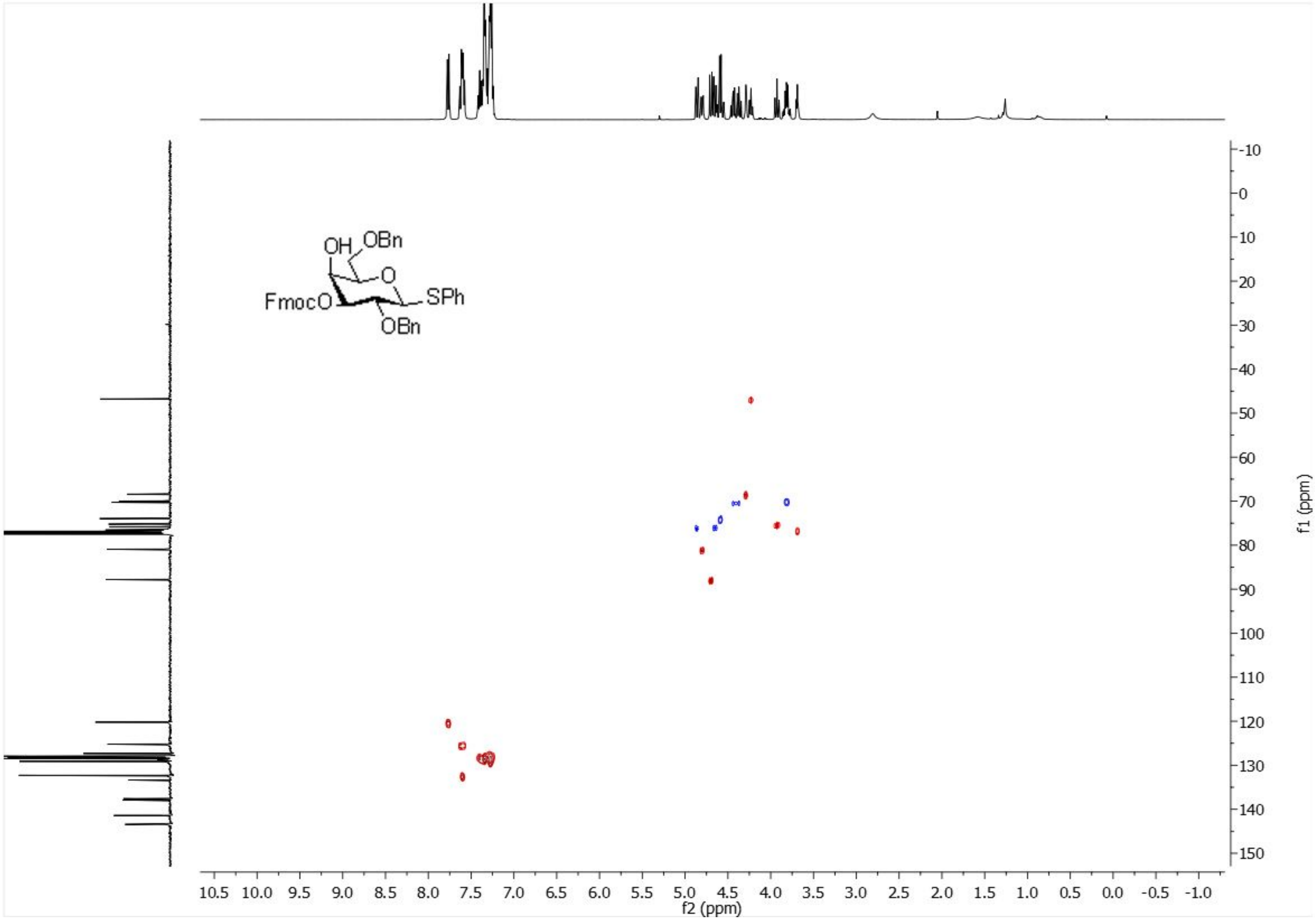

^{1}H-NMR of **17**

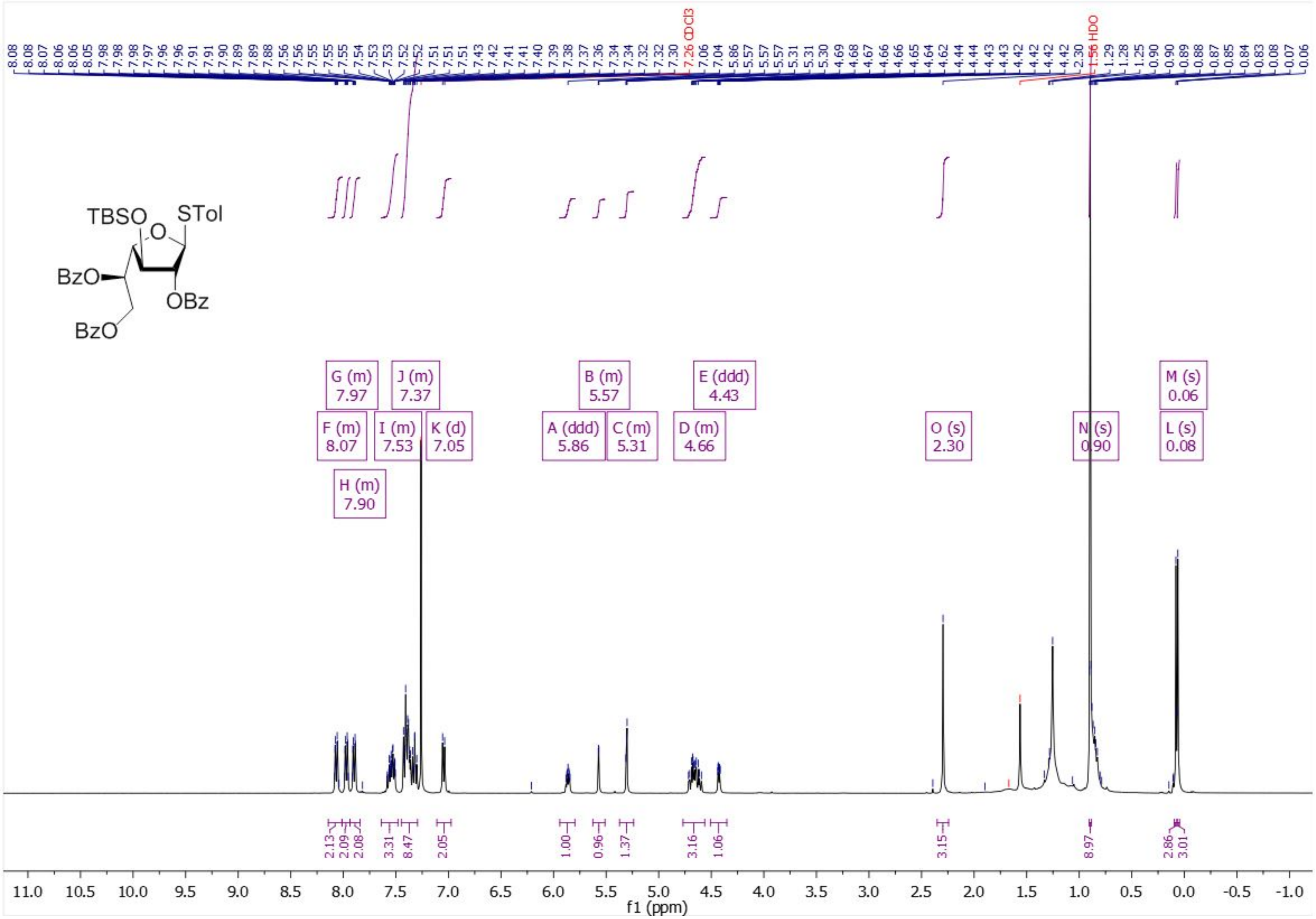
TBSO
STol
O
BzO
OBz
BzO
G (m) 7.97
J (m) 7.37
B (m) 5.57
E (ddd) 4.43
M (s) 0.06
F (m) 8.07
I (m) 7.53
K (d) 7.05
A (ddd) 5.86
C (m) 5.31
D (m) 4.66
O (s) 2.30
N (s) 0.90
L (s) 0.08
H (m) 7.90
7.26 CDCl3
1.56 HDO
f1 (ppm)

^{13}C-NMR of **17**

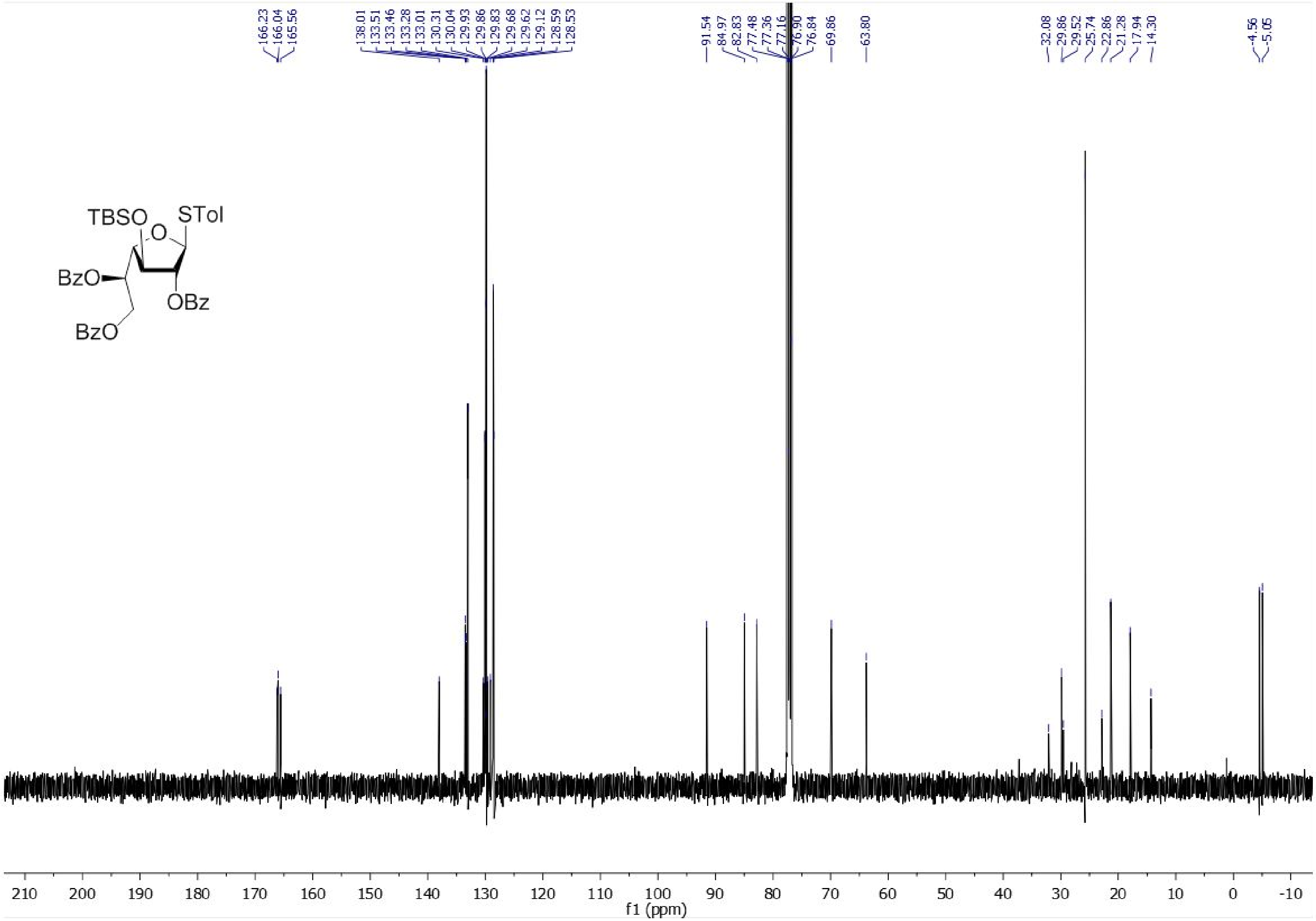
166.23
166.04
165.56
138.01
133.51
133.46
133.28
133.01
130.31
130.04
129.93
129.86
129.83
129.68
129.62
129.12
128.59
128.53
91.54
84.97
82.83
77.48
77.36
77.16
76.90
76.84
69.86
63.80
32.08
29.86
29.52
25.74
22.86
21.28
17.94
14.30
-4.56
-5.05
TBSO
STol
O
BzO
OBz
BzO
210 200 190 180 170 160 150 140 130 120 110 100 90 80 70 60 50 40 30 20 10 0 -10
f1 (ppm)

H-C HSQC of **17**

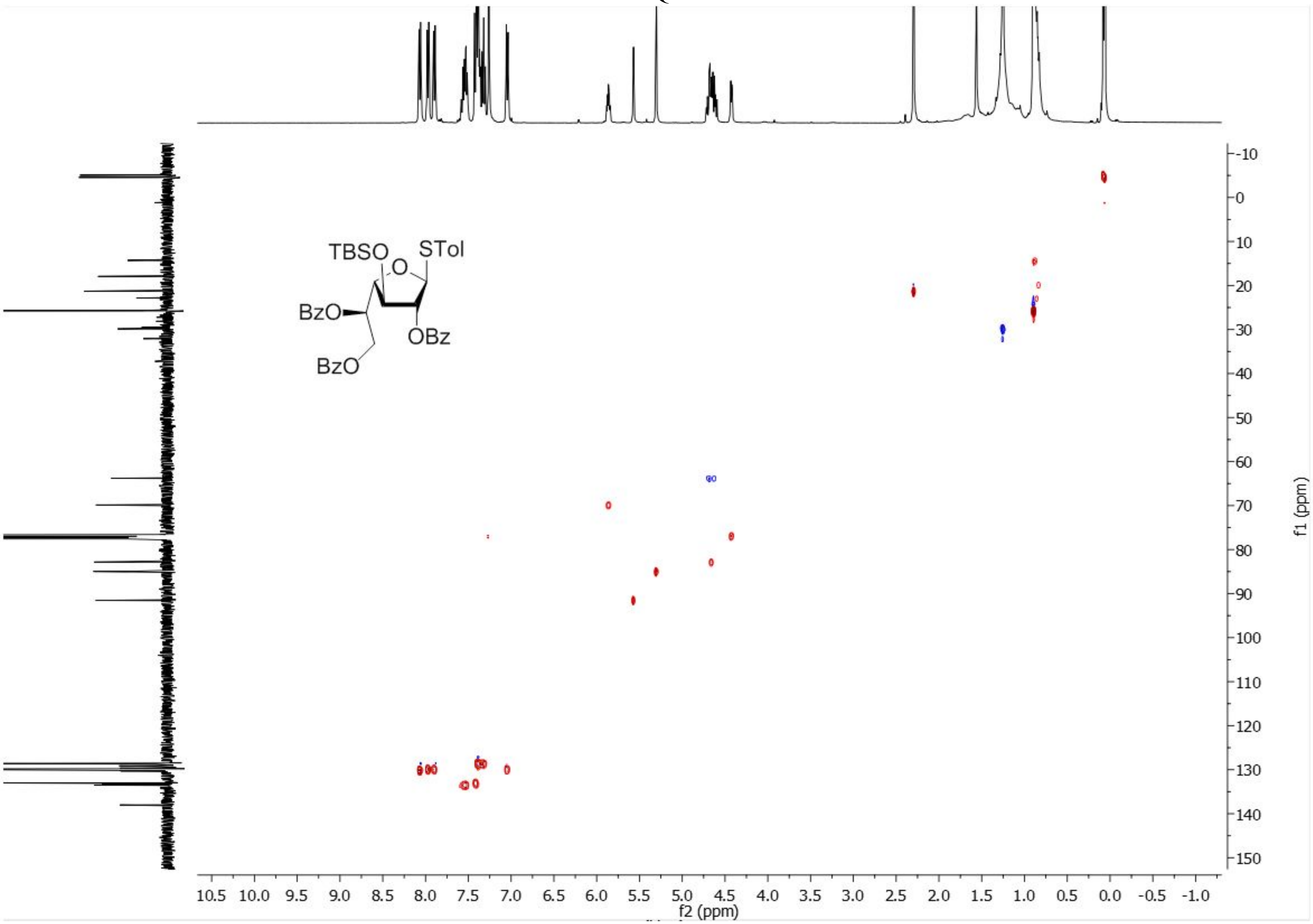

Supplement: Supplementary file 2 — ja4c13972_si_002.pdf [file ja4c13972_si_002.pdf]
